# A new versatile primer set targeting a short fragment of the mitochondrial COI region for metabarcoding metazoan diversity: application for characterizing coral reef fish gut contents

**DOI:** 10.1186/1742-9994-10-34

**Published:** 2013-06-14

**Authors:** Matthieu Leray, Joy Y Yang, Christopher P Meyer, Suzanne C Mills, Natalia Agudelo, Vincent Ranwez, Joel T Boehm, Ryuji J Machida

**Affiliations:** 1 Laboratoire d'Excellence "CORAIL", USR 3278 CRIOBE CNRS-EPHE, CBETM de l’Université de Perpignan, 66860, Perpignan Cedex, France; 2 Department of Invertebrate Zoology, National Museum of Natural History, Smithsonian Institution, P.O. Box 37012, MRC-163, Washington, DC 20013-7012, USA; 3 National Human Genome Research Institute, National Institutes of Health, Bethesda, Maryland, USA; 4 Montpellier SupAgro (UMR AGAP), Montpellier, France; 5 Biology Department, City College of New York, New York, NY 10031, USA; 6 The Graduate Center, City University of New York, New York, NY 10016, USA; 7 Biodiversity Research Center, Academia Sinica, Taipei, Taiwan

**Keywords:** Second generation sequencing, DNA barcoding, Mini-barcode, Mitochondrial marker, Trophic interactions, Food web

## Abstract

**Introduction:**

The PCR-based analysis of homologous genes has become one of the most powerful approaches for species detection and identification, particularly with the recent availability of Next Generation Sequencing platforms (NGS) making it possible to identify species composition from a broad range of environmental samples. Identifying species from these samples relies on the ability to match sequences with reference barcodes for taxonomic identification. Unfortunately, most studies of environmental samples have targeted ribosomal markers, despite the fact that the mitochondrial Cytochrome c Oxidase subunit I gene (COI) is by far the most widely available sequence region in public reference libraries. This is largely because the available versatile (“universal”) COI primers target the 658 barcoding region, whose size is considered too large for many NGS applications. Moreover, traditional barcoding primers are known to be poorly conserved across some taxonomic groups.

**Results:**

We first design a new PCR primer within the highly variable mitochondrial COI region, the “mlCOIintF” primer. We then show that this newly designed forward primer combined with the “jgHCO2198” reverse primer to target a 313 bp fragment performs well across metazoan diversity, with higher success rates than versatile primer sets traditionally used for DNA barcoding (i.e. LCO1490/HCO2198). Finally, we demonstrate how the shorter COI fragment coupled with an efficient bioinformatics pipeline can be used to characterize species diversity from environmental samples by pyrosequencing. We examine the gut contents of three species of planktivorous and benthivorous coral reef fish (family: Apogonidae and Holocentridae). After the removal of dubious COI sequences, we obtained a total of 334 prey Operational Taxonomic Units (OTUs) belonging to 14 phyla from 16 fish guts. Of these, 52.5% matched a reference barcode (>98% sequence similarity) and an additional 32% could be assigned to a higher taxonomic level using Bayesian assignment.

**Conclusions:**

The molecular analysis of gut contents targeting the 313 COI fragment using the newly designed mlCOIintF primer in combination with the jgHCO2198 primer offers enormous promise for metazoan metabarcoding studies. We believe that this primer set will be a valuable asset for a range of applications from large-scale biodiversity assessments to food web studies.

## Introduction

Biological diversity often poses a major challenge for ecologists who seek to understand ecological processes or conduct biomonitoring programs. Environmental samples commonly contain a high taxonomic diversity of small-sized organisms (e.g., meiofauna in marine benthic sediments [1]), with numerous specimens lacking diagnostic morphological characters (i.e. larval stages in plankton tows [2]) or partially digested organisms in gut or faecal contents [3]), making it difficult to identify species within a reasonable timeframe and with sufficient accuracy [4]. Yet, DNA-based community analyses have offered some alternatives to traditional methods and have become even more promising with the availability of ultrasequencing platforms now supplanting cloning. Taxon detection from bulk samples can be achieved using PCR amplification followed by deep sequencing of homologous gene regions. Sequences are then compared to libraries of reference barcodes for taxonomic identification. This so-called “metabarcoding” approach [5] has been used as a powerful means to understand the diversity and distribution of meiofauna [6]. It has also been found to be an effective tool for assessing the diversity of insects collected from traps [7] and characterize the diet of predators [8-11] and herbivores [12,13] through analysis of their feces or gut content. Nevertheless, metabarcoding is still a relatively new approach, and both methodological and analytical improvements are necessary to further expand its range of applications [7,14].

The success of a metabarcoding analysis is particularly contingent upon the primer set used and the target loci, because they will determine the efficiency and accuracy of taxon detection and identification. In general, primers should preferentially target hypervariable DNA regions (for high resolution taxonomic discrimination) for which extensive libraries of reference sequences are available (for taxonomic identification). Furthermore, primers should preferentially target short DNA fragments (e.g., < 400 bp) to maximize richness estimates [15,16] and increase the probability of recovering DNA templates that are more degraded (sheared), such as samples preserved for extended periods of time [17] or prey items in the gut and faecal contents of predators [18,19]. The taxonomic coverage of the primer set will then depend upon the question addressed. For example, when the goal is to describe the diet of specialised predators (i.e. insects consumed by bats [20,21]) or more generally to describe the diversity and composition of a specific functional group (i.e. nematodes in sediments [6]), “group-specific” primers will be effective. Alternatively, when the goal is to obtain a comprehensive analysis of samples containing species from numerous phyla (as most environmental samples do), primers should target a locus found universally across all animals or plants.

Despite the inherent difficulty of designing versatile primers (also referred to as broad-range or universal primers), several sets are readily available to amplify nuclear and mitochondrial gene fragments across animals. For example, there are primers to amplify short fragments of the nuclear 18S and 28S ribosomal markers [22,23], but these regions evolve slowly and may underestimate diversity [24-27]. Versatile primers have also recently become available to target a short fragment of the mitochondrial 12S gene [28], a region with high rates of molecular evolution suitable for species delineation and identification, but taxonomic reference databases are currently highly limited for this marker. The mitochondrial Cytochrome c Oxidase I gene (COI) has been adopted as the standard ‘taxon barcode’ for most animal groups [29] and is by far the most represented in public reference libraries. As of January 2013, the Barcode of Life Database included COI sequences from >1,800,000 specimens belonging to >160,000 species collected among all phyla across all ecosystems. However, versatile primers are only available to amplify the barcoding region of 658 bp [30,31] and are known to be poorly conserved across nematodes [6,26], gastropods [31] and echinoderms [32] among others. A single attempt was made at designing a versatile primer to amplify a shorter “mini-barcode” COI region [17], but it has received limited use due to large numbers of mismatches in the priming site that affects its efficiency across a broad range of taxa [33].

In the first part of this paper, we use an extensive library of COI barcodes provided by the Moorea BIOCODE project, an “All Taxa Biotic Inventory” (http://www.mooreabiocode.org), to locate a conserved priming site internal to the highly variable 658 bp COI region. The newly designed internal primer is combined with a modified version of the classic reverse barcoding primer HCO2198 proposed by Folmer et al. (1994) [30] (“jgHCO2198” - [34] to target a 313 bp COI region. We test the effectiveness of the primer set across 287 disparate taxa from 30 phyla and we compare its performance against versatile primer sets commonly employed for DNA barcoding.

In the second part of this paper, we demonstrate how the new COI primer set coupled with an effective bioinformatics pipeline allows high throughput DNA-based characterization of prey diversity from the gut contents of coral reef fish species with three distinct feeding modes. Analysis of predator’s gut or faecal contents is one of the promising applications of the DNA metabarcoding approach. Efficient prey detection combined with high-resolution prey identification offers the potential for improving our understanding of food webs, animal feeding behaviour [14] and prey distribution [35,36]. Previously, due to the large amplicon size, COI was often considered a non-suitable marker ( [8,19,37], reviewed in [14]). We propose that this new primer set will be a powerful asset for understanding various ecological processes and conducting biomonitoring programs.

## Material and methods

### COI primer design and performance test

#### Primer design

**Figure 1:**
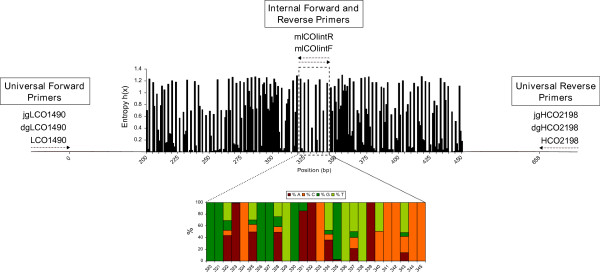
**Design of the “mlCOIint” forward and reverse complements within the highly variable COI fragment.** A total of 6643 COI sequences, spanning 17 phyla (provided by the Moorea BIOCODE project) were aligned and the entropy h(x) plotted to visualize the level of variability at each position. h(x) = 0 when the site is conserved across all sequences (e.g., 100% A). h(x) is at a maximum when each nucleotide occurs at equal frequency. We also present the proportion of each nucleotide between sites 320 and 345, the region where the primers were designed.

We aimed to design a versatile PCR primer within the 658 bp COI barcoding region which could be used in combination with a published primer commonly used for DNA barcoding (i.e. LCO1490 or HCO2198 [30]) to target a short DNA fragment. The Moorea BIOCODE project provided an alignment of 6643 COI sequences belonging to ~3877 marine taxa, mostly coral reef associated species (up to five specimens per morphospecies) spanning 17 animal phyla (sequences available in BOLD, projects MBMIA, MBMIB and MBFA). The information content [entropy h(x)] at each position of the alignment was plotted using BioEdit [38] to locate more conserved regions within the 658 bp COI barcoding fragment (Figure 1). A site with limited variation was located between positions 320 and 345 of the 658 bp COI region (Figure 1). The forward primer “mlCOIintF” and its reverse complement “mlCOIintR” (Table 1) were designed herein and used for further performance testing.

**Table 1:** COI primers used in this study

**Primer label**	**Sequence (5' - 3')**	**Reference**
LCO1490	GGTCAACAAATCATAAAGATATTGG	[30]
HCO2198	TAAACTTCAGGGTGACCAAAAAATCA	[30]
dgLCO1490	GGTCAACAAATCATAAAGAYATYGG	[31]
dgHCO2198	TAAACTTCAGGGTGACCAAARAAYCA	[31]
jgLCO1490	TITCIACIAAYCAYAARGAYATTGG	[34]
jgHCO2198	TAIACYTCIGGRTGICCRAARAAYCA	[34]
Uni-MinibarF1	CAAAATCATAATGAAGGCATGAGC	[17]
Uni-MinibarR1	TCCACTAATCACAARGATATTGGTAC	[17]
mlCOIintF	GGWACWGGWTGAACWGTWTAYCCYCC	herein
mlCOIintR	GGRGGRTASACSGTTCASCCSGTSCC	herein

#### Primer performance

Genomic DNA was provided by the Moorea BIOCODE project for 287 specimens belonging to 30 animal phyla in order to carry out amplification tests (list of taxa in Additional file 1). Eight phyla were represented by more than five specimens and were the most common phyla from BIOCODE collections. These samples were organized in three 96 well plates.

We conducted preliminary tests to determine which primer combination performs best across a wide range of phyla to amplify a short size COI fragment. To test this, 47 specimens belonging to 11 phyla (rows 10 and 11 of each of the three plates - Additional file 1) were selected. We used the following primer combinations to target a 313 bp COI fragment (Figure 1): (1) mlCOIintF with HCO2198, (2) mlCOIintF with dgHCO2198, (3) mlCOIintF with jgHCO2198; and the following primer combinations to target a 319 bp COI fragment: (4) LCO1490 with mlCOIintR, (5) dgLCO1490 with mlCOIintR, (6) jgLCO1490 with mlCOIintR. It is important to note that dgHCO2198 and jgHCO2198 are degenerate versions of HCO2198 with the identical priming site, as dgLCO1490 and jgLCO1490 are to LCO1490 (see Table 1 for primer sequences and sources). PCR amplification was performed in a total volume of 20 μl with 0.6 μl of 10 μM of each universal forward and reverse primers, 0.2 μl of Biolase *taq* polymerase (Bioline) 5 U.μl^-1^, 0.8 μl of 50 mM Mg^2+^, 1 μl of 10 μM dNTP and 1 μl of genomic DNA. Because of the high level of degeneracy in primer sequences, we used a “touchdown” PCR profile to minimize the probability of non-specific amplifications. We carried out 16 initial cycles: denaturation for 10s at 95°C, annealing for 30s at 62°C (−1°C per cycle) and extension for 60s at 72°C, followed by 25 cycles at 46°C annealing temperature. Success of PCR amplifications was checked on 1.5% agarose gels. A clear single band of expected length indicated success whereas the absence of a band, the presence of multiple bands or the presence of a single band of incorrect size meant PCR failure. The primer set providing the best results was kept for further tests.

Secondly, the performance at amplifying the short COI fragment across the diversity of 285 templates was compared to the performance of existing COI primer sets targeting the 658 bp COI region commonly used for DNA barcoding, LCO1490 with HCO2198, as well as their degenerate versions dgLCO1490 with dgHCO2190 and jgLCO1490 with jgHCO2198. We also evaluated the performance of the mini-barcode primers Uni-MinibarF1 with Uni-MinibarR1 that were designed to amplify a short 130 bp COI fragment. For each primer set we used optimal reagent concentrations and thermocycler profiles found in the literature [17,31]. PCR products of the short 313 bp COI fragment were sequenced by Sanger sequencing.

### Pyrosequencing of fish gut contents

#### Specimen collection and gut content extraction

Nine adult specimens of the cardinal fish species, *Nectamia savayensis* (Order: Perciformes; Family: Apogonidae; total length = 59-83 mm), three specimens of soldierfish, *Myripristis berndti* (Order: Beryciformes; Family: Holocentridae; total length = 114-143 mm), and four specimens of the squirrelfish, *Sargocentron microstoma* (Order: Beryciformes; Family: Holocentridae; total length = 148-161 mm) were collected by spear-fishing on the 9th of August 2010, two hours after sunset in the lagoon of the North shore of Moorea Island, French Polynesia (17°30’S, 149°50’W). The three nocturnal fish species vary in their feeding mode and habitat use: *N. savayensis* occurs in the water column between two and three meters and is strictly planktivorous; *M. berndti* was collected from near reef crevices at four meters and consumes both planktonic and benthic prey; *S. microstoma* is also a benthic predator but preys upon larger benthic invertebrates [39,40]. Approval was granted from our institutional animal ethics committee, le Centre National de la Recherche Scientifique (CNRS), for sacrificing and subsequently dissecting fish (Permit Number: 006725). None of the fish species are on the endangered species list and no specific authorization was required from the French Polynesian government for collection.

Fish were preserved in cold 50% ethanol in the field. Their digestive systems were dissected within 2 hours in the laboratory and preserved in 80% ethanol at −20°C. After storage for 2 months, total genomic DNA was extracted from the total prey mixture contained in the digestive track using QIAGEN® DNeasy Blood & Tissue individual columns. Genomic DNA was purified using the MOBIO PowerClean DNA clean-up kit to prevent interference with PCR inhibitors.

#### Design of predator-specific blocking primers

Gut contents of semi-digested prey homogenate contain highly degraded prey DNA mixed with abundant high-quality DNA of the predator itself. Therefore, predator DNA co-amplification may prevent or bias prey recovery if no preventive measure is taken [41-43]. Therefore, we included predator-specific annealing blocking primers at ten times the concentration of versatile primers (tailed mlCOIintF and jgHCO2198, see below) in all PCR amplifications. Blocking primers are modified primers that overlap with one of the versatile primer binding sites and extend into a predator specific sequence. They help prevent predator DNA amplification but simultaneously enable amplification of DNA from prey items. We designed blocking primers for *N. savayensis*, *M. berndti* and *S. microstoma* to minimize prey DNA blocking (see guidelines in [43]):

5’-CAAAGAATCAGAATAGGTGTTGGTAAAGA-3’,

5’-CAAAGAATCAGAACAGGTGTTGATAAAGG-3’

and 5’-CAAAGAATCAGAATAGGTGTTGATAAAGA-3 respectively.

Primers were modified at the 3’end with a Spacer C3 CPG (3 hydrocarbons) to prevent elongation without affecting their annealing properties [41].

#### Sample multiplexing and library preparation for Roche 454 FLX sequencing

We used a hierarchical tagging approach with a combination of tailed PCR primers and 454 Multiplex Identifiers (MIDs) to sequence all samples in a single 454 run. Five pairs of the versatile primers, mlCOIintF and jgHCO2198, were synthetized each with 6 base pair tags at their 5’end (T1: AGCACG, T2: ACGCAG, T3: ACTATC, T4: AGACGC, T5: ATCGAC). We tested these tailed primer pairs (e.g. P1: T1-mlCOIintF × jgHCO2198-T1, P2: T2-mlCOIintF × jgHCO2198-T2) across templates from a diversity of phyla and found that they did not affect PCR performance (data not presented). Primer sets P1, P2, P3, P4 were used to amplify three gut content samples each and P5 was used for four samples. Five PCR replications and a negative control (no DNA template) were generated per sample to account for PCR drift [44] and to check for PCR contaminants. PCR products of the five replicates were pooled, run on 1.5% agarose gels, and the fragment excised to ensure that all primer dimer was screened away. PCR amplicons were purified using QIAGEN® MiniElute columns, eluted in 12 μl elution Buffer, and PCR product concentration measured using the Qubit® Fluoremeter (Invitrogen). Equimolar amounts of each sample were combined in three tubes, each tube containing amplicons generated with each of the five tailed primer pairs. We prepared these three mixes with the NEBNext Quick DNA Sample Prep Reagent Set 2 (New England BioLabs), which includes end-repair and dA-tailing chemistry and then ligated with MIDs (9, 10 and 11) using the FLX Titanium Rapid Library MID Adaptors Kit (Roche). Ligated PCR products were purified using Agencourt AMPure (Beckman Coulter Genomics), eluted in 40 μl of TE buffer, and pooled prior to emulsion PCR and 454 sequencing. Note that these 16 gut content samples were combined with 44 other samples in this run (multiplexed using the five tailed PCR primers and 12 MIDs).

### Analysis of sequence data

**Figure 2:**
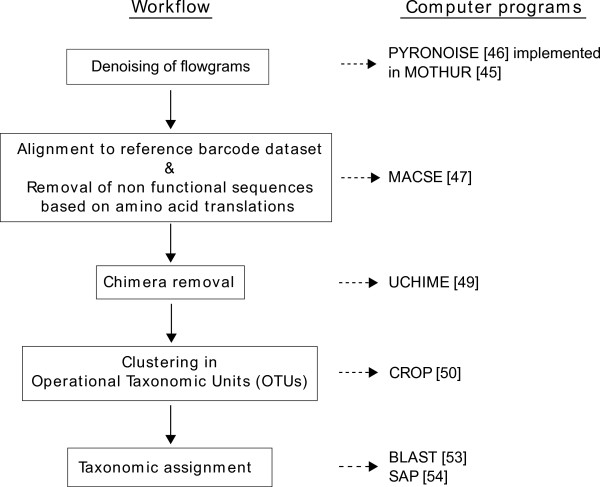
Schematic representation of the bioinformatics pipeline used for analysis of COI sequence 454 dataset.

A diagram of the bioinformatics pipeline is provided in Figure 2.

#### Denoising

“Standard flowgram file” (.sff) is the standard output of 454 platforms. It contains bases, quality and strength of the signal for each read. We used the program Mothur [45] to extract the flowgram data (.flow file) and sort reads as follow: 1) we partitioned flowgrams per sample based on barcodes and MIDs, 2) we discarded reads with more than two mismatches in the primer sequence, 3) we discarded reads with less than 200 flows (including primers and barcode), 4) we discarded or trimmed flowgrams based on standard thresholds for signal intensity (as suggested in [46]). Following this initial quality filtering, we conducted additional denoising of flowgrams using a mothur implementation of Pyronoise [46] that uses an expectation-maximization algorithm to adjust flowgrams and translates them to DNA sequences (command shhh.flows).

#### Alignment to reference barcode database and removal of non-functional sequences

We used amino acid translations to align sequences to the BIOCODE reference dataset using MACSE v1.00 [47]. Quality-filtered sequences were sequentially aligned and added to the reference dataset using the option “enrichAlignment”. This alignment strategy is only reasonable because the studied COI fragment is highly conserved at the amino acid levels. To further optimize computing time, sequences were split into subsets containing 500 sequences that were aligned in parallel thanks to a computer farm and then progressively merged into a single final alignment using the option “alignTwoProfiles”. MACSE can detect and quantify interruptions in open reading frames due to: (1) nucleotide substitutions that result in stop codons and (2) insertion or deletion of nucleotides (non multiples of three) that induce frameshifts. Sequences with stop codons are likely bacterial sequences, pseudogenes or chimeric sequences. On the other hand, frameshifts may be caused by sequencing errors that are frequent with the 454 platform [48]. MACSE can also detect and quantify insertions and deletions that do not lead to interruptions in open reading frames. COI is relatively conserved and indels are relatively uncommon. For example, only 0.9% of the sequences in BIOCODE dataset (including platyhelminthes, gastropods and isopods) display a deletion of one codon in their COI sequence, and none of the sequences in the BIOCODE dataset have codon insertions. As a result, we decided to keep all sequences from the 454 dataset which satisfied the following criteria: no stop codons, no frame shifts, no insertions and less than four deletions. For the final dataset we retained all sequences with a single frameshift when they had no stop codon, no insertions and no deletions to account for sequencing errors. Alignment of these sequences with frameshift required insertion or deletion of a nucleotide either at the first, second or third codon position. However, because the correct position could not be known, we chose to remove these codons all together.

#### Chimera removal

We used the BIOCODE reference dataset to facilitate chimera detection implemented in UCHIME [49].

#### Clustering sequences in operational taxonomic units (OTUs)

Our dataset comprised sequences belonging to a diversity of taxonomic groups that are known to have dissimilar rates of COI evolution. This means that using a fixed sequence dissimilarity cutoff (i.e. 5%) for clustering OTUs may not result in accurate species delineations. Therefore, rather than using a conventional hierarchical clustering method, we ran CROP [50], a Bayesian clustering program that delineates OTUs based on the natural distribution of the data. The program uses user-defined lower and upper bound variance to generate clusters with different standard deviations. The settings used in CROP for clustering sequences in OTUs will determine our estimation of taxonomic diversity in each sample. Ideally, each OTU should represent an evolutionary distinct unit. In order to optimize lower and upper bound values, we first use CROP to cluster sequences from the reference BIOCODE database using a variety of thresholds that in turn correspond to sequence dissimilarities (e.g., lower and upper values of 3 and 4 correspond to sequence dissimilarities of between 6% and 8% respectively). The following paired lower and upper thresholds were tested because they are within the range of intra- and inter-specific sequence dissimilarity reported in the literature for marine invertebrates [2,43,51,52]: -l 1.5 -u 2.5; -l 2.5 -u 3.5; -l 3.0 -u 3.75; - 3.0 -u 4.0; -l 3.25 -u 4.25. We particularly examined the frequency of false positives (splitting of a taxon in two or more clusters because of deep intraspecific variation) and false negatives (lumping of two or more sister taxa together because of shallow interspecific divergence) in comprehensively sampled and diverse groups (i.e. Scaridae, Trapeziidae, Cypraeidae) and found that priors of –l 3.0 and –u 4.0 provided the best results (data not shown). Yet, because the algorithm is based on stochastic processes, CROP can still find clusters with dissimilarities as low as 4-5% or as high as 9-10% as long as there are enough sequences supporting the existence of such clusters (Hao X. pers. comm.).

#### Taxonomic assignment of OTUs

We performed BLAST searches [53] of representative sequences in the local BIOCODE database (implemented in Geneious) and in GENBANK. We considered species level match when sequence similarity was at least 98% [2,51,52]. Whenever sequence similarity was lower than 98%, we used the Bayesian approach implemented in the Statistical Assignment Package (SAP, [54]) to assign the sequence to a higher taxonomic group. SAP retrieves GENBANK homologues for each query sequences and builds 10,000 unrooted phylogenetic trees. It then calculates the posterior probability for the query sequence to belong to a taxonomic group. Here we allowed SAP to download 50 GENBANK homologues at ≥70% sequence identity and we accepted assignments at a significance level of 95% (posterior probability). We combined taxonomic information and number of sequences per OTU and per sample into a summary table for downstream analysis.

## Results

### Primer design and performance

**Figure 3:**
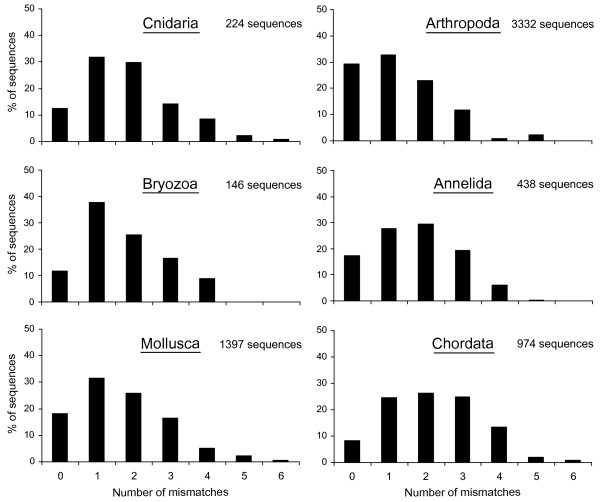
Distribution of mismatches between the “mlCOIint” primer sequence and templates from the Moorea BIOCODE database.

We were able to find a relatively well-conserved priming site from an alignment of COI barcode sequences provided by the Moorea BIOCODE project. The degenerate forward mlCOIintF and mlCOIintR (128 fold degeneracy) were designed to be used in combination with versatile primers commonly used for DNA barcoding (Table 1) to target 313 bp and 319 bp fragment lengths respectively (Figure 1). Analysis of primer-template mismatches across the BIOCODE reference library revealed that the maximum number of mismatches between sequences of six major marine phyla and the new designed primer sequence never exceeded six (Figure 3) with the majority of sequences showing less than four mismatches (Cnidaria: 88%, Arthropoda: 97%, Bryozoa: 91%, Annelida: 94%, Mollusca: 92%, Chordata: 84%).

Preliminary tests showed that the forward mlCOIintF primer used in combination with the reverse jgHCO2198 (Table 1) amplified the highest proportion of metazoan diversity tested herein (91% - Table 2). On the other hand, the reverse mlCOIintR primer performed poorly whether it was used with LCO1490, dgLCO1490 or jgLCO1490 (57%, 60% and 64% respectively – Table 2). Despite the degenerate sites in both mlCOIintF and jgHCO2198 primer sequences, particularly at the third codon position (Table 1), there was no evidence of non-specific binding (see single bands on agarose gel pictures in Additional file 2) using the touchdown PCR thermal profile. A total of 87% (250 of 285) of templates successfully amplified, among which 93% provided good quality sequences (GENBANK accession numbers KC706674-KC706906). We observed high amplification success for Arthropoda (88%, n = 99; Table 3), Molluscs (90%, n = 52), Cnidaria (88%, n = 28), Annelida (100%, n = 25), Chordata (83%, n = 18), Echinodermata (100%, n = 11), Bryozoa (100%, n = 9) and Sipuncula (100%, n = 5). In comparison, primer sets currently used for DNA barcoding to target the 658 bp COI fragment, LCO1490 × HCO2198, dgLCO1490 × dgHCO2198, and jgLCO1490 × jgHCO2198 (Table 1) had lower amplification successes (76%, 77% and 77% of successful amplifications across all templates respectively; Table 3). The mini-barcode primer set, Uni-MinibarF1 with Uni-MinibarR1, performed very poorly across the diversity of templates (27% amplification).

**Table 2:** Preliminary tests to determine the primer combination that performed best to amplify a short COI fragment

	***Forward primer***	**mlCOIintF**			**LCO1490**	**dgLCO1490**	**jgLCO1490**
	***Reverse primer***	**HCO2198**	**dgHCO2198**	**jgHCO2198**	**mlCOIintR**		
	***Fragment length (bp)***	**313**	**313**	**313**	**319**	**319**	**319**
Phylum	Cnidaria (6)	6	6	6	2	2	2
	Arthropoda (18)	16	15	16	12	11	11
	Rotifera (1)	1	1	1	0	0	0
	Entoprocta (1)	0	0	0	1	1	0
	Annelida (4)	4	4	4	3	4	4
	Nemertea (2)	2	2	2	0	0	1
	Mollusca (9)	7	7	8	7	7	7
	Echiura (1)	1	1	1	1	1	1
	Chordata (2)	2	2	2	1	2	1
	Hemichordata (2)	2	2	2	0	0	2
	Echinodermata (1)	1	1	1	0	0	1
	**TOTAL (47)**	**42**	**41**	**43**	**27**	**28**	**30**
		**89%**	**87%**	**91%**	**57%**	**60%**	**64%**

Columns show the number of taxa for which the target region was successfully amplified. The total number of taxa used for each phylum is displayed in parentheses.

**Table 3:** Performance of universal primer sets for COI across phyla

	***Forward primer***	**“mlCOIintF”**	**“LCO1490”**	**“dgLCO1490”**	**“jgLCO1490”**	**“Uni-MinibarF”**
	***Reverse primer***	**“jgHCO2198”**	**“HCO2198”**	**“dgHCO2198”**	**“jgHCO2198”**	**“Uni-MinibarR1”**
	***Fragment length (bp)***	**313**	**658**	**658**	**658**	**130**
Phylum	Radiolaria (1)	0	0	0	0	0
	Ciliophora (1)	0	1	0	0	0
	Sarcomastigophora (1)	0	0	0	0	0
	Amoebozoa (1)	0	0	0	0	0
	Placozoa (1)	0	0	0	0	0
	Porifera (4)	4	3	3	2	2
	Cnidaria (28)	26	22	23	23	11
	Ctenophora (2)	1	0	0	0	1
	Chaetognatha (2)	2	1	2	2	0
	Nematomorpha (1)	0	0	0	0	0
	Nematoda (2)	1	1	0	0	0
	Tardigrada (1)	0	0	0	0	0
	Arthropoda (99)	87	84	80	82	30
	Platyhelminthes (4)	4	1	1	0	0
	Gastrotricha (3)	2	0	0	0	0
	Gnathostomulida (3)	2	1	0	0	0
	Rotifera (1)	1	1	1	0	0
	Entoprocta (1)	0	0	1	0	0
	Bryozoa (9)	9	9	8	7	5
	Annelida (25)	25	23	25	23	5
	Nemertea (4)	3	3	3	1	2
	Sipuncula (5)	5	5	5	5	1
	Mollusca (52)	47	45	49	48	11
	Echiura (1)	1	1	1	1	0
	Phoronida (2)	2	2	2	2	2
	Brachiopoda (1)	0	1	1	1	0
	Chordata (18)	15	9	12	12	4
	Acoelomorpha (1)	0	0	0	0	0
	Hemichordata (2)	2	0	1	2	1
	Echinodermata (11)	11	4	2	11	1
	**Total (287)**	**250**	**217**	**220**	**222**	**76**
		**(87%)**	**(76%)**	**(77%)**	**(77%)**	**(27%)**

Columns present the number of taxa for which the target region was successfully amplified. Amplification success was evaluated on agarose gels (pictures shown in Additional file 2). The total number of taxa used for each phylum is displayed in parentheses.

### Pyrosequencing of fish gut contents

We obtained a total of 93,973 flowgrams after initial denoising with Pyronoise. Alignments of sequences to the reference BIOCODE dataset using MACSE revealed 38,576 sequences (41%) with anomalies in their amino acid translation. Among them, 6407 sequences with a single frameshift but with no stop codons and no inserted or deleted codons were kept in the dataset, as we assumed they were the result of minor sequencing errors. All remaining 32,169 sequences, among which 2.4% only had a stop codon, were discarded. UCHIME detected 522 potential chimeric sequences that were also removed to obtain a final dataset of 54,875 high quality reads. The number of reads per individual varied from 1219 to 8423 (mean ± SD = 3430 ± 1104), most likely as a result of differences in ligation efficiency during addition of MIDs due to the primer tag (Additional file 3). Individual rarefaction curves implemented in R, package VEGAN [55] indicate that additional sequencing would be required for further describing the gut contents of some fishes (curves do not reach a plateau, Additional file 4).

**Figure 4:**
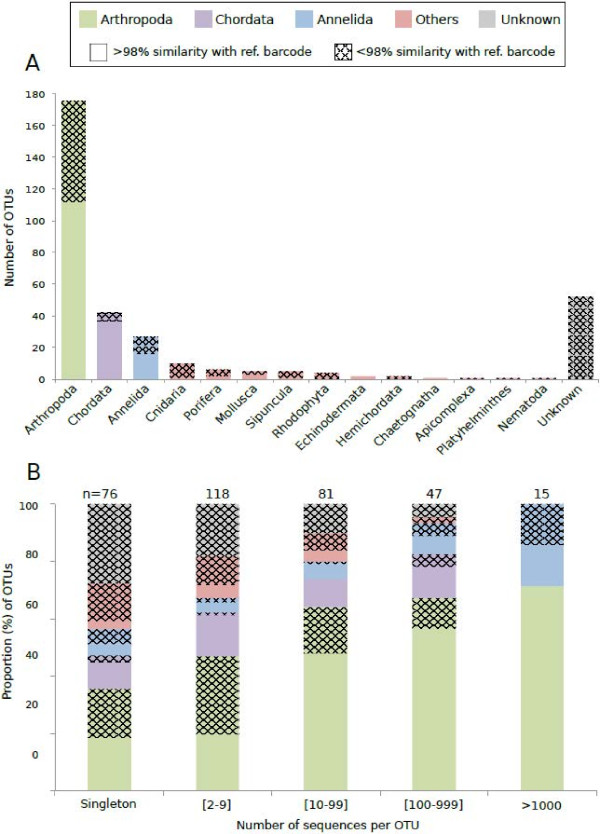
**Diversity, identity and sequence abundance of Operational Taxonomic Units (OTUs) recovered from fish gut contents. A**) The number of OTUs per phylum is presented for all fish guts pooled together. OTUs were identified from BLASTn searches performed in the Moorea BIOCODE database and GENBANK. We considered a match to be at the species level when sequence similarity to a reference barcode was >98%. When sequence similarity was < 98%, we used the Bayesian assignment tool implemented in SAP to assign each OTU to a higher taxonomic group, accepting assignments at a significance level of 95% (posterior probability). **B**) The proportion of OTUs presented per abundance classes. Abundance corresponds to the number of sequences per OTUs.

The Bayesian clustering program CROP revealed a total of 337 OTUs. None were identified as bacteria or non-COI sequences from BLAST searches. Of these, 177 OTUs (52.5%) were identified to the species level as they showed more than 98% sequence similarity with BIOCODE or GENBANK sequences (Figure 4A, Additional file 5). For the three fish species separately, 56.9%, 50.5% and 52.9% of OTUs determined from *N. savayensis*, *M. berndti* and *S. microstoma* gut contents respectively had a species-level match. Three OTUs representing the DNA of the predatory fish species themselves (*N. savayensis*: 1012 sequences, *S. microstoma*: 921 sequences; *M. berndti*: 3 sequences) were removed. Importantly, none of the 177 OTUs identified to the species level were assigned to the reference barcode of the same morphological species. Moreover, CROP was effective at discriminating closely related species, such as 12 species within the genus *Alpheus* (among which *A. obesomanus* and *A. malleodigitus* are sister species within the *obesomanus* species complex) (see Additional file 5 for more examples). The Bayesian assignment tool offered further taxonomic insights by confidently assigning 108 additional OTUs (32%) to a higher taxonomic level, and only 52 OTUs (15.4%) remained unidentified. An alignment of all representative sequences is provided in Additional file 6 and all unique sequences were deposited in the Dryad Repository doi: http://dx.doi.org/10.5061/dryad.6gd51).

OTUs belonged to 14 phyla (Figure 4A); Arthropoda, Chordata and Annelida were the most represented, with 175 OTUs (52.4%), 42 OTUs (12.6%) and 27 OTUs (8%) respectively. Species level matches were more prevalent among Chordata (88.1%) and Arthropoda (64%), two macrofaunal groups particularly well sampled by the Moorea BIOCODE teams [56] (Figure 4A). Moreover, taxonomic assignments were more prevalent for OTUs represented by a high number of sequences. For example, only 51.8% of Arthropoda OTUs matched reference barcodes when they were represented by a single sequence, whereas 100% of OTUs represented by more than 1000 sequences were assigned to BIOCODE or GenBank specimen (1: 51.8%; [2-9]: 41.8%; [10–99]: 75%; [100–999]: 83.9%; >1000: 100%; Figure 4B). Similarly, 27.6% of sequences represented by a single sequence could not be assigned to a phylum (unknown – Figure 4B) whereas none of the OTUs represented by more than 1000 sequences remained unidentified (1: 27.6%; [2-9]: 17.9%; [10–99]: 9.9%; [100–999]: 4.3%; >1000: 0%; Figure 4B).

**Figure 5:**
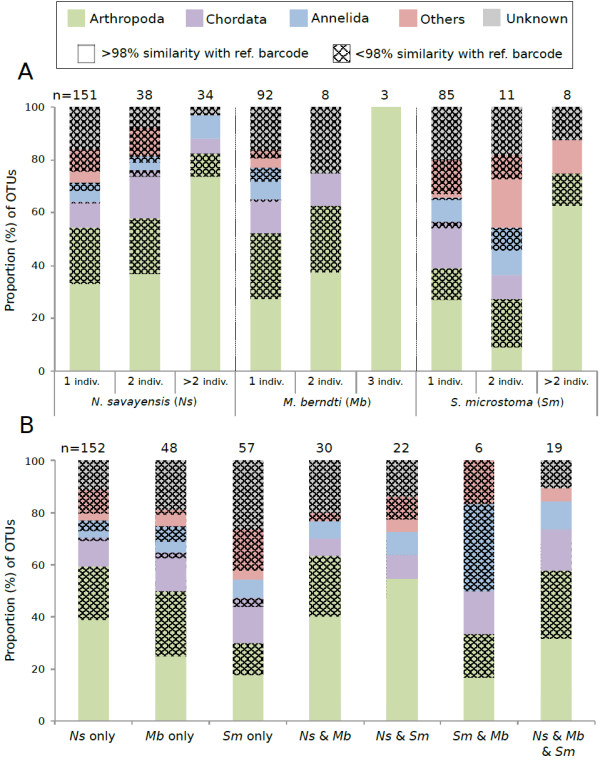
**Intra- (A) and inter-specific (B) dietary content.** The proportion of OTUs in each of the three most diverse phyla is presented. n = number of OTUs in each category. Note that the number of OTUs in A is larger than 334, the total number of OTUs found in fish gut contents, because some OTUs are shared between individuals of the three species. The list of phyla contained in the category “Others” is presented in Figure 4.

Among the 223 OTUs detected in the gut contents of *N. savayensis*, 151 (67.8%) occurred in a single individual, 38 (17%) occurred in two individuals, and 34 (15.2%) in more than two individuals (Figure 5A). Intraspecific diet overlap was lower for *M. berndti* and *S. microstoma* with only 7.8% and 10.6% of prey shared by two individuals respectively. The majority of OTUs shared by more than two individuals belonged to the phylum Arthropoda (82%, 100% and 75% for *N. savayensis*, *M. berndti* and *S. microstoma* respectively). In contrast, there was a significant overlap in dietary composition between fish species (Figure 5B): 31.8% of OTUs detected in the guts of *N. savayensis* were also detected in the guts of *M. berndti* and *S. microstoma*, and 53.4% and 45.2% of OTUs in *M. berndti* and *S. microstoma* were shared with *N. savayensis*/*S. microstoma* and *N. savayensis*/*M. berndti* respectively. OTUs shared among predatory fish were mostly Arthropoda, Chordata and Annelida, but also included Mollusca, Echinodermata, Cnidarian, Porifera and Hemichordata.

## Discussion

The high level of variability in the COI region is problematic for designing a PCR primer internal to the 658 bp COI barcoding region [8]. As shown in this study, the mini-barcode primer set [17], which represents the only published attempt at designing versatile primers for a short COI fragment to date, is not effective across taxonomic groups. We present an alternative primer set and we show how it can be used for metabarcoding analyses.

We designed the forward and reverse primers, mlCOIintF and mlCOIintR, within the 658 bp COI barcoding region using a total of seven degenerate bases to accommodate variation in the priming region. The forward internal primer was always more effective when used in combination with HCO2198 (and its degenerate versions dgHCO2198 and jgHCO2198) than its reverse complement used with LCO1490 (and its degenerate versions dgLCO1490 and jgLCO1490), which likely reflects higher incompatibilities in the LCO1490 priming site than in the HCO2198 priming site [34]. This had been previously reported for nematodes which display a three base pair deletion in the LCO1490 priming region [6]. The overall performance of mlCOIintF used with jgHCO2198 was superior to traditional barcoding primers. We demonstrate its remarkable efficacy across Arthropoda, Mollusca, Cnidaria, Annelida, Chordata, Echinodermata, Bryozoa and Sipuncula, although further tests should be conducted to evaluate its performance across less represented phyla (less than five species tested). Nevertheless, this new primer set appears to be an exceptional candidate for DNA barcoding and metabarcoding.

Higher degeneracy results in better amplification when primer-sequence mismatches are present, but a major downside can be the higher likelihood of non-specific primer annealing. Amplification tests conducted across 284 templates showed no evidence for amplification of non-target loci (single PCR band of expected size). The touchdown PCR profile may have helped increase the probability of primer-template specificity with high annealing temperatures during the first PCR cycles. Nevertheless, we also experimented with PCR conditions such as 35 cycles at 48°C for selected samples without observing any evidence for non-selective amplification (data not presented). Amplification and pyrosequencing of the 313 bp COI fragment from fish gut contents represents a better test of the likelihood of this primer set to co-amplify contaminants. Bacteria are particularly preponderant in gut and faecal samples [43] and can become problematic when misconstrued as prey items [57]. We used a sequence analysis pipeline that takes advantage of the coding properties of the COI region to exclude dubious DNA fragments. As a result, 34.2% of sequences were removed from the dataset, among which 2.4% which had a stop codon were potential bacteria. Most anomalies were not attributable to co-amplification of contaminants but rather base insertions causing frameshifts. Pyronoise (used as initial denoising) removes errors caused by incorrect interpretation of signal intensity during 454 pyrosequencing, therefore, numerous frameshifts may in fact be the result of nucleotide mis-incorporation during PCR amplification. Therefore, we highly recommend using a proofreading taq polymerase to generate amplicons with fewer errors in future metabarcoding analysis. DNA may also get damaged during digestion [14]. Other types of environmental samples where animals are collected alive (i.e. plankton tows) may be less susceptible to this type of error and should be tested.

Diversity analysis was conducted with a high-quality sequence dataset free of non-coding dubious sequences to ensure the exclusion of artefacts. A total of 344 OTUs spanning 14 different phyla were identified which further confirms the remarkable versatility of the primer set. Arthropoda, Chordata and Annelida were the most represented in terms of number of OTUs. This is in accordance with our morphological observations of prey remains, as well as with previous studies that described these three groups as the main food source of these generalist fish species [40,58-60]. Among all prey OTUs, 52.5% had a direct match with a reference barcode, mostly from the Moorea BIOCODE sequence library. Although remarkable, the proportion of species-level assignments is lower than in a previous dietary study conducted in Moorea, where 94% of undigested prey found in the guts of common generalist predatory fish could be identified using DNA barcoding of individual prey items [56]. The metabarcoding analysis conducted in the present study is not restricted to large prey (>2 mm) with hard parts, such as decapods and molluscs that received in-depth sampling by the Moorea BIOCODE teams [56]. Most unassigned OTUs belong to under-represented phyla (i.e. Porifera, Sipuncula and Rhodophyta), possibly pelagic (i.e. Maxillipoda) or small sized species (< 2 mm adult size). Interestingly, we found that OTUs represented by fewer sequences or OTUs detected in the guts of a single fish were more likely to remain unidentified. It is well known that primer bias (the number and position of mismatches with the primer sequence) and biological factors (i.e. level of digestion [61], variation in the amount of DNA target between tissue types and genome size [62], or differences in DNA survival rates during digestion [63]) affect quantitative estimates. Yet, assuming that BIOCODE was able to inventory and barcode most common fish and macro-invertebrates of the Moorea ecosystem, this suggests that species represented by a single sequence are either mostly rare or belong to small sized organisms. Due to the relatively lower sampling effort dedicated to the pelagic environment relative to the benthic environment by BIOCODE, we expected the frequency of species assignments to be lower for the strictly planktivorous species *N. savayensis* than for the strictly benthic feeder *S. microstoma*. However, our analysis revealed that *N. savayensis* had consumed eggs or larvae of numerous benthic species, whilst *S. microstoma* appeared to be very effective at sampling juvenile and adult stages of coral reef associated fish and motile invertebrates. We found 55 OTUs in the guts of *S. microstoma*, among which were ten arthropods and two fish OTUs that were never collected during the 6 years of the BIOCODE project. This shows that fish are great integrators of their immediate environment as they consume species that are not easily accessible to traditional sampling methods (see [36]). Metabarcoding analysis also detected unexpected species, including the terrestrial crab *Cardinosa carniflex* (which has planktonic larvae) and the crown-of-thorn seastar *Acanthaster planci* (a voracious predator responsible for dramatic reductions in coral cover and changes in benthic communities in Moorea between 2009 and 2011 [64,65]).

All adult fish were collected on the same night at the same site within a short period of time, enabling some preliminary insights on food partitioning among coral reef fishes. The extent of dietary overlap for species coexisting on coral reefs has long been debated [66], but overlap has often been estimated using dietary data with low taxonomic resolution [67]. We found limited evidence for dietary partitioning between species despite different feeding modes while intra-specific overlap in prey composition was more limited. Such intraspecific partitioning may be due to intraspecific competition or individual specialization, with all three species having access to a large pool of shared prey [68]. We also observed large variation in prey diversity between individual fish that could either be caused by differences in feeding intensity or efficacy. Together these preliminary results further highlight the importance of using high-resolution dietary information and consider individual level variation in prey consumption for understanding the role of food partitioning for the coexistence of coral reef fishes.

## Conclusions

The molecular analysis of gut contents targeting the 313 COI fragment using the newly designed mlCOIintF primer in combination with the jgHCO2198 primer offers enormous promise for metazoan metabarcoding studies. This primer set performs exceptionally well across metazoan phylogenetic diversity. We believe that this primer set will be a valuable asset for a range of applications from large-scale biodiversity assessments to food web studies. In particular, it could be used to rapidly assess anthropogenic impacts on biodiversity and ecosystem function, especially in highly diverse and fragile environments such as coral reefs or tropical forests.

## Competing interests

The authors declare that they have no competing interests.

## Author’ contributions

ML, SCM, CPM, JTB and RJM designed the study. ML designed the versatile primers and blocking primers, collected the fish, performed the laboratory work for metabarcoding analysis of gut contents, performed data analysis and wrote the manuscript. CM provided the Moorea BIOCODE sequence library and genomic DNA samples. NA conducted primers tests in the laboratory. JTB helped collect the fish and tested the blocking primers in the laboratory. RJM helped computing primer-template mismatches. JYY and VR provided critical help for analysing 454 sequence data. SCM supervised the project and helped writing the manuscript. All authors read and approved the final manuscript.

## Supplementary Material

Additional file 1 **List of taxa used for comparing the performance of primer sets.** Genomic DNA for both terrestrial and marine species was provided by the Moorea Biocode project. Photographs and additional information about each specimen can be obtained at http://biocode.berkeley.edu. Click here for file

Additional file 2 **Agarose gel image showing the amplification success of a 313 bp COI fragment across taxa belonging to 30 animal phyla.** The forward primer mlCOIintF and reverse primer jgHCO2198 were used. List of taxa is shown in Additional file 1. Summary of results is shown in Table 3 of the main text. Click here for file

Additional file 3 **Differences in sequence recovery due to a bias in ligation efficiency during addition of multiplex identifiers (MIDs).** We used a hierarchical tagging approach: following PCR amplification with versatile primers synthetized with a 6 bp barcode (T1 through T5) at the 5’ end, samples were pooled resulting in 12 pools of five samples each. A different MID identifier was ligated to each pool. The mean (± SD) proportion of sequences per sample is represented on the y axis. Twelve MID tags were used to multiplex 60 samples in this 454 sequencing run. Click here for file

Additional file 4 **Individual rarefaction curves illustrating the accumulation of prey diversity with sequencing.** Each curve represents the gut contents of an individual fish. Click here for file

Additional file 5 **List of taxa recovered from fish gut contents by targeting the 313 bp COI region.** A representative sequence per OTU was used for taxonomic identification. BIOCODE reference specimen number or GENBANK accession number are indicated when sequence similarity with reference barcode sequence was >98% (using BLASTn search). Photographs and additional information about BIOCODE reference specimens can be obtained at http://biocode.berkeley.edu. When sequence similarity was < 98%, we used the Bayesian assignment tool implemented in SAP to assign each OTU to a higher taxonomic group. # indiv.: number of individual fish. # seq.: number of sequences for each OTU. Click here for file

Additional file 6 **Fasta formatted alignment of OTU representative sequences.** See Additional file 5 for taxonomic identification. Click here for file
