# Supplementary material for: A new versatile primer set targeting a short fragment of the mitochondrial COI region for metabarcoding metazoan diversity: application for characterizing coral reef fish gut contents
Source: Front Zool. 2013 Jun 14;10:34. doi: 10.1186/1742-9994-10-34 (PMC3686579; doi:10.1186/1742-9994-10-34)
Supplement: Additional file 1 — List of taxa used for comparing the performance of primer sets. Genomic DNA for both terrestrial and marine species was provided by the Moorea Biocode project. Photographs and additional information about each specimen can be obtained at http://biocode.berkeley.edu. [file 1742-9994-10-34-S1.docx]

List of taxa used for comparing the performance of primer sets. Genomic DNA for both terrestrial and marine species was provided by the Moorea Biocode project. Photographs and additional information about each specimen can be obtained at http://biocode.berkeley.edu

| **Plate** | **Well** | **Extraction ID** | **Phylum** | **Lowest Taxon** |
| --- | --- | --- | --- | --- |
| P1 | A01 | MBIO32019.1.3 | Radiolaria | Radiolaria |
|  | A02 | MBIO32326.1.3 | Ciliophora | Ciliophora |
|  | A03 | MBIO27028.1.3 | Sarcomastigophora | Polycystinea |
|  | A04 | MBIO26935.1.3 | Amoebozoa | *Collozoum* |
|  | A05 | MBIO41267.1.2 | Placozoa | Placozoa |
|  | A06 | MBIO19746.1.3 | Porifera | *Astrosclera* |
|  | A07 | MBIO28690.1.3 |  | *Dysidea* |
|  | A08 | MBIO19755.1.3 |  | *Corticium* |
|  | A09 | MBIO26763.1.3 |  | Verongida |
|  | A10 | MBIO28985.1.3 | Cnidaria | *Boloceroides mcmurrichi* |
|  | A11 | MBIO28651.1.3 |  | *Triactis producta* |
|  | A12 | MBIO19735.1.3 |  | *Sinularia* |
|  | B01 | MBIO29732.1.3 |  | Cerianthidae |
|  | B02 | MBIO21262.1.3 |  | *Pocillopora woodjonesi* |
|  | B03 | MBIO20602.1.3 |  | *Tubastraea* |
|  | B04 | MBIO26658.1.3 |  | Caryophyllidae |
|  | B05 | MBIO32470.1.3 |  | *Montastraea curta* |
|  | B06 | MBIO29671.1.3 |  | *Acropora palmerae* |
|  | B07 | MBIO30304.1.3 |  | *Fungia concinna* |
|  | B08 | MBIO28738.1.3 |  | Cubozoa |
|  | B09 | MBIO41281.1.2 |  | Cubozoa |
|  | B10 | MBIO30406.1.3 |  | *Carybdea alata* |
|  | B11 | MBIO21597.1.3 |  | *Velella velella* |
|  | B12 | MBIO28901.1.3 |  | *Distichopora violacea* |
|  | C01 | MBIO32446.1.3 |  | *Eudendrium* |
|  | C02 | MBIO28527.1.3 |  | *Cladonema* |
|  | C03 | MBIO28748.1.3 |  | *Millepora platyphylla* |
|  | C04 | MBIO26776.1.3 |  | *Plumularia strictocarpa* |
|  | C05 | MBIO26769.1.3 |  | *Lytocarpia phyteuma* |
|  | C06 | MBIO32235.1.3 |  | *Nemalecium lighti* |
|  | C07 | MBIO32440.1.3 |  | *Halopteris polymorpha* |
|  | C08 | MBIO28344.1.3 |  | *Halecium* |
|  | C09 | MBIO26783.1.3 |  | Siphonophora |
|  | C10 | MBIO28446.1.3 |  | Abylidae |
|  | C11 | MBIO32021.1.3 |  | *Forskalia* |
|  | C12 | MBIO32011.1.3 |  | *Linuche* |
|  | D01 | MBIO27183.1.3 |  | *Pelagia* |
|  | D02 | MBIO27029.1.3 | Ctenophora | *Velamen* |
|  | D03 | MBIO26933.1.3 |  | *Pleurobrachia* |
|  | D04 | MBIO19265.1.3 | Chaetognatha | Chaetognatha |
|  | D05 | MBIO26497.1.3 |  | *Sagitta* |
|  | D06 | MBIO40517.1.3 | Nematomorpha | Nematomorpha |
|  | D07 | MBIO28565.1.3 | Nematoda | Nematoda |
|  | D08 | MBIO28704.1.3 |  | Nematoda |
|  | D09 | MBIO28273.1.3 | Tardigrada | Tardigrada |
|  | D10 | MBIO29358.1.3 | Arthropoda | Notodelphidae |
|  | D11 | MBIO40739.1.3 |  | *Neonaesa rugosa* |
|  | D12 | MBIO40583.1.3 |  | Tanaidacea |
|  | E01 | MBIO14605.1.3 |  | Calinoida |
|  | E02 | MBIO35283.1.2 |  | Mysidea |
|  | E03 | MBIO43028.1.2 |  | *Geogarypus longidigitatus* |
|  | E04 | MBIO43026.1.2 |  | *Tangaroa tahitiensis* |
|  | E05 | MBIO22382.1.4 |  | Bruchinae |
|  | E06 | MBIO22410.1.4 |  | Cerambycidae |
|  | E07 | MBIO35870.1.4 |  | Coleoptera |
|  | E08 | MBIO22411.1.4 |  | Tephritidae |
|  | E09 | MBIO22413.1.4 |  | Tipulidae |
|  | E10 | MBIO22663.1.3 |  | *Ornidia obesa* |
|  | E11 | MBIO35851.1.4 |  | Diptera |
|  | E12 | MBIO35882.1.4 |  | Hemiptera |
|  | F01 | MBIO22406.1.4 |  | *Macroglossum hirundo* |
|  | F02 | MBIO22409.1.4 |  | *Assara halmophila* |
|  | F03 | MBIO22434.1.4 |  | Geometridae |
|  | F04 | MBIO22600.1.3 |  | Noctuidae |
|  | F05 | MBIO22618.1.3 |  | Tineidae |
|  | F06 | MBIO37696.1.3 |  | *Cydia pseudomalesana* |
|  | F07 | MBIO37414.1.4 |  | *Tanaoctena* |
|  | F08 | MBIO37629.1.4 |  | *Cnaphalocrocis* |
|  | F09 | MBIO37715.1.4 |  | Pterophoridae |
|  | F10 | MBIO37719.1.4 |  | *Alucita* |
|  | F11 | MBIO37704.1.4 |  | *Macarostola pontificalis* |
|  | F12 | MBIO37702.1.4 |  | *Imma* |
|  | G01 | MBIO22235.1.4 |  | Mantidae |
|  | G02 | MBIO22383.1.4 |  | Chrysopidae |
|  | G03 | MBIO22302.1.5 |  | Gryllidae |
|  | G04 | MBIO22585.1.3 |  | Orthoptera |
|  | G05 | MBIO29400.1.3 |  | *Gammaropsis* |
|  | G06 | MBIO29425.1.3 |  | *Ampithoe* |
|  | G07 | MBIO29298.1.3 |  | *Polynesoecetes kekeae* |
|  | G08 | MBIO29392.1.3 |  | *Erichthonius* |
|  | G09 | MBIO30565.1.3 |  | *Globosolembos ovatus* |
|  | G10 | MBIO30699.1.3 |  | *Maera lindsae* |
|  | G11 | MBIO30560.1.3 |  | *Iphimedia* |
|  | G12 | MBIO30074.1.3 |  | *Synopia ultramarina* |
|  | H01 | MBIO30100.1.3 |  | *Paranamixis fijiensis* |
|  | H02 | MBIO29561.1.3 |  | Hyperiidae |
|  | H03 | MBIO30057.1.3 |  | *Hyale* |
|  | H04 | MBIO20162.1.3 |  | *Macrophthalmus convexus* |
|  | H05 | MBIO21744.1.3 |  | *Lucifer* |
|  | H06 | MBIO20125.1.3 |  | *Alpheus paracrinitus* |
|  | H07 | MBIO20072.1.3 |  | *Pilumnus* |
|  | H08 | MBIO21707.1.3 |  | *Dardanus lagopodes* |
|  | H09 | MBIO21730.1.3 |  | *Domecia hispida* |
|  | H10 | MBIO20396.1.3 |  | *Daldorfia horrida* |
|  | H11 | MBIO19580.1.3 |  | *Huenia* |
|  | H12 | MBIO21714.1.3 |  | *Schizophrys aspera* |
| P2 | A01 | MBIO21709.1.3 | Arthropoda | *Hirsutodynomene spinosa* |
|  | A02 | MBIO19603.1.3 |  | *Pagurixus nomurai* |
|  | A03 | MBIO19566.1.3 |  | *Pseudograpsus albus* |
|  | A04 | MBIO20096.1.3 |  | *Microprosthema* |
|  | A05 | MBIO20221.1.3 |  | *Gnathophyllum americanum* |
|  | A06 | MBIO20692.1.3 |  | *Nucia speciosa* |
|  | A07 | MBIO20579.1.3 |  | *Saron marmoratus* |
|  | A08 | MBIO20583.1.3 |  | *Anchistus demani* |
|  | A09 | MBIO20945.1.3 |  | *Etisus splendidus* |
|  | A10 | MBIO21052.1.3 |  | Callianassidae |
|  | A11 | MBIO26335.1.3 |  | *Liocarpilodes integerrimus* |
|  | A12 | MBIO26428.1.3 |  | *Geograpsus grayi* |
|  | B01 | MBIO26484.1.3 |  | *Cardisoma carnifex* |
|  | B02 | MBIO26485.1.3 |  | *Metasesarma obesum* |
|  | B03 | MBIO26486.1.3 |  | *Ocypode ceratophthalma* |
|  | B04 | MBIO26552.1.3 |  | *Axiopsis serratifrons* |
|  | B05 | MBIO28553.1.3 |  | *Portunus* |
|  | B06 | MBIO28948.1.3 |  | *Nikoides maldivensis* |
|  | B07 | MBIO29754.1.3 |  | *Tetralia cinctipes* |
|  | B08 | MBIO29663.1.3 |  | *Hymenocera picta* |
|  | B09 | MBIO30640.1.3 |  | Palicidae |
|  | B10 | MBIO30658.1.3 |  | *Sympagurus poupini* |
|  | B11 | MBIO30746.1.3 |  | *Hapalocarcinus* |
|  | B12 | MBIO30376.1.3 |  | *Petrolisthes* |
|  | C01 | MBIO30478.1.3 |  | *Atyoida pilipes* |
|  | C02 | MBIO30552.1.3 |  | *Parribacus holthuisi* |
|  | C03 | MBIO30555.1.3 |  | *Carpilius convexus* |
|  | C04 | MBIO31135.1.2 |  | *Galathea* |
|  | C05 | MBIO31382.1.2 |  | *Trapezia serenei* |
|  | C06 | MBIO31314.1.2 |  | *Thor* |
|  | C07 | MBIO19354.1.3 |  | Bopyridae |
|  | C08 | MBIO29994.1.2 |  | Aegidae |
|  | C09 | MBIO29704.1.3 |  | Munnidae |
|  | C10 | MBIO29651.1.3 |  | Gnathiidae |
|  | C11 | MBIO20191.1.3 |  | *Gonodactylellus espinosus* |
|  | C12 | MBIO20762.1.3 |  | Lysiosquillidae |
|  | D01 | MBIO26496.1.3 |  | *Pullosquilla* |
|  | D02 | MBIO30673.1.3 |  | *Leptochelia* |
|  | D03 | MBIO30686.1.3 |  | Tanaidacea |
|  | D04 | MBIO14902.1.3 |  | Labidocera |
|  | D05 | MBIO42269.1.3 |  | Harpacticoida |
|  | D06 | MBIO28958.1.3 |  | *Lepas anatifera* |
|  | D07 | MBIO21504.1.3 |  | Poecilostomatoida |
|  | D08 | MBIO27053.1.3 |  | Pyrgomatidae |
|  | D09 | MBIO22149.1.3 |  | Ostracoda |
|  | D10 | MBIO29706.1.3 |  | Pycnogonida |
|  | D11 | MBIO30473.1.3 |  | Pycnogonida |
|  | D12 | MBIO28840.1.3 | Platyhelminthes | Acerola |
|  | E01 | MBIO26514.1.3 |  | Polycladida |
|  | E02 | MBIO28569.1.3 |  | *Cheliplana* |
|  | E03 | MBIO28372.1.3 |  | Otoplanidae |
|  | E04 | MBIO28280.1.3 | Gastrotricha | *Dactylopodola agadasys* |
|  | E05 | MBIO28272.1.3 |  | *Mesodasys* |
|  | E06 | MBIO28841.1.3 |  | *Urodasys* |
|  | E07 | MBIO28274.1.3 | Gnathostomulida | Gnathostomulida |
|  | E08 | placeHolder.2115 |  | Gnathostomulida |
|  | E09 | placeHolder.2116 |  | Gnathostomulida |
|  | E10 | MBIO28292.1.3 | Rotifera | Rotifera |
|  | E11 | MBIO25473.1.3 | Entoprocta | Entoprocta |
|  | E12 | MBIO27190.1.3 | Bryozoa | *Celleporaria* |
|  | F01 | MBIO27088.1.3 |  | *Conopeum* |
|  | F02 | MBIO27194.1.3 |  | *Calyptotheca* |
|  | F03 | MBIO27085.1.3 |  | *Rhynchozoon* |
|  | F04 | MBIO27094.2.3 |  | *Figularia* |
|  | F05 | MBIO27374.1.3 |  | *Bugula dentata* |
|  | F06 | MBIO27419.1.3 |  | *Parasmittina* |
|  | F07 | MBIO27412.1.3 |  | *Stylopoma* |
|  | F08 | MBIO26965.1.3 |  | *Disporella* |
|  | F09 | MBIO40213.1.3 | Annelida | Sigallonidae |
|  | F10 | MBIO40373.1.3 |  | Echiuridae |
|  | F11 | MBIO40588.1.3 |  | Euphrosiniidae |
|  | F12 | MBIO40562.1.3 |  | Nereidae |
|  | G01 | MBIO40552.1.3 |  | *Palolo viridis* |
|  | G02 | MBIO26415.1.3 |  | Oligochaeta |
|  | G03 | MBIO21991.1.3 |  | Polychaeta |
|  | G04 | MBIO28560.1.3 |  | Arenicolidae |
|  | G05 | MBIO32346.1.3 |  | *Protodrilus* |
|  | G06 | MBIO40409.1.3 |  | *Spinther* |
|  | G07 | MBIO41618.1.3 |  | Opheliidae |
|  | G08 | MBIO41599.1.3 |  | *Saccocirrus* |
|  | G09 | MBIO40428.1.3 |  | *Pherecardia striata* |
|  | G10 | MBIO40338.1.3 |  | Dorvilleidae |
|  | G11 | MBIO29530.1.3 |  | Nereididae |
|  | G12 | MBIO40212.1.3 |  | Phyllodocidae |
|  | H01 | MBIO40387.1.3 |  | Syllidae |
|  | H02 | MBIO40395.1.3 |  | Hesionidae |
|  | H03 | MBIO40379.1.3 |  | *Gastrolepidia clavigera* |
|  | H04 | MBIO40540.1.3 |  | Glyceridae |
|  | H05 | MBIO40551.1.3 |  | Syllidae |
|  | H06 | MBIO42298.1.3 |  | Chrysopetalidae |
|  | H07 | MBIO40333.1.3 |  | *Chaetopterus* |
|  | H08 | MBIO40578.1.3 |  | Spionidae |
|  | H09 | MBIO40353.1.3 |  | Terebellidae |
|  | H10 | MBIO27923.1.3 | Nemertea | Nemertea |
|  | H11 | MBIO27930.1.3 |  | *Cephalothrix* |
|  | H12 | MBIO32514.2.3 |  | *Baseodiscus delineatus* |
| P3 | A01 | MBIO28140.1.3 |  | Heteronemertea |
|  | A02 | MBIO21561.1.3 | Sipuncula | Sipuncula |
|  | A03 | MBIO19290.1.3 |  | Sipuncula |
|  | A04 | MBIO30021.1.2 |  | Sipuncula |
|  | A05 | MBIO28885.1.3 |  | Sipuncula |
|  | A06 | MBIO21438.1.3 |  | *Phascolosoma* |
|  | A07 | MBIO40201.1.3 | Mollusca | *Neritina turrita* |
|  | A08 | MBIO40082.1.3 |  | *Phenecolepas* |
|  | A09 | MBIO40494.1.3 |  | Succineidae |
|  | A10 | MBIO41198.1.3 |  | Charopidae |
|  | A11 | MBIO14396.1.3 |  | Arcoidea |
|  | A12 | MBIO28020.1.3 |  | *Ctena bella* |
|  | B01 | MBIO21458.1.3 |  | *Lithophaga* |
|  | B02 | MBIO26406.1.3 |  | *Spondylus* |
|  | B03 | MBIO26469.1.3 |  | *Fragum fragum* |
|  | B04 | MBIO26582.1.3 |  | *Tellina crucigera* |
|  | B05 | MBIO28617.1.3 |  | *Gafrarium pectinatum* |
|  | B06 | MBIO30296.1.3 |  | *Chama cerion* |
|  | B07 | MBIO40105.1.3 |  | Galeommatoidea |
|  | B08 | MBIO41371.1.3 |  | *Octopus* |
|  | B09 | MBIO27365.1.3 |  | *Siphonaria* |
|  | B10 | MBIO30147.1.3 |  | Pyramidella |
|  | B11 | MBIO30323.1.3 |  | *Turbo petholatus* |
|  | B12 | MBIO21639.1.3 |  | Dialidae |
|  | C01 | MBIO27297.1.3 |  | Cerithiopsidae |
|  | C02 | MBIO27321.1.3 |  | *Triphora* |
|  | C03 | MBIO26585.1.3 |  | *Cerithium* |
|  | C04 | MBIO30498.1.3 |  | *Melanella* |
|  | C05 | MBIO26481.1.3 |  | *Aplysia parvula* |
|  | C06 | MBIO29853.1.3 |  | *Haminoea* |
|  | C07 | MBIO19940.1.3 |  | *Cymatium nicobaricum* |
|  | C08 | MBIO21775.1.3 |  | *Strombus gibberulus* |
|  | C09 | MBIO27024.1.3 |  | *Atlanta* |
|  | C10 | MBIO29213.1.3 |  | Caecidae |
|  | C11 | MBIO27366.1.3 |  | *Serpulorbis* |
|  | C12 | MBIO29263.1.2 |  | *Cypraea punctata* |
|  | D01 | MBIO29259.1.3 |  | *Rissoina ambigua* |
|  | D02 | MBIO21401.1.3 |  | *Mitra assimilis* |
|  | D03 | MBIO21410.1.3 |  | *Oliva annulata* |
|  | D04 | MBIO19474.1.3 |  | *Terebra affinis* |
|  | D05 | MBIO19492.1.3 |  | *Mitrella margarita* |
|  | D06 | MBIO19397.1.3 |  | *Drupa clathrata* |
|  | D07 | MBIO26655.1.3 |  | *Vexillum cancellarioides* |
|  | D08 | MBIO30979.1.3 |  | *Conus nanus* |
|  | D09 | MBIO26308.1.3 |  | *Peristernia* |
|  | D10 | MBIO26380.1.3 |  | *Prodotia iostoma* |
|  | D11 | MBIO30148.1.3 |  | *Nassarius* |
|  | D12 | MBIO40309.1.3 |  | *Georissa* |
|  | E01 | MBIO29793.1.2 |  | Aeolidiidae |
|  | E02 | MBIO28543.1.3 |  | Discodorididae |
|  | E03 | MBIO29009.1.3 |  | *Gymnodoris inornata* |
|  | E04 | MBIO29032.1.3 |  | *Polycera japonica* |
|  | E05 | MBIO40194.1.3 |  | *Plakobranchus* |
|  | E06 | MBIO41241.1.3 |  | *Diastole conica* |
|  | E07 | MBIO21622.1.3 |  | *Limacina* |
|  | E08 | MBIO21734.1.3 |  | Thecosomata |
|  | E09 | MBIO27047.1.3 |  | *Creseis* |
|  | E10 | MBIO32399.1.3 |  | Polyplacophora |
|  | E11 | MBIO19324.1.3 | Echiura | Echiuridae |
|  | E12 | MBIO30050.1.3 | Phoronida | *Phoronis* |
|  | F01 | MBIO30041.1.3 |  | *Phoronis* |
|  | F02 | MBIO40148.1.3 | Brachiopoda | Brachiopoda |
|  | F03 | MBIO38570.1.3 | Chordata | *Sargocentron microstoma* |
|  | F04 | MBIO38732.1.3 |  | *Trimma milta* |
|  | F05 | MBIO38535.1.3 |  | *Thalassoma amblycephalum* |
|  | F06 | MBIO38543.1.3 |  | *Cirrhitus pinnulatus* |
|  | F07 | MBIO38548.1.3 |  | *Dascyllus flavicaudus* |
|  | F08 | MBIO38558.1.3 |  | *Chromis viridis* |
|  | F09 | MBIO38560.1.3 |  | *Caranx sexfasciatus* |
|  | F10 | MBIO38561.1.3 |  | *Myripristis violacea* |
|  | F11 | MBIO38624.1.3 |  | *Chaetodon lunulatus* |
|  | F12 | MBIO38932.1.3 |  | *Gymnothorax melatremus* |
|  | G01 | MBIO32920.1.3 |  | *Epinephelus hexagonatus* |
|  | G02 | MBIO38931.1.3 |  | *Boelkenchelys longidentata* |
|  | G03 | MBIO38937.1.3 |  | *Scorpaenodes minor* |
|  | G04 | MBIO32127.1.3 |  | *Didemnum granulatum* |
|  | G05 | MBIO32489.1.3 |  | *Ascidia archaia* |
|  | G06 | MBIO32245.1.3 |  | *Herdmania* |
|  | G07 | MBIO32022.1.3 |  | Doliolidae |
|  | G08 | MBIO27039.1.3 |  | *Salpa* |
|  | G09 | MBIO28562.1.3 | Acoelomorpha | Acoela |
|  | G10 | MBIO27430.1.3 | Hemichordata | *Ptychodera* |
|  | G11 | MBIO32533.1.3 |  | *Ptychodera flava* |
|  | G12 | MBIO14436.1.3 | Echinodermata | *Asterina* |
|  | H01 | MBIO26354.1.3 |  | *Linckia multifora* |
|  | H02 | MBIO20192.1.4 |  | *Tripneustes gratilla* |
|  | H03 | MBIO26905.1.3 |  | *Echinometra oblonga* |
|  | H04 | MBIO26844.1.3 |  | *Actinocidaris mcedwardi* |
|  | H05 | MBIO21392.1.3 |  | *Mortonia australis* |
|  | H06 | MBIO26506.1.3 |  | *Metalia* |
|  | H07 | MBIO19726.1.3 |  | *Chiridota hawaiiensis* |
|  | H08 | MBIO27919.1.3 |  | *Polyplectana* |
|  | H09 | MBIO19890.1.3 |  | *Stichopus horrens* |
|  | H10 | MBIO14445.1.3 |  | *Ophiocoma pica* |
|  | H11 |  | Negative control |  |
|  | H12 |  | Negative control |  |
|  |  |  |  |  |
