# Supplementary material for: A new versatile primer set targeting a short fragment of the mitochondrial COI region for metabarcoding metazoan diversity: application for characterizing coral reef fish gut contents
Source: Front Zool. 2013 Jun 14;10:34. doi: 10.1186/1742-9994-10-34 (PMC3686579; doi:10.1186/1742-9994-10-34)

Agarose gel image showing the amplification success of a 313bp COI fragment across taxa belonging to 30 animal phyla. The forward primer mlCOIintF and reverse primer jgHCO2198 were used. List of taxa is shown in Additional file 1. Summary of results is shown in Table 3 of the main text.


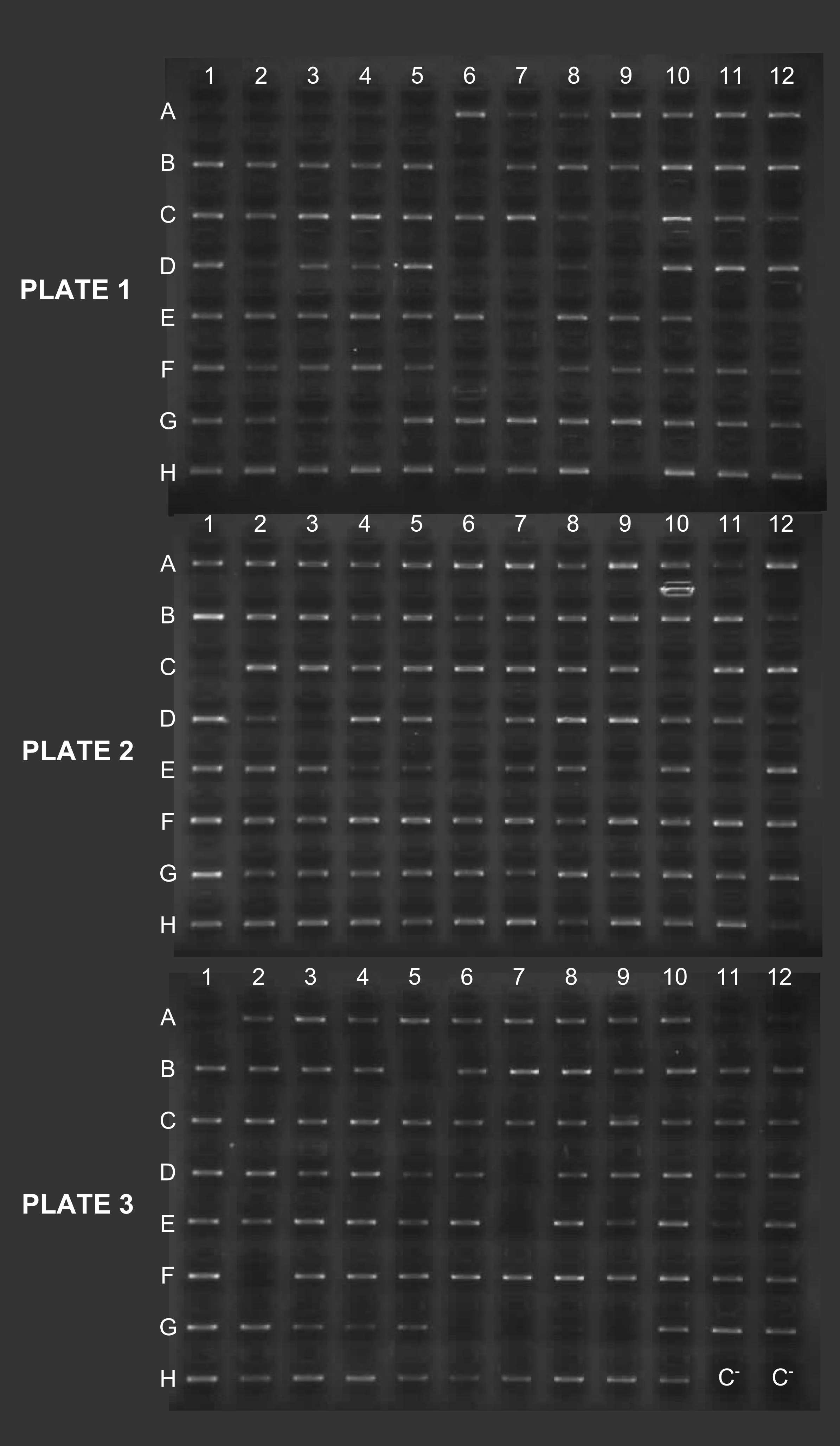

Supplement: Additional file 2 — Agarose gel image showing the amplification success of a 313 bp COI fragment across taxa belonging to 30 animal phyla. The forward primer mlCOIintF and reverse primer jgHCO2198 were used. List of taxa is shown in Additional file 1. Summary of results is shown in Table 3 of the main text. [file 1742-9994-10-34-S2.docx]
