# Supplementary material for: A new versatile primer set targeting a short fragment of the mitochondrial COI region for metabarcoding metazoan diversity: application for characterizing coral reef fish gut contents
Source: Front Zool. 2013 Jun 14;10:34. doi: 10.1186/1742-9994-10-34 (PMC3686579; doi:10.1186/1742-9994-10-34)
Supplement: Additional file 3 — Differences in sequence recovery due to a bias in ligation efficiency during addition of multiplex identifiers (MIDs). We used a hierarchical tagging approach: following PCR amplification with versatile primers synthetized with a 6 bp barcode (T1 through T5) at the 5’ end, samples were pooled resulting in 12 pools of five samples each. A different MID identifier was ligated to each pool. The mean (± SD) proportion of sequences per sample is represented on the y axis. Twelve MID tags were used to multiplex 60 samples in this 454 sequencing run. [file 1742-9994-10-34-S3.docx]

Differences in sequence recovery due to a bias in ligation efficiency during addition of multiplex identifiers (MIDs). We used a hierarchical tagging approach: following PCR amplification with versatile primers synthetized with a 6bp barcode (T1 through T5) at the 5’ end, samples were pooled resulting in 12 pools of five samples each. A different MID identifier was ligated to each pool. The mean (± SD) proportion of sequences per sample is represented on the y axis. Twelve MID tags were used to multiplex 60 samples in this 454 sequencing run.
