# Supplementary material for: A new versatile primer set targeting a short fragment of the mitochondrial COI region for metabarcoding metazoan diversity: application for characterizing coral reef fish gut contents
Source: Front Zool. 2013 Jun 14;10:34. doi: 10.1186/1742-9994-10-34 (PMC3686579; doi:10.1186/1742-9994-10-34)
Supplement: Additional file 4 — Individual rarefaction curves illustrating the accumulation of prey diversity with sequencing. Each curve represents the gut contents of an individual fish. [file 1742-9994-10-34-S4.docx]

Individual rarefaction curves illustrating the accumulation of prey diversity with sequencing. Each curve represents the gut contents of an individual fish.
