# Supplementary material for: A new versatile primer set targeting a short fragment of the mitochondrial COI region for metabarcoding metazoan diversity: application for characterizing coral reef fish gut contents
Source: Front Zool. 2013 Jun 14;10:34. doi: 10.1186/1742-9994-10-34 (PMC3686579; doi:10.1186/1742-9994-10-34)
Supplement: Additional file 5 — List of taxa recovered from fish gut contents by targeting the 313 bp COI region. A representative sequence per OTU was used for taxonomic identification. BIOCODE reference specimen number or GENBANK accession number are indicated when sequence similarity with reference barcode sequence was >98% (using BLASTn search). Photographs and additional information about BIOCODE reference specimens can be obtained at http://biocode.berkeley.edu. When sequence similarity was < 98%, we used the Bayesian assignment tool implemented in SAP to assign each OTU to a higher taxonomic group. # indiv.: number of individual fish. # seq.: number of sequences for each OTU. [file 1742-9994-10-34-S5.docx]

List of taxa recovered from fish gut contents by targeting the 313bp COI region. A representative sequence per OTU was used for taxonomic identification. BIOCODE reference specimen number or GENBANK accession number are indicated when sequence similarity with reference barcode sequence was >98% (using BLASTn search). Photographs and additional information about BIOCODE reference specimens can be obtained at http://biocode.berkeley.edu. When sequence similarity was <98%, we used the Bayesian assignment tool implemented in SAP to assign each OTU to a higher taxonomic group. # indiv.: number of individual fish. # seq.: number of sequences for each OTU.

|  | ***N. savayensis*** | | ***S. microstoma*** | | ***M. berndti*** | | ***Total*** | |  |  |  |  |
| --- | --- | --- | --- | --- | --- | --- | --- | --- | --- | --- | --- | --- |
| **OTU#** | **# seq.** | **# spec.** | **# seq.** | **# spec.** | **# seq.** | **# spec.** | **# seq.** | **# spec.** | **Phylum** | **Lowest Taxon** | **BIOCODE #** | **GENBANK #** |
| **X242** | 0 | 0 | 1 | 1 | 0 | 0 | 1 | 1 | Annelida | Palola sp. | BMOO_00579 | DQ317825 |
| **X193** | 6599 | 4 | 1 | 1 | 142 | 1 | 6742 | 6 | Annelida | Polychaeta | BMOO_07095 |  |
| **X036** | 0 | 0 | 8 | 1 | 0 | 0 | 8 | 1 | Annelida | Eunicidae | BMOO_08566 |  |
| **X035** | 3 | 2 | 0 | 0 | 1 | 1 | 4 | 3 | Annelida | Nereididae | BMOO_09151 |  |
| **X299** | 0 | 0 | 0 | 0 | 2 | 1 | 2 | 1 | Annelida | Phyllodocidae | BMOO_09153 |  |
| **X259** | 1 | 1 | 0 | 0 | 0 | 0 | 1 | 1 | Annelida | Syllidae | BMOO_10157 |  |
| **X213** | 0 | 0 | 1 | 1 | 0 | 0 | 1 | 1 | Annelida | Hesionidae | BMOO_10782 |  |
| **X023** | 1 | 1 | 568 | 1 | 0 | 0 | 569 | 2 | Annelida | Eunice | BMOO_13311 |  |
| **X154** | 809 | 1 | 0 | 0 | 0 | 0 | 809 | 1 | Annelida | Dorvilleidae | BMOO_14282 |  |
| **X125** | 7 | 1 | 319 | 2 | 1830 | 1 | 2156 | 4 | Annelida | Eunicidae | BMOO_14433 |  |
| **X037** | 0 | 0 | 0 | 0 | 17 | 1 | 17 | 1 | Annelida | Syllidae | BMOO_14486 |  |
| **X150** | 3 | 3 | 0 | 0 | 43 | 1 | 46 | 4 | Annelida | Spionidae | BMOO_14493 |  |
| **X032** | 11 | 1 | 0 | 0 | 0 | 0 | 11 | 1 | Annelida | Dorvilleidae | BMOO_14500 |  |
| **X013** | 9 | 4 | 0 | 0 | 0 | 0 | 9 | 4 | Annelida | Polychaeta | plate_M023_E9 |  |
| **X134** | 103 | 1 | 1 | 1 | 0 | 0 | 104 | 2 | Annelida | Annelida | PlateM999_F08_A6 | |
| **X016** | 0 | 0 | 78 | 1 | 0 | 0 | 78 | 1 | Annelida | Amphinomidae | XMOO_0458 |  |
| **X015** | 6 | 1 | 0 | 0 | 0 | 0 | 6 | 1 | Annelida | Phyllodocida |  |  |
| **X039** | 0 | 0 | 0 | 0 | 14 | 1 | 14 | 1 | Annelida | Eunicidae |  |  |
| **X129** | 2098 | 2 | 0 | 0 | 0 | 0 | 2098 | 2 | Annelida | Glyceridae |  |  |
| **X133** | 1156 | 1 | 0 | 0 | 0 | 0 | 1156 | 1 | Annelida | Syllidae |  |  |
| **X145** | 0 | 0 | 2 | 1 | 131 | 1 | 133 | 2 | Annelida | Eunicidae |  |  |
| **X179** | 0 | 0 | 727 | 2 | 4 | 1 | 731 | 3 | Annelida | Eunicidae |  |  |
| **X238** | 1 | 1 | 0 | 0 | 0 | 0 | 1 | 1 | Annelida | Orbiniidae |  |  |
| **X265** | 0 | 0 | 0 | 0 | 1 | 1 | 1 | 1 | Annelida | Spionidae |  |  |
| **X267** | 1 | 1 | 0 | 0 | 0 | 0 | 1 | 1 | Annelida | Eunicidae |  |  |
| **X286** | 0 | 0 | 0 | 0 | 1 | 1 | 1 | 1 | Annelida | Eunicidae |  |  |
| **X316** | 2 | 1 | 0 | 0 | 0 | 0 | 2 | 1 | Annelida | Spionidae |  |  |
| **X002** | 0 | 0 | 37 | 1 | 0 | 0 | 37 | 1 | Apicomplexa | Sarcocystidae |  |  |
| **X055** | 3387 | 5 | 2 | 1 | 916 | 3 | 4305 | 9 | Arthropoda | Daira perlata | BMOO 03530 | HM638029 |
| **X017** | 0 | 0 | 0 | 0 | 19 | 1 | 19 | 1 | Arthropoda | Harpacticoida | BMOO 14006 |  |
| **X100** | 0 | 0 | 18 | 1 | 0 | 0 | 18 | 1 | Arthropoda | Xanthidae | BMOO_00008 |  |
| **X117** | 214 | 8 | 0 | 0 | 1 | 1 | 215 | 9 | Arthropoda | Galathea mauritiana | BMOO_00448 | HM462506 |
| **X163** | 572 | 5 | 1846 | 3 | 217 | 2 | 2635 | 10 | Arthropoda | Pilodius pugil | BMOO_00480 |  |
| **X086** | 59 | 1 | 0 | 0 | 0 | 0 | 59 | 1 | Arthropoda | Chlorodiella | BMOO_00481 | JN107924 |
| **X121** | 185 | 1 | 1 | 1 | 0 | 0 | 186 | 2 | Arthropoda | Liomera bella | BMOO_00539 | HM751007 |
| **X159** | 69 | 2 | 0 | 0 | 0 | 0 | 69 | 2 | Arthropoda | Lysmata | BMOO_00669 |  |
| **X287** | 1 | 1 | 0 | 0 | 0 | 0 | 1 | 1 | Arthropoda | Ciliopagurus strigatus | BMOO_00698 | EF683559 |
| **X127** | 33 | 2 | 0 | 0 | 0 | 0 | 33 | 2 | Arthropoda | Dardanus gemmatus | BMOO_00786 | HM464937 |
| **X176** | 44 | 2 | 0 | 0 | 0 | 0 | 44 | 2 | Arthropoda | Trapezia serenei | BMOO_01003 |  |
| **X326** | 0 | 0 | 2 | 1 | 0 | 0 | 2 | 1 | Arthropoda | Menaethius monoceros | BMOO_01080 | JN107933 |
| **X146** | 188 | 1 | 0 | 0 | 0 | 0 | 188 | 1 | Arthropoda | Catoptrus inaequalis | BMOO_01305 |  |
| **X169** | 145 | 2 | 0 | 0 | 0 | 0 | 145 | 2 | Arthropoda | Sadayoshia edwardsii | BMOO_01334 | GQ260878 |
| **X201** | 2 | 1 | 0 | 0 | 0 | 0 | 2 | 1 | Arthropoda | Pagurixus ruber | BMOO_01462 | GQ260882 |
| **X152** | 1 | 1 | 0 | 0 | 68 | 2 | 69 | 3 | Arthropoda | Phylladiorhynchus | BMOO_01548 | HM466505 |
| **X067** | 0 | 0 | 0 | 0 | 12 | 1 | 12 | 1 | Arthropoda | Alpheus aff. paracrinitus | BMOO_01774 | HM466517 |
| **X260** | 1 | 1 | 0 | 0 | 0 | 0 | 1 | 1 | Arthropoda | Calcinus guamensis | BMOO_01984 | GQ260869 |
| **X171** | 14 | 1 | 0 | 0 | 0 | 0 | 14 | 1 | Arthropoda | Alpheopsis | BMOO_02259 |  |
| **X080** | 599 | 3 | 0 | 0 | 0 | 0 | 599 | 3 | Arthropoda | Galathea pilosa | BMOO_02291 | GQ260874 |
| **X078** | 38 | 1 | 0 | 0 | 0 | 0 | 38 | 1 | Arthropoda | Alpheus pachychirus | BMOO_02307 | JN679706 |
| **X124** | 47 | 1 | 107 | 1 | 0 | 0 | 154 | 2 | Arthropoda | Cuapetes cf. ensifrons | BMOO_02313 | HM465055 |
| **X138** | 27 | 1 | 0 | 0 | 31 | 1 | 58 | 2 | Arthropoda | Alpheus | BMOO_02323 | HM465957 |
| **X272** | 0 | 0 | 0 | 0 | 1 | 1 | 1 | 1 | Arthropoda | Viaderiana | BMOO_02348 |  |
| **X172** | 4 | 2 | 0 | 0 | 0 | 0 | 4 | 2 | Arthropoda | Leptodius sanguineus | BMOO_02351 |  |
| **X085** | 595 | 3 | 0 | 0 | 0 | 0 | 595 | 3 | Arthropoda | Pagurixus | BMOO_02483 |  |
| **X167** | 0 | 0 | 1022 | 1 | 0 | 0 | 1022 | 1 | Arthropoda | Thalamita | BMOO_02498 |  |
| **X180** | 40 | 3 | 0 | 0 | 14 | 1 | 54 | 4 | Arthropoda | Alpheus | BMOO_02620 | HM464905 |
| **X046** | 0 | 0 | 5 | 1 | 0 | 0 | 5 | 1 | Arthropoda | Luciferidae | BMOO_02627 |  |
| **X103** | 35 | 3 | 0 | 0 | 0 | 0 | 35 | 3 | Arthropoda | Decapoda | BMOO_02629 |  |
| **X262** | 1 | 1 | 0 | 0 | 0 | 0 | 1 | 1 | Arthropoda | Paraxanthias notatus | BMOO_02668 |  |
| **X164** | 3 | 1 | 0 | 0 | 0 | 0 | 3 | 1 | Arthropoda | Lybia | BMOO_02730 |  |
| **X083** | 8 | 1 | 0 | 0 | 0 | 0 | 8 | 1 | Arthropoda | Synalpheus paraneomeris | BMOO_02924 |  |
| **X166** | 57 | 2 | 0 | 0 | 0 | 0 | 57 | 2 | Arthropoda | Liomera rubra | BMOO_02926 | HM465963 |
| **X077** | 47 | 2 | 0 | 0 | 0 | 0 | 47 | 2 | Arthropoda | Alpheus edamensis | BMOO_02997 |  |
| **X081** | 0 | 0 | 11 | 1 | 0 | 0 | 11 | 1 | Arthropoda | Cardisoma carnifex | BMOO_03026 |  |
| **X197** | 12 | 1 | 0 | 0 | 0 | 0 | 12 | 1 | Arthropoda | Cyclodius ungulatus | BMOO_03030 | HM750965 |
| **X182** | 6 | 1 | 0 | 0 | 11 | 1 | 17 | 2 | Arthropoda | Alpheus aff. paracrinitus | BMOO_03251 |  |
| **X183** | 273 | 1 | 0 | 0 | 0 | 0 | 273 | 1 | Arthropoda | Clibanarius humilis | BMOO_03255 |  |
| **X147** | 17 | 2 | 510 | 3 | 0 | 0 | 527 | 5 | Arthropoda | Phylladiorhynchus | BMOO_03262 | JN107961 |
| **X106** | 1233 | 4 | 0 | 0 | 6 | 1 | 1239 | 5 | Arthropoda | Decapoda | BMOO_03297 |  |
| **X220** | 2 | 1 | 0 | 0 | 0 | 0 | 2 | 1 | Arthropoda | Euphausiidae | BMOO_03333 |  |
| **X021** | 0 | 0 | 18 | 1 | 0 | 0 | 18 | 1 | Arthropoda | Decapoda | BMOO_03355 |  |
| **X282** | 1 | 1 | 0 | 0 | 0 | 0 | 1 | 1 | Arthropoda | Percnon abbreviatum | BMOO_03523 | GQ260912 |
| **X253** | 1 | 1 | 0 | 0 | 0 | 0 | 1 | 1 | Arthropoda | Synalpheus | BMOO_03626 |  |
| **X136** | 1 | 1 | 0 | 0 | 69 | 1 | 70 | 2 | Arthropoda | Lachnopodus subacutus | BMOO_03639 |  |
| **X192** | 103 | 3 | 0 | 0 | 0 | 0 | 103 | 3 | Arthropoda | Pilodius areolatus | BMOO_03808 | HM751055 |
| **X060** | 3037 | 2 | 1 | 1 | 2 | 1 | 3040 | 4 | Arthropoda | Nannosquillidae | BMOO_03835 |  |
| **X275** | 1 | 1 | 0 | 0 | 0 | 0 | 1 | 1 | Arthropoda | Trapezia bella | BMOO_03989 |  |
| **X277** | 1 | 1 | 0 | 0 | 0 | 0 | 1 | 1 | Arthropoda | Medaeus elegans | BMOO_04008 |  |
| **X012** | 300 | 1 | 0 | 0 | 0 | 0 | 300 | 1 | Arthropoda | Saron marmoratus | BMOO_04169 |  |
| **X128** | 85 | 2 | 0 | 0 | 0 | 0 | 85 | 2 | Arthropoda | Alpheus aff. gracilipes | BMOO_04400 |  |
| **X090** | 46 | 1 | 0 | 0 | 0 | 0 | 46 | 1 | Arthropoda | Medaeus ornatus | BMOO_04421 | GQ260926 |
| **X061** | 41 | 1 | 0 | 0 | 0 | 0 | 41 | 1 | Arthropoda | Brachycarpus biunguiculatus | BMOO_04578 |  |
| **X306** | 1 | 1 | 0 | 0 | 0 | 0 | 1 | 1 | Arthropoda | Urocaridella antonbruuni | BMOO_04705 |  |
| **X139** | 46 | 3 | 0 | 0 | 0 | 0 | 46 | 3 | Arthropoda | Majidae | BMOO_04707 |  |
| **X118** | 232 | 3 | 0 | 0 | 0 | 0 | 232 | 3 | Arthropoda | Alpheus gracilipes | BMOO_04948 | HM465959 |
| **X093** | 0 | 0 | 0 | 0 | 9 | 1 | 9 | 1 | Arthropoda | Epialtidae | BMOO_05355 | GQ260908 |
| **X072** | 0 | 0 | 0 | 0 | 3 | 1 | 3 | 1 | Arthropoda | Synalpheus cf. coutierei | BMOO_05394 |  |
| **X084** | 0 | 0 | 47 | 1 | 1 | 1 | 48 | 2 | Arthropoda | Thalamita admete | BMOO_05512 | JQ180243 |
| **X296** | 2 | 1 | 0 | 0 | 0 | 0 | 2 | 1 | Arthropoda | Axiidae | BMOO_07680 |  |
| **X191** | 1 | 1 | 45 | 3 | 0 | 0 | 46 | 4 | Arthropoda | Liocarpilodes integerrimus | BMOO_07805 | HM465493 |
| **X073** | 0 | 0 | 0 | 0 | 13 | 1 | 13 | 1 | Arthropoda | Callianassidae | BMOO_08150 |  |
| **X160** | 703 | 3 | 0 | 0 | 0 | 0 | 703 | 3 | Arthropoda | Libystes | BMOO_08585 |  |
| **X076** | 18 | 3 | 0 | 0 | 0 | 0 | 18 | 3 | Arthropoda | Axiopsis | BMOO_08610 |  |
| **X161** | 143 | 1 | 0 | 0 | 0 | 0 | 143 | 1 | Arthropoda | Axianassa ngochoae | BMOO_09173 |  |
| **X088** | 248 | 4 | 1710 | 3 | 989 | 3 | 2947 | 10 | Arthropoda | Xanthidae | BMOO_09251 | HM464357 |
| **X045** | 1 | 1 | 35 | 1 | 0 | 0 | 36 | 2 | Arthropoda | Nikoides maldivensis | BMOO_09278 |  |
| **X308** | 0 | 0 | 0 | 0 | 1 | 1 | 1 | 1 | Arthropoda | Alpheopsis yaldwyni | BMOO_09282 |  |
| **X285** | 0 | 0 | 0 | 0 | 2 | 1 | 2 | 1 | Arthropoda | Portunidae | BMOO_09317 |  |
| **X095** | 2 | 1 | 0 | 0 | 6 | 1 | 8 | 2 | Arthropoda | Alpheus cf. obesomanus | BMOO_09796 |  |
| **X144** | 0 | 0 | 0 | 0 | 419 | 2 | 419 | 2 | Arthropoda | Gnathiidae | BMOO_09910 |  |
| **X048** | 29 | 1 | 0 | 0 | 0 | 0 | 29 | 1 | Arthropoda | Automate dolichognatha | BMOO_09912 |  |
| **X064** | 13 | 1 | 0 | 0 | 0 | 0 | 13 | 1 | Arthropoda | Alpheus | BMOO_10085 |  |
| **X254** | 0 | 0 | 2 | 1 | 0 | 0 | 2 | 1 | Arthropoda | Metapenaeopsis | BMOO_10187 |  |
| **X300** | 2 | 1 | 0 | 0 | 0 | 0 | 2 | 1 | Arthropoda | Synopia ultramarina | BMOO_10292 |  |
| **X033** | 9 | 1 | 0 | 0 | 0 | 0 | 9 | 1 | Arthropoda | Parambasia | BMOO_10608 |  |
| **X025** | 0 | 0 | 11 | 1 | 0 | 0 | 11 | 1 | Arthropoda | Amphilochus | BMOO_10734 |  |
| **X320** | 0 | 0 | 0 | 0 | 1 | 1 | 1 | 1 | Arthropoda | Yhi yindi | BMOO_10849 | HM466373 |
| **X332** | 1 | 1 | 0 | 0 | 0 | 0 | 1 | 1 | Arthropoda | Alpheus lottini | BMOO_10894 |  |
| **X056** | 0 | 0 | 7 | 1 | 0 | 0 | 7 | 1 | Arthropoda | Hapalocarcinus | BMOO_10895 |  |
| **X119** | 2392 | 4 | 0 | 0 | 0 | 0 | 2392 | 4 | Arthropoda | Synalpheus | BMOO_10903 | HM465529 |
| **X094** | 1631 | 4 | 0 | 0 | 0 | 0 | 1631 | 4 | Arthropoda | Decapoda | BMOO_11028 |  |
| **X082** | 280 | 3 | 5 | 1 | 0 | 0 | 285 | 4 | Arthropoda | Brachyura | BMOO_11158 |  |
| **X043** | 8 | 1 | 0 | 0 | 0 | 0 | 8 | 1 | Arthropoda | Thor | BMOO_11253 | GQ260962 |
| **X132** | 1 | 1 | 356 | 1 | 0 | 0 | 357 | 2 | Arthropoda | Thor | BMOO_11255 | HM466584 |
| **X051** | 31 | 1 | 1 | 1 | 0 | 0 | 32 | 2 | Arthropoda | Menaethius | BMOO_11396 | HM462560 |
| **X140** | 618 | 4 | 3 | 1 | 0 | 0 | 621 | 5 | Arthropoda | Paguroidea | BMOO_11424 | HM466519 |
| **X189** | 40 | 1 | 0 | 0 | 6 | 1 | 46 | 2 | Arthropoda | Brachyura | BMOO_11450 |  |
| **X271** | 0 | 0 | 0 | 0 | 6 | 1 | 6 | 1 | Arthropoda | Portunidae | BMOO_11672 |  |
| **X174** | 70 | 1 | 0 | 0 | 0 | 0 | 70 | 1 | Arthropoda | Brachyura | BMOO_11685 | JN679777 |
| **X157** | 225 | 2 | 0 | 0 | 4 | 1 | 229 | 3 | Arthropoda | Brachyura | BMOO_11869 |  |
| **X122** | 9 | 1 | 674 | 3 | 0 | 0 | 683 | 4 | Arthropoda | Brachyura | BMOO_12375 | HM465541 |
| **X156** | 135 | 3 | 2 | 1 | 0 | 0 | 137 | 4 | Arthropoda | Brachyura | BMOO_12503 |  |
| **X184** | 222 | 1 | 0 | 0 | 1 | 1 | 223 | 2 | Arthropoda | Brachyura | BMOO_12635 |  |
| **X175** | 0 | 0 | 18 | 1 | 0 | 0 | 18 | 1 | Arthropoda | Xanthidae | BMOO_12826 | JN679619 |
| **X190** | 1641 | 6 | 0 | 0 | 0 | 0 | 1641 | 6 | Arthropoda | Pilodius flavus | BMOO-05342 | GQ260938 |
| **X111** | 947 | 1 | 0 | 0 | 0 | 0 | 947 | 1 | Arthropoda | Quadrimaera quadrimanus | BMOO-10613 |  |
| **X309** | 4 | 1 | 0 | 0 | 0 | 0 | 4 | 1 | Arthropoda | Alpheidae | DL218Z |  |
| **X131** | 11 | 1 | 175 | 2 | 46 | 1 | 232 | 4 | Arthropoda | Decapoda | DL534 |  |
| **X005** | 30 | 2 | 0 | 0 | 0 | 0 | 30 | 2 | Arthropoda | Conodocera | GDHBMOO-1 |  |
| **X057** | 8 | 3 | 0 | 0 | 0 | 0 | 8 | 3 | Arthropoda | Calcinus morgani | MBIO2320 | HM465864 |
| **X058** | 12 | 3 | 0 | 0 | 0 | 0 | 12 | 3 | Arthropoda | Alpheus malleodigitus | MBIO3100 |  |
| **X112** | 359 | 4 | 1 | 1 | 0 | 0 | 360 | 5 | Arthropoda | Xanthidae | MBIO4218 |  |
| **X089** | 7 | 2 | 0 | 0 | 0 | 0 | 7 | 2 | Arthropoda | Galatheidae | XMOO_0036 |  |
| **X116** | 146 | 4 | 17 | 1 | 7 | 1 | 170 | 6 | Arthropoda | Paguridae | XMOO_0476 | HM464204 |
| **X142** | 1064 | 4 | 0 | 0 | 21 | 3 | 1085 | 7 | Arthropoda | Maxillipoda | XMOO_1064 |  |
| **X241** | 0 | 0 | 0 | 0 | 1 | 1 | 1 | 1 | Arthropoda | Macrosetella gracilis |  | DQ989149 |
| **X098** | 18 | 2 | 0 | 0 | 0 | 0 | 18 | 2 | Arthropoda | Dardanus lagopodes |  | EF683577 |
| **X314** | 2 | 1 | 0 | 0 | 0 | 0 | 2 | 1 | Arthropoda | Caridea |  | HM465998 |
| **X289** | 1 | 1 | 0 | 0 | 0 | 0 | 1 | 1 | Arthropoda | Stenopus hispidus |  | JN399096 |
| **X003** | 1 | 1 | 0 | 0 | 6 | 1 | 7 | 2 | Arthropoda | Calanoida |  |  |
| **X007** | 0 | 0 | 10 | 2 | 11 | 1 | 21 | 3 | Arthropoda | Miraciidae |  |  |
| **X010** | 4 | 2 | 6 | 3 | 2 | 1 | 12 | 6 | Arthropoda | Cyclopettidae |  |  |
| **X026** | 4 | 2 | 1 | 1 | 3 | 2 | 8 | 5 | Arthropoda | Miraciidae |  |  |
| **X029** | 8 | 3 | 0 | 0 | 2 | 1 | 10 | 4 | Arthropoda | Miraciidae |  |  |
| **X030** | 0 | 0 | 0 | 0 | 13 | 1 | 13 | 1 | Arthropoda | Decapoda |  |  |
| **X038** | 98 | 3 | 0 | 0 | 2 | 1 | 100 | 4 | Arthropoda | Alpheidae |  |  |
| **X041** | 10 | 1 | 0 | 0 | 0 | 0 | 10 | 1 | Arthropoda | Decapoda |  |  |
| **X044** | 1 | 1 | 0 | 0 | 8 | 1 | 9 | 2 | Arthropoda | Decapoda |  |  |
| **X052** | 3 | 1 | 1 | 1 | 1 | 1 | 5 | 3 | Arthropoda | Verrucidae |  |  |
| **X053** | 5 | 2 | 0 | 0 | 0 | 0 | 5 | 2 | Arthropoda | Decapoda |  |  |
| **X068** | 10 | 1 | 0 | 0 | 0 | 0 | 10 | 1 | Arthropoda | Alpheidae |  |  |
| **X079** | 7 | 1 | 0 | 0 | 0 | 0 | 7 | 1 | Arthropoda | Decapoda |  |  |
| **X092** | 9 | 1 | 0 | 0 | 0 | 0 | 9 | 1 | Arthropoda | Xanthidae |  |  |
| **X096** | 7 | 1 | 0 | 0 | 124 | 2 | 131 | 3 | Arthropoda | Alpheidae |  |  |
| **X107** | 0 | 0 | 0 | 0 | 6 | 1 | 6 | 1 | Arthropoda | Miraciidae |  |  |
| **X108** | 16 | 2 | 0 | 0 | 0 | 0 | 16 | 2 | Arthropoda | Euchaetidae |  |  |
| **X114** | 7 | 2 | 0 | 0 | 0 | 0 | 7 | 2 | Arthropoda | Alpheidae |  |  |
| **X115** | 0 | 0 | 0 | 0 | 299 | 1 | 299 | 1 | Arthropoda | Oncaeidae |  |  |
| **X120** | 7 | 1 | 0 | 0 | 0 | 0 | 7 | 1 | Arthropoda | Xanthidae |  |  |
| **X143** | 372 | 1 | 0 | 0 | 0 | 0 | 372 | 1 | Arthropoda | Palaemonidae |  |  |
| **X149** | 0 | 0 | 29 | 2 | 0 | 0 | 29 | 2 | Arthropoda | Hippolytidae |  |  |
| **X151** | 0 | 0 | 0 | 0 | 170 | 1 | 170 | 1 | Arthropoda | Maxillipoda |  |  |
| **X155** | 32 | 3 | 8 | 1 | 5 | 1 | 45 | 5 | Arthropoda | Maxillipoda |  |  |
| **X168** | 14 | 2 | 0 | 0 | 3 | 1 | 17 | 3 | Arthropoda | Acartiidae |  |  |
| **X177** | 1 | 1 | 0 | 0 | 10 | 1 | 11 | 2 | Arthropoda | Decapoda |  |  |
| **X195** | 28 | 2 | 24 | 1 | 4 | 1 | 56 | 4 | Arthropoda | Miraciidae |  |  |
| **X196** | 33 | 2 | 0 | 0 | 0 | 0 | 33 | 2 | Arthropoda | Alpheidae |  |  |
| **X198** | 3 | 1 | 0 | 0 | 0 | 0 | 3 | 1 | Arthropoda | Xanthidae |  |  |
| **X206** | 1 | 1 | 0 | 0 | 0 | 0 | 1 | 1 | Arthropoda | Miraciidae |  |  |
| **X218** | 3 | 1 | 0 | 0 | 0 | 0 | 3 | 1 | Arthropoda | Decapoda |  |  |
| **X223** | 0 | 0 | 0 | 0 | 1 | 1 | 1 | 1 | Arthropoda | Maxillipoda |  |  |
| **X224** | 0 | 0 | 1 | 1 | 0 | 0 | 1 | 1 | Arthropoda | Calanoida |  |  |
| **X226** | 0 | 0 | 0 | 0 | 1 | 1 | 1 | 1 | Arthropoda | Calanoida |  |  |
| **X227** | 0 | 0 | 0 | 0 | 2 | 1 | 2 | 1 | Arthropoda | Cyclopidae |  |  |
| **X230** | 0 | 0 | 0 | 0 | 3 | 1 | 3 | 1 | Arthropoda | Oncaeidae |  |  |
| **X235** | 1 | 1 | 0 | 0 | 0 | 0 | 1 | 1 | Arthropoda | Cyclopettidae |  |  |
| **X237** | 1 | 1 | 0 | 0 | 0 | 0 | 1 | 1 | Arthropoda | Malacostraca |  |  |
| **X240** | 0 | 0 | 2 | 1 | 0 | 0 | 2 | 1 | Arthropoda | Domeciidae |  |  |
| **X243** | 2 | 1 | 0 | 0 | 0 | 0 | 2 | 1 | Arthropoda | Calanoida |  |  |
| **X244** | 2 | 1 | 0 | 0 | 0 | 0 | 2 | 1 | Arthropoda | Siphonostomatoida |  |  |
| **X245** | 0 | 0 | 0 | 0 | 3 | 1 | 3 | 1 | Arthropoda | Maxillipoda |  |  |
| **X246** | 0 | 0 | 0 | 0 | 1 | 1 | 1 | 1 | Arthropoda | Palaemonidae |  |  |
| **X247** | 2 | 1 | 0 | 0 | 0 | 0 | 2 | 1 | Arthropoda | Clausocalanidae |  |  |
| **X249** | 1 | 1 | 0 | 0 | 0 | 0 | 1 | 1 | Arthropoda | Oncaeidae |  |  |
| **X255** | 2 | 1 | 0 | 0 | 0 | 0 | 2 | 1 | Arthropoda | Calanidae |  |  |
| **X264** | 2 | 1 | 0 | 0 | 0 | 0 | 2 | 1 | Arthropoda | Harpacticoida |  |  |
| **X278** | 0 | 0 | 2 | 1 | 0 | 0 | 2 | 1 | Arthropoda | Malacostraca |  |  |
| **X279** | 1 | 1 | 0 | 0 | 0 | 0 | 1 | 1 | Arthropoda | Miraciidae |  |  |
| **X281** | 2 | 1 | 0 | 0 | 0 | 0 | 2 | 1 | Arthropoda | Diogenidae |  |  |
| **X288** | 0 | 0 | 1 | 1 | 0 | 0 | 1 | 1 | Arthropoda | Decapoda |  |  |
| **X295** | 2 | 1 | 0 | 0 | 0 | 0 | 2 | 1 | Arthropoda | Hippolytidae |  |  |
| **X303** | 2 | 1 | 0 | 0 | 0 | 0 | 2 | 1 | Arthropoda | Arthropoda |  |  |
| **X304** | 2 | 1 | 0 | 0 | 0 | 0 | 2 | 1 | Arthropoda | Canthocamptidae |  |  |
| **X305** | 0 | 0 | 0 | 0 | 1 | 1 | 1 | 1 | Arthropoda | Alpheidae |  |  |
| **X310** | 0 | 0 | 4 | 1 | 0 | 0 | 4 | 1 | Arthropoda | Lithoglyptidae |  |  |
| **X312** | 0 | 0 | 2 | 1 | 0 | 0 | 2 | 1 | Arthropoda | Miraciidae |  |  |
| **X315** | 3 | 1 | 0 | 0 | 0 | 0 | 3 | 1 | Arthropoda | Maxillipoda |  |  |
| **X317** | 5 | 1 | 0 | 0 | 0 | 0 | 5 | 1 | Arthropoda | Decapoda |  |  |
| **X319** | 0 | 0 | 0 | 0 | 2 | 1 | 2 | 1 | Arthropoda | Decapoda |  |  |
| **X329** | 1 | 1 | 0 | 0 | 0 | 0 | 1 | 1 | Arthropoda | Paguridae |  |  |
| **X331** | 2 | 1 | 0 | 0 | 0 | 0 | 2 | 1 | Arthropoda | Decapoda |  |  |
| **X333** | 1 | 1 | 0 | 0 | 0 | 0 | 1 | 1 | Arthropoda | Hippolytidae |  |  |
| **X334** | 2 | 1 | 0 | 0 | 0 | 0 | 2 | 1 | Arthropoda | Euphausiidae |  |  |
| **X231** | 2 | 1 | 0 | 0 | 0 | 0 | 2 | 1 | Chaetognatha | Sagitta | BMOO_08055 |  |
| **X252** | 0 | 0 | 0 | 0 | 2 | 1 | 2 | 1 | Chordata | Oplopomus oplopomus | FLMOO_1160 | HQ536661 |
| **X130** | 356 | 2 | 0 | 0 | 0 | 0 | 356 | 2 | Chordata | Gobiidae | FLMOO_974 |  |
| **X293** | 1 | 1 | 0 | 0 | 0 | 0 | 1 | 1 | Chordata | Apogonidae | FLMOO156 |  |
| **X276** | 2 | 1 | 0 | 0 | 0 | 0 | 2 | 1 | Chordata | Cheilio inermis | MBIO1457 | AY850742 |
| **X283** | 0 | 0 | 1 | 1 | 0 | 0 | 1 | 1 | Chordata | Forcipiger flavissimus | MBIO653 | FJ583406 |
| **X292** | 1 | 1 | 0 | 0 | 0 | 0 | 1 | 1 | Chordata | Gnatholepis cauerensis | Mp0421 | AF391376 |
| **X153** | 623 | 1 | 1 | 1 | 2 | 1 | 626 | 3 | Chordata | Scarus globiceps | Mp0424 | JQ432102 |
| **X170** | 0 | 0 | 16 | 1 | 0 | 0 | 16 | 1 | Chordata | Scarus psittacus | Mp0425 | JQ432113 |
| **X291** | 1 | 1 | 2 | 1 | 0 | 0 | 3 | 2 | Chordata | Pseudogramma polyacanthum | MParis0012 | JQ350284 |
| **X158** | 9 | 3 | 5 | 2 | 3 | 1 | 17 | 6 | Chordata | Dascyllus flavicaudus | MParis0021 | JQ431678 |
| **X321** | 0 | 0 | 1 | 1 | 0 | 0 | 1 | 1 | Chordata | Chromis iomelas | MParis0056 | JF434887 |
| **X087** | 0 | 0 | 4 | 1 | 0 | 0 | 4 | 1 | Chordata | Parupeneus multifasciatus | MParis0063 | FJ237887 |
| **X322** | 0 | 0 | 0 | 0 | 2 | 1 | 2 | 1 | Chordata | Pseudocheilinus hexataenia | MParis0075 | JQ432058 |
| **X284** | 2 | 1 | 0 | 0 | 0 | 0 | 2 | 1 | Chordata | Ctenochaetus striatus | Mparis0091 | HM034214 |
| **X101** | 0 | 0 | 0 | 0 | 3 | 1 | 3 | 1 | Chordata | Zebrasoma scopas | Mparis0107 | FJ584269 |
| **X059** | 0 | 0 | 0 | 0 | 5 | 1 | 5 | 1 | Chordata | Gobiodon quinquestrigatus | Mparis0117 | JQ431765 |
| **X069** | 12 | 2 | 0 | 0 | 0 | 0 | 12 | 2 | Chordata | Cirripectes quagga | MParis0202 | JQ431647 |
| **X109** | 0 | 0 | 8 | 1 | 0 | 0 | 8 | 1 | Chordata | Sebastapistes tinkhami | MParis0210 | JQ432148 |
| **X074** | 4 | 2 | 0 | 0 | 0 | 0 | 4 | 2 | Chordata | Cirripectes variolosus | MParis0213 | JQ431649 |
| **X054** | 3 | 2 | 0 | 0 | 5 | 1 | 8 | 3 | Chordata | Stegastes nigricans | MParis0231 | JQ432166 |
| **X065** | 0 | 0 | 14 | 1 | 0 | 0 | 14 | 1 | Chordata | Antennarius coccineus | MParis0246 | GU188486 |
| **X049** | 24 | 1 | 0 | 0 | 0 | 0 | 24 | 1 | Chordata | Callogobius sclateri | MParis0251-A | JQ431512 |
| **X050** | 1 | 1 | 1 | 1 | 7 | 1 | 9 | 3 | Chordata | Ctenogobiops feroculus | MParis0263 | JQ431671 |
| **X062** | 0 | 0 | 4 | 1 | 0 | 0 | 4 | 1 | Chordata | Eviota | MParis0271 | JQ431736 |
| **X040** | 47 | 1 | 0 | 0 | 1 | 1 | 48 | 2 | Chordata | Callionymus simplicicornis | MParis0913 | JQ431511 |
| **X113** | 0 | 0 | 26 | 1 | 82 | 2 | 108 | 3 | Chordata | Myripristis kuntee | MParis0966 | HM034231 |
| **X307** | 1 | 1 | 0 | 0 | 0 | 0 | 1 | 1 | Chordata | Pomacentrus pavo | Mparis1002-A | JQ432030 |
| **X126** | 0 | 0 | 0 | 0 | 147 | 1 | 147 | 1 | Chordata | Fusigobius neophytus | Mparis1005 | JQ431664 |
| **X102** | 5 | 2 | 0 | 0 | 0 | 0 | 5 | 2 | Chordata | Chromis viridis | MParis523 | FJ583164 |
| **X063** | 16 | 2 | 0 | 0 | 0 | 0 | 16 | 2 | Chordata | Plectroglyphidodon leucozonus | MParis529 | JF435085 |
| **X290** | 1 | 1 | 0 | 0 | 0 | 0 | 1 | 1 | Chordata | Pomachromis fuscidorsalis | MParis650 | JF435138 |
| **X266** | 1 | 1 | 0 | 0 | 0 | 0 | 1 | 1 | Chordata | Sebastapistes fowleri | MParis699 | JQ432143 |
| **X070** | 0 | 0 | 3 | 1 | 0 | 0 | 3 | 1 | Chordata | Cheilinus chlorourus | MParis712-A | JF434848 |
| **X110** | 33 | 1 | 0 | 0 | 0 | 0 | 33 | 1 | Chordata | Novaculichthys taeniourus | MParis816 | FJ583741 |
| **X001** | 946 | 3 | 17 | 1 | 0 | 0 | 963 | 4 | Chordata | Asymmetron lucayanum |  | AB240560 |
| **X325** | 0 | 0 | 0 | 0 | 3 | 1 | 3 | 1 | Chordata | Schindleria pietschmanni |  | AF391335 |
| **X066** | 4 | 1 | 0 | 0 | 0 | 0 | 4 | 1 | Chordata | Arothron meleagris |  | FJ582876 |
| **X178** | 225 | 1 | 0 | 0 | 0 | 0 | 225 | 1 | Chordata | Gobiidae |  |  |
| **X186** | 0 | 0 | 0 | 0 | 186 | 1 | 186 | 1 | Chordata | Gobiidae |  |  |
| **X268** | 0 | 0 | 1 | 1 | 0 | 0 | 1 | 1 | Chordata | Scorpaenidae |  |  |
| **X294** | 0 | 0 | 1 | 1 | 0 | 0 | 1 | 1 | Chordata | Perciformes |  |  |
| **X330** | 3 | 2 | 0 | 0 | 0 | 0 | 3 | 2 | Chordata | Perciformes |  |  |
| **X256** | 0 | 0 | 0 | 0 | 1 | 1 | 1 | 1 | Cnidaria | Pocillopora | BMOO_03451 | EF526303 |
| **X031** | 7 | 1 | 0 | 0 | 0 | 0 | 7 | 1 | Cnidaria | Hydrozoa |  |  |
| **X047** | 6 | 2 | 0 | 0 | 0 | 0 | 6 | 2 | Cnidaria | Hydroida |  |  |
| **X075** | 3 | 2 | 0 | 0 | 1 | 1 | 4 | 3 | Cnidaria | Hydroida |  |  |
| **X091** | 0 | 0 | 3 | 1 | 0 | 0 | 3 | 1 | Cnidaria | Campanulariidae |  |  |
| **X099** | 3 | 1 | 0 | 0 | 0 | 0 | 3 | 1 | Cnidaria | Hydroida |  |  |
| **X185** | 8 | 2 | 0 | 0 | 0 | 0 | 8 | 2 | Cnidaria | Hydrozoa |  |  |
| **X257** | 2 | 1 | 0 | 0 | 0 | 0 | 2 | 1 | Cnidaria | Hydrozoa |  |  |
| **X297** | 1 | 1 | 0 | 0 | 0 | 0 | 1 | 1 | Cnidaria | Hydrozoa |  |  |
| **X298** | 0 | 0 | 0 | 0 | 3 | 1 | 3 | 1 | Cnidaria | Agalmatidae |  |  |
| **X225** | 5 | 1 | 0 | 0 | 0 | 0 | 5 | 1 | Echinodermata | Echinometra mathaei | XMOO_0403 | GU670184 |
| **X022** | 1 | 1 | 2 | 2 | 20 | 1 | 23 | 4 | Echinodermata | Acanthaster planci |  | FM174498 |
| **X165** | 16 | 1 | 81 | 1 | 0 | 0 | 97 | 2 | Hemichordata | Ptychoderidae |  |  |
| **X181** | 95 | 1 | 0 | 0 | 0 | 0 | 95 | 1 | Hemichordata | Ptychoderidae |  |  |
| **X269** | 5 | 1 | 0 | 0 | 0 | 0 | 5 | 1 | Mollusca | Octopus cyanea | BMOO_00884 | AB430534 |
| **X071** | 0 | 0 | 0 | 0 | 10 | 1 | 10 | 1 | Mollusca | Phenacolepadidae | BMOO_10505 |  |
| **X188** | 0 | 0 | 41 | 2 | 0 | 0 | 41 | 2 | Mollusca | Deniatys dentifer | BMOO-02659 |  |
| **X135** | 128 | 2 | 0 | 0 | 0 | 0 | 128 | 2 | Mollusca | Octopodidae |  |  |
| **X173** | 0 | 0 | 2 | 1 | 10 | 1 | 12 | 2 | Mollusca | Octopodidae |  |  |
| **X203** | 1 | 1 | 0 | 0 | 0 | 0 | 1 | 1 | Nemotoda | Aphelenchoididae |  |  |
| **X233** | 1 | 1 | 0 | 0 | 0 | 0 | 1 | 1 | Platyhelminthes | Callioplanidae |  |  |
| **X034** | 1 | 1 | 4 | 3 | 0 | 0 | 5 | 4 | Porifera | Microciona prolifera |  | AJ704978 |
| **X273** | 1 | 1 | 0 | 0 | 0 | 0 | 1 | 1 | Porifera | Haliclona tubifera |  | EF519624 |
| **X219** | 2 | 1 | 0 | 0 | 0 | 0 | 2 | 1 | Porifera | Axinellidae |  |  |
| **X239** | 1 | 1 | 0 | 0 | 0 | 0 | 1 | 1 | Porifera | Mycalidae |  |  |
| **X274** | 1 | 1 | 0 | 0 | 0 | 0 | 1 | 1 | Porifera | Plakinidae |  |  |
| **X328** | 3 | 1 | 1 | 1 | 0 | 0 | 4 | 2 | Porifera | Plakinidae |  |  |
| **X280** | 0 | 0 | 1 | 1 | 0 | 0 | 1 | 1 | Rhodophyta | Delesseriaceae |  |  |
| **X301** | 0 | 0 | 1 | 1 | 0 | 0 | 1 | 1 | Rhodophyta | Rhodomelaceae |  |  |
| **X302** | 0 | 0 | 3 | 1 | 0 | 0 | 3 | 1 | Rhodophyta | Hypneaceae |  |  |
| **X311** | 1 | 1 | 0 | 0 | 0 | 0 | 1 | 1 | Rhodophyta | Ceramiaceae |  |  |
| **X009** | 0 | 0 | 4 | 1 | 0 | 0 | 4 | 1 | Sipuncula | Aspidosiphonidae | BMOO_02415 |  |
| **X019** | 0 | 0 | 30 | 2 | 0 | 0 | 30 | 2 | Sipuncula | Aspidosiphonidae |  |  |
| **X200** | 0 | 0 | 2 | 1 | 0 | 0 | 2 | 1 | Sipuncula | Aspidosiphonidae |  |  |
| **X234** | 0 | 0 | 1 | 1 | 0 | 0 | 1 | 1 | Sipuncula | Phascolosomatidae |  |  |
| **X248** | 0 | 0 | 1 | 1 | 0 | 0 | 1 | 1 | Sipuncula | Phascolosomatidae |  |  |
| **X004** | 0 | 0 | 0 | 0 | 4 | 1 | 4 | 1 | Unknown |  |  |  |
| **X006** | 0 | 0 | 0 | 0 | 7 | 1 | 7 | 1 | Unknown |  |  |  |
| **X008** | 3 | 2 | 1 | 1 | 2 | 1 | 6 | 4 | Unknown |  |  |  |
| **X011** | 8 | 2 | 0 | 0 | 0 | 0 | 8 | 2 | Unknown |  |  |  |
| **X014** | 29 | 1 | 0 | 0 | 19 | 1 | 48 | 2 | Unknown |  |  |  |
| **X018** | 0 | 0 | 6 | 1 | 0 | 0 | 6 | 1 | Unknown |  |  |  |
| **X020** | 1 | 1 | 0 | 0 | 3 | 1 | 4 | 2 | Unknown |  |  |  |
| **X024** | 0 | 0 | 5 | 1 | 0 | 0 | 5 | 1 | Unknown |  |  |  |
| **X027** | 1 | 1 | 2 | 1 | 0 | 0 | 3 | 2 | Unknown |  |  |  |
| **X028** | 0 | 0 | 0 | 0 | 4 | 1 | 4 | 1 | Unknown |  |  |  |
| **X042** | 0 | 0 | 0 | 0 | 8 | 1 | 8 | 1 | Unknown |  |  |  |
| **X097** | 1 | 1 | 2 | 1 | 0 | 0 | 3 | 2 | Unknown |  |  |  |
| **X104** | 4 | 1 | 0 | 0 | 0 | 0 | 4 | 1 | Unknown |  |  |  |
| **X105** | 8 | 1 | 0 | 0 | 18 | 1 | 26 | 2 | Unknown |  |  |  |
| **X123** | 79 | 6 | 27 | 4 | 18 | 2 | 124 | 12 | Unknown |  |  |  |
| **X137** | 295 | 1 | 0 | 0 | 2 | 1 | 297 | 2 | Unknown |  |  |  |
| **X141** | 0 | 0 | 0 | 0 | 52 | 1 | 52 | 1 | Unknown |  |  |  |
| **X148** | 0 | 0 | 36 | 1 | 0 | 0 | 36 | 1 | Unknown |  |  |  |
| **X162** | 51 | 1 | 0 | 0 | 0 | 0 | 51 | 1 | Unknown |  |  |  |
| **X187** | 0 | 0 | 30 | 1 | 0 | 0 | 30 | 1 | Unknown |  |  |  |
| **X194** | 0 | 0 | 0 | 0 | 35 | 1 | 35 | 1 | Unknown |  |  |  |
| **X199** | 5 | 2 | 3 | 2 | 0 | 0 | 8 | 4 | Unknown |  |  |  |
| **X202** | 0 | 0 | 1 | 1 | 0 | 0 | 1 | 1 | Unknown |  |  |  |
| **X204** | 1 | 1 | 0 | 0 | 0 | 0 | 1 | 1 | Unknown |  |  |  |
| **X205** | 1 | 1 | 0 | 0 | 0 | 0 | 1 | 1 | Unknown |  |  |  |
| **X207** | 0 | 0 | 1 | 1 | 0 | 0 | 1 | 1 | Unknown |  |  |  |
| **X208** | 0 | 0 | 2 | 1 | 0 | 0 | 2 | 1 | Unknown |  |  |  |
| **X209** | 0 | 0 | 1 | 1 | 0 | 0 | 1 | 1 | Unknown |  |  |  |
| **X210** | 0 | 0 | 1 | 1 | 0 | 0 | 1 | 1 | Unknown |  |  |  |
| **X211** | 0 | 0 | 0 | 0 | 1 | 1 | 1 | 1 | Unknown |  |  |  |
| **X212** | 2 | 1 | 0 | 0 | 0 | 0 | 2 | 1 | Unknown |  |  |  |
| **X214** | 2 | 1 | 0 | 0 | 0 | 0 | 2 | 1 | Unknown |  |  |  |
| **X215** | 13 | 1 | 0 | 0 | 0 | 0 | 13 | 1 | Unknown |  |  |  |
| **X216** | 0 | 0 | 1 | 1 | 0 | 0 | 1 | 1 | Unknown |  |  |  |
| **X217** | 0 | 0 | 1 | 1 | 0 | 0 | 1 | 1 | Unknown |  |  |  |
| **X221** | 1 | 1 | 0 | 0 | 0 | 0 | 1 | 1 | Unknown |  |  |  |
| **X222** | 1 | 1 | 0 | 0 | 0 | 0 | 1 | 1 | Unknown |  |  |  |
| **X228** | 0 | 0 | 0 | 0 | 2 | 1 | 2 | 1 | Unknown |  |  |  |
| **X229** | 1 | 1 | 0 | 0 | 0 | 0 | 1 | 1 | Unknown |  |  |  |
| **X232** | 1 | 1 | 0 | 0 | 0 | 0 | 1 | 1 | Unknown |  |  |  |
| **X236** | 0 | 0 | 1 | 1 | 0 | 0 | 1 | 1 | Unknown |  |  |  |
| **X250** | 0 | 0 | 0 | 0 | 1 | 1 | 1 | 1 | Unknown |  |  |  |
| **X251** | 1 | 1 | 0 | 0 | 0 | 0 | 1 | 1 | Unknown |  |  |  |
| **X258** | 0 | 0 | 1 | 1 | 0 | 0 | 1 | 1 | Unknown |  |  |  |
| **X261** | 1 | 1 | 0 | 0 | 0 | 0 | 1 | 1 | Unknown |  |  |  |
| **X263** | 1 | 1 | 0 | 0 | 0 | 0 | 1 | 1 | Unknown |  |  |  |
| **X270** | 0 | 0 | 1 | 1 | 0 | 0 | 1 | 1 | Unknown |  |  |  |
| **X313** | 1 | 1 | 0 | 0 | 3 | 2 | 4 | 3 | Unknown |  |  |  |
| **X318** | 0 | 0 | 2 | 2 | 0 | 0 | 2 | 2 | Unknown |  |  |  |
| **X323** | 1 | 1 | 0 | 0 | 1 | 1 | 2 | 2 | Unknown |  |  |  |
| **X324** | 1 | 1 | 0 | 0 | 0 | 0 | 1 | 1 | Unknown |  |  |  |
| **X327** | 3 | 1 | 0 | 0 | 0 | 0 | 3 | 1 | Unknown |  |  |  |
