# Supplementary material for: A new versatile primer set targeting a short fragment of the mitochondrial COI region for metabarcoding metazoan diversity: application for characterizing coral reef fish gut contents
Source: Front Zool. 2013 Jun 14;10:34. doi: 10.1186/1742-9994-10-34 (PMC3686579; doi:10.1186/1742-9994-10-34)
Supplement: Additional file 6 — Fasta formatted alignment of OTU representative sequences. See Additional file 5 for taxonomic identification. [file 1742-9994-10-34-S6.docx]

**Additional file 6** Fasta formatted alignment of OTU representative sequences. See Additional file 5 for taxonomic identification.

>x1_G6YZTIE02D7NXL

---TTAGCCGGCAACATTGCCCACGCTGGAGCATCTGTCGATTTAGCTATTTTTTCTCTCCATTTAGCGGGGGTCTCGTCAATTCTAGGTGCTATTAATTTCATCACGACGATTCATAATATACGTGCA---AGCGTTGAATGAAATCGTGTACCCCTATTCGTATGATCTATTTGAGTAACTGCTTATTTACTACTTCTCTCACTACCGGTTCTTGCCGGAGCCATTACCATACTCCTTACAGATCGAAACATCAATACTACCTTTTTCGACCCATCAGGAGGGGGAGATCCTATCTTATATGAACACTTA----

>x2_G6YZTIE01B9OLV

TTATCAACTTCTCTAATGGCTTTATCTCCTGTATCTATAGATCAGATGATTGCAGCTTTAGCTATTGTTGGTATTTCTAGCTTACTATCTTCTCTAAATTTCTTAACTACAACTTGGTTCTTA---GGTTTCTGTATGAACGATAAAGCTAATGCTGTATTCGTTTATGCTATTCTATTTACTGCAGTTATGTTAATTTGCACTCTACCTATTTTAACAGCTGGTCTTGTTATGATTGTACTAGATTTACATCTAAACACTCATTTCTACGATCCATTCTTTAATGGAGATCCTGTATTATACCAACATCTGTTC-

>x3_G6YZTIE01AQASN

--TTTGAGTGCGAATATTGCCCATAGGGGAGCTTCGGTAGATTTTGCTATTTTCTCTTTGCATTTAGCTGGGGTTTCATCTCTTCTAGGGGCCGTAAATTTCATTAGCACCCTAGGAAACTTGCGAATCTTTGGAATACTGCTAGATCGTATGCCTCTGTTTGCCTGGGCTGTAATAGTTACAGCAGTTTTGCTTTTGTTATCTTTGCCCGTATTAGCTGGTGCTATTACAATACTTCTAACAGACCGCAATTTAAATAGGACTTTCTACGACGTGGCCGGCGGTGGAGACCCAGTTCTATATCAGCATTTATT--

>x4_G6YZTIE01AQNIT

--GCTGTCAGATAGAGTATTTCATAGGGGGCCAGCCGTTGATATGGGTATTTTTTCTTTGCACATTGCTGGGGTTTCTTCTTTACTGGGTGCTATAAATTTTATAGTAACTTTTTTAAAACTAAAAAATAGAAAT---AGTTTGATTATGTCTCCCCTGTTTTCTTGGTCTATTTTTATTGCTGCGTATTTACTTTTAACAACGCTGCCAGTATTAGCTGGGGGTATTACAATACTTTTAATAGACCGAAACTTCAACACTTGTTTTTTTAACCCTTC--------------------------------------

>x5_G6YZTIE01BJTQN

--ACTTTCAGGCCTAGTCGGTCAAAACGGACCAAGAATGGACCTTGCCATCTTT---CTTCATTTAGCAGGTGCCTCATCTCTAGCAGGAGCAATTAACTTTCTAGTAACAATCTTCCAAGCTCGATCCCCCGGCATGACTCTTGAACGAATTCCTCTTTACCCATGAGCAGTCGCAGTCACAGCACTACTTTTAGTCTTAGCCCTCCCAGTCTTAGCAGGAGCAATTACCATGCTCCTAACTGATCGTAACTTTAACACATCATTCTTTTCACCTGACGGAGGAGGAGATATCATCTTATTCCAACACCTCTTC-

>x6_G6YZTIE01B8ZGS

--CCTTAGAAGGAATCTGGCCCATGCCGGCGCTTCCGTTGATTTTGCTATCTTCTCCTTACACCTAGCGGGAGTTTCATCTCTTTTAGGAGCTGTAAATTTTATTAGGACTCTGGTGAATTTGCGCGTGATAGGGATAGTGATGGATCGGATACCCCTGTTTGCTTGAGCTGTGTTTGTGACAGCAATCTTGCTCCTTCTGTCCCTGCCCGTGCTGGCTGGGGCTATTACCATGTTACTTACCGATCGAAATCTTAACACTTCTTTCTATGACCCAAGTGGGGAAGGGGACCCGATTTTGTACCAACATCTGTTT-

>x7_G6YZTIE01BFBA5

--CCTAAGTTCTAATTTAGCACACAGGGGTGCAGCTGTGGATTTGGCCATTTTTTCATTACACTTAGCCGGTATCTCGTCTCTGTTAGGGGCCGTAAATTTTATCACCACCCTGGTCAACTTGCGGTCTTTGGGGATGTTGGTAGAGCGCATACCCTTATTTGCCTGGTCCGTTCTTGTGACTGCTGTTTTATTGTTACTGTCTTTGCCTGTCTTAGCAGGGGCCATCACTATGCTCTTGACAGATCGAAACTTGAACACTAGATTTTACGACTCCAGGGGAGGAGGGGACCCTGTTCTATACCAGCACTTGTTC-

>x8_G6YZTIE02DGD5P

---CTCAGTTCAAACGTAGCACACTCAGGCCCCTCGGTAGACCTTGCGATTTTTGCTCTCCATTTAGCGGGG---TCCTCTTTATTGGGTGCCGTTAATTTCATTAGAACATTTTCTAATTTGCGTACGTTAGGGATATTACTAGAACGTGTTCCTCTTTTTGGGTGAGCTGTCTTAATTACAACAATCTTGCTTCTTCTCTCCCTGCCCGTATTGGCAGGGGCAATTACCATGTTACTAACAGATCGAAACTTAAACACCTCATTTATTGATTCCAATGGAGGGGGTG---------------------------

>x9_G6YZTIE01CGN1B

--TCTTTCTGGGGCGCTGGCCCATGCTGGGGCTTCTGTGGACTTGGCTATCTTTTCATTACATCTTGCTGGAGTAAGCTCTATTCTGGGGTCTTTGAATTTTATTACAACAGTCATTAATATACGTCCGGAAGGGATAACGTGGGAGCGGGTGCCGTTGTTTGTTTGAGCGGCTTTCATTACTGTGATCTTGTTATTATTAGCTTTGCCGGTGTTAGCTGGGGCTATTCCTATGTTATTAACTGATCGGAATTTAAATACTGCTTTCTTTGATCCTAGGGGAGGAGGGGGATCCAATTTTTATTCTCCCATTTAT-

>x10_G6YZTIE01B4RX0

--CCTAAGCGGAAACCTTTCCCACTCAGGAGCTTCCGTAGACTACGCAATTTTCTCTCTTCACTTAGCAGGAGTTTCATCTTTGTTAGGAGCTGTGAATTTTATTAGCACTCTTAGAAATCTTCGAGTTTTTGGGATAATACTAGACCGTTTACCTCTATTCGCTTGAGCAGTTTTAGTTACTGCTATTTTACTTCTTCTTTCTTTACCAGTATTAGCTGGGGCTATCACTATGCTGCTCACTGACCGAAATTTTAATACGTCTTTTTACGACCCAAGAGGGGGAGGAGACCCACTTCTTTACCAGCACTTATTT-

>x11_G6YZTIE01BB8AP

---CTATCCTCTAGCCTCGCCCACGCAGGCCCTTCTGTTGACCTAGCTATCTTCTCCCTCCACTTAGCAGGGGCGTCCTCTATTCTAGGCTCCATTAATTTCATTTCAACTATTATAACTGCACGACAAGAAGCCTACACCCTAGTTCGCATGCCCCTCTTTATCTGAGCAGTAAAAATTACAACCATCCTCCTCCTTCTATCTCTGCCCGTGCTAGCTGGCGCCATTACTATGCTTCTCACCGACCGTAATGCCAACACATCCTTCTTCGACCCAAGAGGAGGTGGGGACCCAGTCCTATTCCAGCACCTT----

>x12_G6YZTIE02D61N7

--ATTATCCCACTCTATTGCCCACGCAGGACCAGCAGTCGACATAACAATTTTCGCCCTACACATAGCAGGCGCATCTTCTATTATAGGCGCCATTAACTTTATATGTACAGTAATTAATATACGACCA---AATATACCAATCACATGTATACCTCTATTTGCCTGATCAATATTATTAACAGCCATTCTTCTCCTACTATCCCTACCTGTATTAGCAGGGGCTATCACCATACTTTTAACTGACCGTAATATAAACACCTCTTTCTTCGACCCAGTAGGGGGAGGGGACCCAATCCTCTACCAACATCTATTC-

>x13_G6YZTIE01CCRYS

--CCTGTCAGCTAACCTGGCCCATGCCGGACCATCAGTAGACCTAGCTATTTTCTCCCTTCATCTAGCCGGTGTCTCTTCTATTCTTGGCGCCCTTAATTTTATCTCCACCATCCTGAACATGCGCCCCTCAGCGCTAGCCCTAGAAAAAATCTCCCTCTTCGTCTGAGCAGTTATAATTACTGCTATTCTTCTCCTTTTATCTCTCCCCGTCTTAGCAGGAGCTATTACCATACTCCTAACTGACCGCAACCTAAATACCTCCTTCTTCGACCCAGCTGGAGGGGGCGACCCTATTTTGTATCAACACCTCTTC-

>x14_G6YZTIE01BL5P1

--TCTAGCTAGCATTCAGGCTCACTCTGGAGGTTCTGTGGACATGGCCATATTCAGTCTACATTTGGCTGGTATCTCGTCTATCTTAGGCGCCATCAACTTCATAACTACTATCTTTAACATGAGAGCTCCTGGCATCTTGTTCGATCGGATGCCTCTCTTTGTCTGATTTATTTTGGTCACTGTCTTCTTACTTCTCCTATCTCTGCCCGTTTTGGCTGGTGCCATTACGATGCTTCTCACTGACAGAAACTTCAATACGACCTTCTTTGATCCGGCAGGTGGTGGCGATCCGGTTTTGTATCAGCACTTATTT-

>x15_G6YZTIE02DUT2E

--TTTAGCAGGAAATATCGCGCATGCGGGTCCTTCAGTTGATCTTGCTATTTTTTCACTTCATATTGCAGGAGTCTCCTCAATTTTAGGAGCTCTTAACTTCATCACAACTGTTATCAATATACGATATAAAGGATTACGCCTAGAACGCGTGCCTCTATTCGTATGAGCCGCTAAAGTTACAGCCATTCTTCTTCTCTTATCATTACCTGTGCTAGCTGGAGCCATTACAATACTATTAACAGATCGGAATTTGAATACAGCTTTTTTTGACCCCGCTGGAGGAGGTGACCCTGTTCTTTACCAGCATCTATTC-

>x16_G6YZTIE02EDD34

--TTTATCTAGAAACTTAGCCCACGCAGGACCCTCAGTGGATCTGGCTATCTTTTCGCTTCATTTAGCCGGAGTGTCTTCAATTCTAGCCTCTTTAAACTTTATTACCACTACTATCAACATGCGGTCAAAAGGAATACGGATAGAGCGAGTACCTTTATTTGTTTGATCCGTGATGATTACGGCAATCCTTCTACTTCTTTCCTTACCAGTCTTAGCTGGAGCCATTACTATGCTTCTAACGGACCGGAACTTAAATACTGCTTTCTTCG---------------------------------------------

>x17_G6YZTIE02DUG09

--TTTAAGAAGATATGCTGCTCACTCAGGAAGTTCAGTTGATTTTGCT---TTTTCTCTTCATTTAGCTGGTGTGTCATCTTTATTAGGGGCTGTTAATTTTATTACTACAATTTTGAATATGCGTGTACCTGGACTTTTACTTGATCGGGTACCATTGTTTGTTTGATCAATTTTATTTACAGCCATCTTGCTTTTATTATCGTTACCTGTTTTAGCAGGAGCAATTACTATATTGTTAACAGATCGAAACTTAAATACTGTATTTTATGATGTTAGAGGAGGAGGGGATCCTATTTTATATCAACATCTGTTT-

>x18_G6YZTIE01BOROC

----TGTCAAATGCAGTTTACCATAGCGGGCCCTCTGTAGACCTGTCTATCTTTTCTCTTCACTTAGCAGGTGCCTCCTCTATTCTTGGTGCTTCAAATTTTATTACAACCCTTATAAATATAAAA------GGAGATAAGATTGACTATTTGAATCTTTTTTCTTGAACGATCTTAACAACCGTTTTCCTTTTACTCCTCTCCCTTCCTGTCCTCGCTGGGGCGATCACAATACTCCTCACAGATCGGAACTTAAACTCTACCTTTTTTGAAGTTAGAGGAGGAGGGGATCCTGTTCTCTACCAGCATTTATTT-

>x19_G6YZTIE02EEET0

---CTTTCTGGGGTATTAGCTCATGCGGGCCCTGCAGTAGACTTCGCAATTTTTTCTCTTCATTTGGCCGGAGCGAGGTCAATTTTGGGCGCTTTAAATTTTATTACTACCGTAATTAATATGCGAAATAAGGAGTTAAGGTGAGAACGAGTACCTTTATTTATTTGAGCGGTATATATTACAGTTATTATGTTACTTTTAGCGCTTCCTGTACTGGCAGGTGCTATTACTATATTACTTACAGATCGAAATCTTAATACTTCCTTCTTTGACCCAAGAGGGGGAGGAGATCCTGTATTATTTTCTCATCTTTTT-

>x20_G6YZTIE02EELX4

--CTTAAGCCACAGAGTAGCCCACTCTTCCCCCTCTGTCGATTTCACTATTTTTTCCCTCCATTTAGCTGGAGTTTCGTCTTTATTAGGTGCGGTAAATTTTATTAGAACTCTCATAAACCTCCGAGTTATGGGAATAGTCCTGGAGATAACCCCTTTGTTCGCTTGAGCTGTGTTAATCACCGCCGTACTTCTTCTTCTTTCTCTCCCTGTTTTGGCAGGAGCTATCACTATACTACTAACGGACCGAAATTTAAATACGAGCTTTTATGACCCGGCCGGAGGGGGG---CCTATCCTCTATCAGCATTTATTT-

>x21_G6YZTIE01BEGFQ

--TTTATCTGCTGGAATCGCCCACGCGGGGGCCTCTGTTGACCTGGGTATTTTTTCACTTCACCTGGCTGGGATCTCTTCGATCCTGGGAGCTGCTAATTTCATTTCTACAGTAGTCAACATACGAAGAACAGGCATACAGATAGATCGAATACCCCTGTTTGTATGGTCGGTTTTCCTGACCGCTATTCTCCTACTCCTGTCACTACCCGTACTAGCAGGAGCAATCACTATGTTACTTACAGATCGTAATCTCAATACTTCGTTTTTCGACCCAGTAGGAGGAGGAGATCCTATCTTATACCAACACCTTTTC-

>x22_G6YZTIE01A1MMC

--ATTATCTAGCGGCCTAGCCCACGCCGGAGGATCAGTGGACCTTGCAATATTCTCACTCCACCTTGCAGGAGCATCCTCTATCCTGGCCTCCATAAAATTCATCACTACTGTAATAAACATGCGAACCCCAGGAATCTCGTTCGACCGTCTGCCGCTATTCGTCTGATCAGTATTCGTAACAGCATTTCTGCTACTCCTTTCCCTTCCCGTTCTAGCTGGAGCTATAACAATGCTTCTCACCAATCGAAACGTAAACACAACCTTCTTTGACCCCGCGGGGGGAGGAGACCCTATTTTATTCCAGCACCTTTT--

>x23_G6YZTIE02DVFS8

--AGTATCTAGAAACATTGCCCATGCTGGCCCATCCGTCGACTTAGCAATTTTTTCCCTCCATTTAGCCGGAGTATCTTCTATTCTAGGTTCAATTAACTCTATCACTACTGCATCCAATATGCGATGAGAAGGTATATTAGCAGAGCGCATACCCCTCTTTGCCTGAGGTGTAGTAATCACTACAGTCCTTCTCCTTCTCTCCCTTCCCGTCCTAGCCGGAGCAATCACCATACTTTTAACAGACCGAAATCTAAATACATCCTTTTTTGACCCTGCGGGAGGCGGCGACCCCGTCCTCTACATACACCTATTT-

>x24_G6YZTIE01BTJJM

--TCTTAGAAGCAATATCGCCCATTCGGGGCCCTCAGTTGATTTTGCCATTTTCAGGCTTCACCTTGCAGGGGTTTCGTCTCTTTTAGGGGCCGTAAATTTTATTAGAACGCTGAGAAACCTACGAACGCTCGGAATACTTCTCGATCGTATACCCCTCTTTGCTTGGGCTGTCTTAATTACCGCCATTCTTCTTCTTCTGTCCCTCCCTGTCTTAGCGGGGGCCATCACTATACTTCTAACGGATCGAAACCTAAACACCTCCTTCTATGACCCTAGAGGGGGAGGGGACCCAGTTCTTTACCAACATCTCTTC-

>x25_G6YZTIE01BVDJW

--TTTGGCCGGTAACTCCGCTCACGGGGGCGGGGCTGTTGATCTGGCTATTTTTTCTTTACATCTAGCAGGAGCCTCCTCCATTTTGGGAGCTATTAATTTTATTTCTACTGTATTGAACATGCGAAGCCCTTATATAAGGATAGACCAGATGCCTTTGTTTGTTTGATCAATTTTTATCACTACAATTCTACTTTTATTATCTTTACCTGTGCTGGCGGGAGCTATTACGATATTGTTGACAGACCGGAATTTAAACACCTCTTTTTTTGACCCAACTGGAGGTGGAGACCCTATTCTATACCAACATTTGTTT-

>x26_G6YZTIE01A0ZTB

--GTTAAGTTCAAATATCTCTCACAGCGGAATATCTGTAGATTTCGCAATTTTTTCTCTACACTTGGCGGGAATTTCTTCCCTGTTAGGGGCCGTGAATTTTATTAGGACTTTGGGTAACTTACGCACTATAAATATAACTTGGGAAAAAATACCCCTTTTTCCCTGGGCTGTGCTAATCACGGCAGTTTTGCTTTTACTCTCTTTACCTGTCTTAGCCGGAGCCATTACTATGCTTTTAACGGACCGCAATTTAAATACTTCGTTTTATGATGTTGCAGGGGGCGGTGACCCGGTGCTTTACCAACACTTATTT-

>x27_G6YZTIE02D53O3

--TTTAAGAAGAAACGTGTCACATGCAGGTGCATCTGTTGACTTTGCTATTTTTTCATTACACCTGGCGGGTGTTTCGTCGCTATTGGGCGCAGTTAATTTCATTACCACTTTAAGTAATTTGCGTGTTATAGGAATGTTAGTTGATCGAATGCCACTGTTCCCTTGAGCAGTGCTGGTTACTGCGATTCTTCTCTTGCTTTCACTTCCTGTCTTAGCTGGGGCTATTACAATACTCTTAACAGACCGAAACTTTAACACTTCCTTCTATGACCCTAGGGGAGGCGGGGACCCTTTACTTTACCAGCATTTGTTT-

>x28_G6YZTIE01BXNQX

--TTTGAGATCTACTCTAGGCCACTCAGGCGCCTCGGTTGATTTTGCAATTTTTTCGCTGCACCTCGCAGGGATTTCGTCCTTATTGGGTGCAGTAAACTTTATTAGCACACTAAAAAATACCCGAACATTCGGCTTGGTTTTAGACCGAATGAGTCTTTTCCCACGATCAGTCTTAGTGACTGCAATCTTGCTTTTGCTCTCTCTCCCCGTTTTAGCAGGAGCAATTACCATACTTCTAACTGACCGAAATTTGAACACAACTTTTTATGACCCGAGGGGCGGTGGGGACCCTGTTCTCTATCAGCATCTTTTT-

>x29_G6YZTIE02C6U1X

--ATTAAGAAGAAATTTAGCACATGCTGGAGCGTCTGTGGATTTTGCTATTTTTTCTCTACACTTAGCTGGTATTTCTTCTCTATTAGGAGCCGTAAATTTTATTAGGACGTTAGGGAATTTGCGGGCTTATGGTATAGGTTTAGACAATGTGCCCCTGTTTGCTTGAGCTGTATTTATTACAGCTGTTTTGCTTTTGCTCTCTCTGCCTGTTTTAGCGGGGGCCATTACCATATTGCTGACCGATCGTAACTTAAATACTGCTTTTTATGATGTAGCAGGGGGAGGGGATCCAGTTCTCTATCAGCATTTATTT-

>x30_G6YZTIE02ESMAT

--CCTATCAGCGAGAATTGCCCACGCAGGTGCATCCGTGGACCTGGGGATTTTCTCGCTTCATTTGGCCGGGGTTTCATCAATCCTAGGAGCAGTAAATTTCATAAGAACTGTCCTAAACATGCGAGCAACAGGAATAACAATAGACCGCATGCCCCTGTTTGTATGATCTGTATTCCTCACAGCCATCCTCCTTCTCCTGTCCCTCCCAGTCCTAGCTGGAGCTATTACCATGCTACTCACAGACCGTGACATCAACACATCCTTCTTTGATCCTGCCGGAGGGGGTGATCCCATCCTGTATCAACACCTATT--

>x31_G6YZTIE02D5WK2

--ATTAGCAGGGCCTCAGACACATTCTGGGGGATCTGTAGATATGGCAATATTTAGTTTACACTGTGCAGGTGCATCATCTATTATGGGTGCCATAAATTTTATTACTACCATATTTAATATGAGAGCCCCTGGGTTAACAATGGATAAATTACCTCTTTTTGTTTGATCAGTTTTGATTACAGCATTTTTATTATTACTTTCTCTACCTGTCTTAGCTGGAGCTATTACGATGTTATTAACAGATAGAAATTTTAACACAACCTTTTTCGACCCGGCAGGAGGGGGTGATCCAGTTTTATACCAACATTTATTT-

>x32_G6YZTIE02EQTTS

---CTAGCTTCCAACATCGCCCATGCTGGTCCTTCTGTCGACCTTGCCATTTTCTCCCTTCATTTAGCAGGAATTAGTTCAATCCTAGGAGCCCTAAACTTTATTACCACAATTTTTAATATACGCTCTAAGGGCCTACGCCTAGAACGAATACCTTTATTTCTCTGAGCCGTAAAAATTACAGCTATTCTCCTTCTTCTGTCCCTTCCTGTTCTCGCCGGAGCAATCACAATACTTTTAACAGATCGTAACCTGAACACAGCTTTCTTTGACCCCGCTGGAGGGGGAGACCCGGTACTCTACCAACACTTATTC-

>x33_G6YZTIE01CBSA8

--ACTGGCTGGTAATACTGCGCATAGTGGAAGATCAGTAGATTTAGCTATTTTTTCGCTTCATTTGGCTGGTGCTTCGTCTATTCTGGGTGCTATTAATTTTATTACTACAGTAATAAATGTACGACCAAGATACATATCTATAGACCTAGTACCTTTATTTGTGTGATCTGTGTTTATTACTGCGATCTTACTTTTATTATCATTACCAGTACTAGCAGGAGCTATTACTATATTATTAACAGATCGTAATTTAAACACTTCTTTTTTTGACCCCATAGGAGGTGGAGACCCAATTCTTTATCAACACTTGTTC-

>x34_G6YZTIE02EVV0M

--TTTATCAGGGATTCAGGCTCATTCTGGGGGTTCAGTAGATTTAGTAATTTTTAGTTTACATTTGGCAGGAATTTCATCTATATTAGGTGCAATGAATTTTATTACAACAATAATAAATATGAGAGCGCCGGGAATAACTTTTGATAGAATGCCATTATTTGCTTGATCTATTTTAGTAACTGCAGTTTTATTATTATTGTCTTTACCGGTATTAGCTGGGGCTATAACTATGTTATTAACAGATAGAAATTTTAATACTGCATTTTTTGACCCTGCTGGGGGAGGAGACCCGATTTTATATCAACATTTATTT-

>x35_G6YZTIE02EEC3F

--TCTTGCAAGCAATATCGCCCATGCTGGCCCTTCAGTTGATCTAGCAATTTTTTCACTCCACTTAGCAGGCGTCTCGTCTATCATAGGAGCATTAAATTTTATTACCACAGTGATTAATATGCGTTCAAAAGGACTTAAACTAGAACGTGTTCCTTTATTTGTTTGATCTGTAGTAATTACTGCTGTGCTATTACTACTTAGCCTTCCTGTATTAGCCGGAGCTATTACTATACTTTTAACCGATCGAAACCTCAACACTGCCTTTTTTGACCCAGCTGGCGGGGGCGACCCTATTTTATATCAACATTTATTC-

>x36_G6YZTIE01CAW9L

--CTTAGCTTCTAACATCGCTCATGCTGGCCCTTCGGTTGATTTAGCTATCTTTTCTCTTCATCTAGCTGGGATTAGATCAATCCTCGGAGCCCTAAACTTCATTACTACAATTTTTAATATACGATCTAAAGGACTCCGTCTAGAACGCATGCCTCTCTTCCTATGAGCTGTAAAAATCACAGCCATCCTCCTTCTTCTGTCTCTCCCTGTCCTCGCCGGAGCTATTACTATACTCCTAACTGACCGTAACTTAAATACAGCCTTCTTTGACCCTGCCGGGGGTGGGGACCCTGTTCTATATCAACATTTATTT-

>x37_G6YZTIE02C4A66

---CTTTCAAGAAATATTGCTCACAGTGGCCCACCAGTAGATATGGCCATCTTTTCCCTCCACTTAGCAGGGGCCAGATCAATCCTGGGGGCCGCCAACTTCATCGCCACATTAATCAACATACGTGTAAAATCACAAAAATCTGACAAAATACCGCTATTCACTTGATCAGTTATACTTACAGCCATCCTCCTTCTCCTATCTCTTCCTGTACTTGCTGGCGCAATTACAATACTACTTACAGACCGAAATTTAAACACAACATTTTTTGATAGGAGAGGGGGGGGTGACCCCATTCTTTATCAACATCTATTC-

>x38_G6YZTIE02EVOYJ

--CCTTGCAAGAGGAATTGCCCACGCAGGAGCCTCAGTTGATCTTGGAATCTTCAGGCTACACTTAGCGGGAGTCTCCTCCATCCTAGGAGCAGTTAACTTCATAACCACAGTTATTAATATGCGAACAACAGGAATAACTATAGACCGTATACCCCTATTTGTCTGGGCAGTATTTCTAACAGCCATCCTACTTCTTCTAAGACTACCTGTTCTTGCTGGAGCAATCATCATACTACTAACAGATCGAAATTTAAACAC--------------------------------------------------------

>x39_G6YZTIE01BYQ49

---TTATCAAGAAACCTCGCTCATGCAGGCCCCTCAGTCGACATAGCAATCTTCTCGCTCCACCTCGCAGGAATTTCATCAATCCTAGGATCAATTAACTTCATTACGACAGCCTTTAATATACGTATCAAAGAACTACACCCAGAAAAAACACCCCTATTCGTATGAGCTGCTGCAATCACCACCGTCCTTCTCCTTCTCTCCCTGCCCGTCCTGGCAGGAGCTATCACGATACTCCTAACCGACCGTAACCTCAACACCTCCTTCTTCGACCCCTCAGGAGGAGGAGACCCAATCCTTTTCCAACACCTA----

>x40_G6YZTIE01CAZW0

--ACTATCTAGCAACCTTGCTCATGCTGGGGCCTCCGTTGATCTGACTATTTTCTCCCTACATTTAGCAGGTGTGTCCTCTATTTTAGGTGCTATTAACTTTATTACTACTATTACTAACATGAAGCCCCCGGCTCTTACGCAATATCAAACACCATTATTTGTTTGAGCCGTCCTAATTACCGCCGTTCTTTTACTCCTTTCGCTCCCTGTCTTAGCCGCCGGCATCACAATGCTGCTAACCGATCGAAACCTTAACACCACTTTCTTCGACCCTGCAGGCGGAGGAGACCCAATCCTGTACCAACACC------

>x41_G6YZTIE01BZHN0

--CTTGGCTTCTGCTATTGCTCATGCGGGCGCTTCTGTTGATATGGGTATCTTTTCTCTTCACTTGGCTGGGGTGTCTTCTATTTTGGGTGCTGTAAATTTTATGACAACTGTTATGAATATACGGGCTTCCGGGATAACTATGGATCGCATGCCTCTTTTTGTATGGTCTGTTTTTATTACTGCTATTCTCCTGCTGTTGTCTCTGCCTGTGCTAGCCGGCGCTATTACTATGTTGTTGACGGATCGTAATCTGAATACTTCTTTTTTCGACCCCGCTGGCGGCGGAGATCCTGTTCTATACCAGCATTTATTT-

>x42_G6YZTIE01BH0Z7

--ACTGTCCACTAAAATCGCCCACTCAGGGCAATCAGTTGATTTTACAATTTTCTCACTCCACCTAGCAGGTCTATCCTCACTTATAGGAGCTATTAACTTTATAACAACAGTGATTAATATACGTCCAAAAGGAATAGTAATAAGACGTATGCCATTATTTGTTTGAGCCCTATTTTTAACAGCTATTCTTCTCCTTCTCTCCTTACCAGTATTAGCAGGCGCTATTACTATACTCTTAACCGACCGTAATATTAATACCTCCTTTTTCGACCCGGTTGGAGGTGGAGATCCAATTTTATACCAACACCTATTT-

>x43_G6YZTIE01BNDJR

--CTTAGCTAGAGGAATCGCCCACGCTGGAGCTTCAGTTGATATAGGAATCTTCTCTCTTCACTTAGCTGGAGTTTCTTCCATTCTCGGAGCAGTTAATTTTATAACAACCGTAATTAACATACGAAGAGAGGGAATAATAATGGACCGCATGCCACTATTTGTCTGATCAGTCTTCTTAACAGCTATTTTACTCCTTCTCTCGCTCCCTGTTCTAGCCGGAGCTATCACTATATTGCTAACAGATCGAAATTTAAATACTTCTTTTTTCGATCCCGCAGGAGGGGGTGATCCTATTCTCTATCAACATTTATTT-

>x44_G6YZTIE02DOBZB

--CCTATCTGCAAATATTGCTCATGCAGGGGCCTCAGTAGACCTTGGTATTTTTTCCCTCCATTTAGCAGGAGTCTCATCTATCCTAGGAGCTGTAAACTTCATCTCTACAATTCTTAATATACGAAGAACTGGAATAAATATGGACCGAATCCCTCTGTTTGTCTGATCTGTATTATTAACTGCTATTTTATTACTCCTATCTCTCCCAGTCCTAGCAGGTGCAATTACTATACTTTTAACTGACCGAAACTTGAACACTTCATTT---GACCCTGTTGGAGGCGGAGACCCAATCCTGTATCAGCATTTATTT-

>x45_G6YZTIE01A7KST

--TCTATCCGCAGGAATTGCCCACGCTGGCGCTTCGGTAGATCTTGGGATTTTTTCACTTCACTTAGCAGGAGTATCTTCTATTCTAGGTGCGGTAAACTTCATTTCTACTGTGATTAACATACGAAGAACAGGAATAAACATAGACCGTATGCCTCTTTTTGTGTGATCTGTATTCCTGACAGCTATTTTACTTTTACTCTCTCTTCCAGTTCTCGCCGGAGCGATCACGATGCTATTGACTGACCGGAACCTAAACACATCTTTTTTTGATCCTGTGGGAGGAGGAGACCCTACTCTATATCAACACTTATTC-

>x46_G6YZTIE01BUT1S

--TCTCTCTGCCGGTATTGCTCATAGAGGTCCTTCAGTTGATCTTGGTATTTTCTCGCTTCATTTGGCAGGAATCTCTTCAATTCTAGGTGCGGTAAACTTCTTAACAACCGTTATTAACATACGAGCTCCCGGCATATCGATGGACCAACTACCATTATTTGTCTGAGCTGTCTTCATCACTGCTCTTTTGCTACTTCTTTCATTACCTGTTCTTGCCGGAGCTATTACTATGCTTCTTACGGAC---AATATTAAC----------------------------------------------------------

>x47_G6YZTIE02EPCFM

--TTTATCAGGACCTCAGATGCATTCTGGGGGTTCAGTTGATATGGCTATTTTCAGTCTTCATTGTGCAGGAGCCTCATCGATTATGGGAGCAATAAATTTTATAACAACCATTTTTAATATGAGGGCACCAGGATTAACTATGGATAAAATGCCCCTATTTGTTTGAGCTGTATTAATAACAGCTATATTATTACTTTTATCCTTACCAGTTTTAGCTGGGGCTATAACAATGCTATTAACTGATAGAAATTTTAACACTACTTTCTTTGATCCTGCAGGAGGAGGGGATCCGATATTATATCAACATTTATTT-

>x48_G6YZTIE01BBBAR

--CTTATCAACCAACATCGCCCACGCAGGAGCCTCAGTAGACCTAGGAATTTTCTCCCTACACCTAGCTGGTGTGTCTTCCATCCTGGGAGCCATTAATTTTATAACCACAGTTCTAAACATACGATCCACAGGAATAACTATAGACCAAATACCCCTATTTGTGTGAGCTGTCTTCCTCACAGCCATCCTACTTCTCCTCTCTCTTCCAGTGCTAGCGGGAGCAATTACGATACTTCTTACAGACCGAAACCTTAATACATCATTTTTCGACCCCACAGGAGGAGGTGATCCAATCCTTTACCAACACCTATTC-

>x49_G6YZTIE01A7FGP

--GTTAGCCGGAAATCTAGCACATGCTGGCGCTTCCGTAGATTTAACAATTTTTTCCCTTCACCTCGCAGGGATCTCGTCAATTCTAGGGGCCATTAACTTTATTACCACAATTCTTAACATGAAGCCTCCAGCCATCTCACAATACCAAACGCCCCTCTTTGTCTGAGCTGTATTAATTACAGCCGTGTTTCTACTTCTGTCCCTGCCAGTTCTAGCTGCCGGAATTACAATACTCTTAACGGATTGAAATTTAAACACAACCTTCTTCGACCCTGCCGGAGGGGGAGACCCAATCTTATACCAACACCTG----

>x50_G6YZTIE01B1JP0

--GCTTGCTGGTAACCTAGCACATGCAGGGGCCTCCGTCGATCTAACCATTTTCTCCCTACATTTGGCCGGGATTTCCTCAATTTTAGGGGCAATTAACTTCATCACTACAATTCTTAATATGAAACCCCCCGCCACCTCGCAATATCAAACACCCCTATTTGTTTGAGCAGTACTAATTACAGCCGTCCTCCTACTCCTCTCCCTGCCAGTTCTTGCTGCCGGTATCACAATGCTTCTCACAGACCGAAATCTAAATACAACCTTCTTTGACCCCGCAGGGGGAGGAGACCCCATCCTCTACCAACATTTA----

>x51_G6YZTIE02C3L47

---TTGGCTGCCGCTATTGCCCATGCAGGTGCTTCAGTAGATCTAGGTATCTTCTCACTCCACCTAGCTGGTGTTTCTTCGATCCTAGGAGCTGTGAACTTTATAACTACTGTTATTAATATACGATCATTTGGTATAACAATAGACCAAATACCTTTATTTGTTTGAGCCGTCTTTATTACTGCTATCTTACTCCTTCTCTCACTTCCTGTGCTAGCAGGGGCCATCACGATACTTTCGACTGATCGTAATCTAAATACCTCCTTCTTTGACCCTGCTGGTGGTGGTGATCCTATTCTATATCAGCACTTGTTC-

>x52_G6YZTIE01BTU44

--ATTAGCTAGAAACATCGCTCACTCAGGAGCCTCAGTAGATCTAACAATCTTTTCTTTACATTTAGCAGGAGCCTCATCCATTTTAGGAGCTATTAATTTTATATCTACAGTAATTAATATACGAGGGAAAAGACTAACCTTCGACCGTCTACCTTTATTTGTGTGAAGAGTGTTTGTAACTGTAATTTTATTACTATTATCACTTCCAGTTTTAGCAGGTGCTATCACCATACTACTTACTGACCGAAACTTAAATACATCGTTTTTTGATCCTACTGGGGGAGGAGATCCTATTTTATACCAACATTTATTC-

>x53_G6YZTIE02C8MY5

--TTTAGCAACTGTAATCGCCCACGCCGGAGCTTCAGTTAATATAGGCATCATTTCCCTTCACCTCGCGGGCGTATCCTCAATTTTAGGAGCCGTAAATTTTATGACCACGGTTATTAATATGCACTCATTTGGGATAACTATGGACCAAATACCATTGCTTGTTTGAGCTTTATTTATTACAGTCATTTTACTTCTCCTGTCCCTACCTGTTTTATCAGGTGCCATCACTATACTCCTGACCGATCGAAATCTAAACACCTCATTCTTTGACCCAGCCGGCGGTGGAAACCCTGTTTTATACCAACACTTATTC-

>x54_G6YZTIE01AX4RX

--ACTCTCTGGCAACTTAGCCCACGCAGGGGCTTCCGTTGACCTGACTATTTTTTCACTTCACCTAGCAGGGATCTCGTCCATCCTAGGTGCAATTAACTTCATTACTACAATTATTAATATGAAACCGCCCGCTATTTCCCAATACCAAACCCCACTCTTTGTGTGAGCCGTACTAATCACGGCCGTCCTACTACTCCTCTCTCTTCCAGTACTGGCAGCCGGAATTACTATGCTTCTGACGGACCGGAACCTAAACACCACTTTCTTTGACCCTGCAGGAGGAGGAGGTCCCATTCTTTACCAACATCTA----

>x55_G6YZTIE01A4RNQ

TAT---GCGGCTGCAATCGCCCACGCCGGTGCCTCAGTTGATATGGGTATTTTTTCACTCCACTTAGCAGGGGTGTCCTCAATTTTAGGAGCCGTAAACTTTATAACCACAGTTATTAATATGCGCTCATTCGGAATAACTATAGACCAAATACCACTATTTGTTTGATCCGTATTTATTACAGCAATTCTCCTCCTTCTATCACTTCCCGTGTTAGCCGGTGCTATTACTATACTTCTAACTGATCGAAACCTAAACACATCATTCTTCGATCCAGCTGGCGGTGGAGACCCTGTCCTATATCAACACTTATTC-

>x56_G6YZTIE01BLCF0

--ATTAGCCTCAGCAATCGCACACGCTGGTGCATCAGTTGACCTTGGAATTTTTTCACTCCAT---GCAGGGGTGTCATCTATTTTAGGAGCAGTTAACTTTATAACTACTGTTATCAACATACGATCATATGGTATAACAGCTGACCAAATACCACTATTTGTTTGAGCTGTCTTTATTACTGCTATCTTACTACTTTTATCCTTACCAGTGTTAGCGGGAGCGATCACTATGCTTCTAACAGACCGAAATTTAAACACATCATTCTTCGACCCTGCAGGAGGAGGAGACCCCATCCTATATCAACACTTATTT-

>x57_G6YZTIE01CCTUM

--TTTGTCTGCTGCTATTGCTCATGCTGGTGCATCTGTTGATTTGGGAATCTTTTCTCTTCATTTAGCAGGAGTGTCATCTATTTTAGGAGCTATTAATTTTATAACTACTGTGATTAATATACGTCCTCAGGGTATAAGGATAGACCGAATGCCTTTATTTGTGTGGTCGGTTTTTATTACTGCTATTTTATTACTACTTTCTTTGCCGGTTCTAGCAGGAGCCATTACTATGCTGTTAACAGATCGTAATTTGAATACGTCGTTCTTTGACCCGGCTGGA---GGAGATCCTGTTTTATATCAGCATTTATTT-

>x58_G6YZTIE01A2HET

--CCTATCAGCAGGGATCGCCCACGCAGGAGCTTCAGTAGACCTGGGAATTTTCTCTCTTCACCTGGCAGGAATCTCATCAATTCTAGGAGCCGTTAATTTTATAACTACAGTCATTAACATACGGACTACAGGAATAACAATAGACCGAATACCTCTATTCGTTTGAGCCGTTTTCCTAACAGCGATCCTGCTACTTCTAAGGCTACCAGTCCTTGCCGGGGCTATTACCATACTACTTACAGATCGAAACTTAAATACAGCCTTCTTTGATCCGGCAGGTGGAGGAGATCCAATCCTATACCAACATCTA----

>x59_G6YZTIE01A7EOJ

--TTTGGCAGGCAATCTGGCACATGCTGGAGCCTCCGTCGATCTAACCATCTTTTCTCTTCATCTTGCAGGCATTTCCTCTATCCTAGGGGCAATCAACTTCATTACTACTATTATTAATATAAAACCTCCTACTATCTCTCAGTATCAAACACCCCTATTCGTCTGGGCCGTACTTATTACAGCTGTGCTATTGCTTCTTTCCCTTCCCGTCCTCGCCGCCGGAATCACAATACTTCTCACAGACCGAAACCTCAATACAACCTTTTTTGACCCTGCAGGCGGGGGAGATCCCATTCTCTACCAACATCTA----

>x60_G6YZTIE01BRS32

--CTTACCTGCCGGAATTGCTCATGCCGGTGCTTCCGTTGATATAGGAATCTTTTCTTTACACTTAGCAGGTGCTTCTTCGATTCTAGGGGCTGTAAACTTTATTACGACTGTAATTAACATGCGCTCGAGTGGAATAACTATAGACCGTATACCTCTTTTTGTTTGAGCTGTCTTTATTACAGCTATCTTGCTTTTATTATCACTCCCGGTACTAGCTGGAGCTATTACTATGCTTTTAACTGACCGTAATTTAAATACTTCCTTCTTCGATCCTGCGGGAGGCGGAGACCCTGTTCTATATCAACACTTATT--

>x61_G6YZTIE01A1W0K

--CCTCTCAGGCGGTATCGCCCACGCTGGAGCTTCCGTGGACATGGGTATTTTCTCCCTTCACCTAGCCGGTGTCTCTTCCATCCTAGGGGCCGTTAACTTCATTACCACCGTAATCAACATACGATCCCCGGGAATGACTATGGACCGAATCCCACTATTCGTCTGAGCTGTATTCCTTACAGCCATCCTCCTCCTCCTATCTCTGCCCGTCCTAGCCGGGGCAATCACTATACTTCTAACAGACCGAAACTTAAACACCTCATTCTTTGACCCCGCTGGAGGAGGGGACCCCATTCTATATCAGCACCTATTC-

>x62_G6YZTIE02D7HIO

-CTCTTGCTGGAAACCTA---CACGCTGGCCCTTCAGTCGACTTAACGATCTTTTCCCTACATTTAGCAGGCATTTCTTCTATTTTAGGAGCTATTAACTTCATCACAACAATTCTTAACATAAAGCCTCCCGCTATACTTCAATACCAAACACCACTATTCGTATGGGCGGTCCTGATCACAGCCGTACTTCTTCTCCTGTCTCTCCCAGTTCTTGCAGCAGGAATTACAATACTTCTGACAGATCGGAACCTAAACACCACATTCTTTGACCCCGCAGGAGGGGGCGACCCTATTTTATACCAGCACCTC----

>x63_G6YZTIE01BQE7H

--TCTATCCGGAAACCTTGCCCACGCAGGTGCATCCGTCGACCTAACCATCTTCTCCCTTCACTTAGCGGGGATTTCCTCTATTCTCGGGGCAATCAACTTCATTACTACAATTATTAATATGAAACCGCCTGCTATCTCCCAGTACCAAACACCGCTCTTCGTATGAGCCGTGTTAATTACTGCCGCCCTTCTACTCCTATCCCTCCCAGTTCTGGCCGCCGGGATTACTATACTTCTTACCGATCGAAACCTAAACACCACATTCTTTGATCCTGCAGGCGGTGGAGATCCAATTCTCTACCAACACCTG----

>x64_G6YZTIE01BZYRE

--TCTATCAGCAGGCATTGCTCACGCGGGAGCCTCAGTAGACCTGGGGATCTTCTCCCTACACCTAGCCGGTGCCTCTTCTATCCTAGGAGCAGTTAATTTTATAACAACCGTCATTAATATACGAGCCACAGGAATTACCATGGACCGAATACCCCTGTTTGTATGATCTGTATTTTTAACTGCCATTTTATTACTCCTTAGGCTCCCAGTCCTAGCGGGAGCTATTACAATACTTTTAACCGACCGGAACCTAAACACCGCGTTCTTCGATCCTGCTGGAGGGGGGGACCCCATCCTATATCAACACTTATTC-

>x65_G6YZTIE01A4GBJ

--CCTTGCGGGCAACTTGGCCCACGCCGGAGCATCCGTCGATCTAACAATTTTTTCTCTCCATCTCGCCGGTGTGTCCTCAATTCTAGGGGCCATCAACTTTATCACAACTATTATTAATATAAAACCTCCCGCCCTCTCACAATATCAAACGCCACTATTTGTATGGGCTGTATTAGTCACCGCTGTACTACTTCTTCTTTCCCTCCCCGTCCTTGCTGCAGGAATTACAATACTGTTAACCGATCGAAATCTTAACACAACCTTTTTTGATCCCACAGGAGGAGGAGACCCCATTCTCTACCAACACCTA----

>x66_G6YZTIE02DQ546

--ACTAGCGGGCAACTTAGCCCACGCAGGAGCATCCGTCGACCTTACCATTTTCTCCCTTCACCTAGCGGGTGTGTCATCAATCCTTGGCGCCATTAACTTCATCACCACAATTATTAACATAAAACCCCCAGCCATCTCTCAGTACCAAACACCCCTATTCGTATGAGCCGTTTTAATCACTGCCGTCCTCCTATTACTATCCCTTCCAGTACTCGCAGCAGGAATCACAATGCTTCTCACAGACCGAAACCTAAACACCACCTTCTTCGACCCTGCAGGCGGAGGGGACCCCATCCTCTACCAACACTTA----

>x67_G6YZTIE01BP4ZQ

--TCTATCCGCCGGAATTGCCCACGCAGGAGCATCTGTAGATCTCGGCATCTTCTCGCTTCATCTCGCAGGGGTATCGTCCATTTTAGGAGCAGTTAACTTCATAACAACAGTTATTAATATGCGATCTACAGGGATGACTATAGACCGGATACCACTATTTGTATGAGCTGTATTCTTAACAGCAATCCTACTACTTTTAAGACTCCCAGTGTTAGCCGGGGCCATTACTATGTTACTAACAGACCGAAACCTAGACACAGCATTCTTCGACCCAGCCGGCGGGGGAGACCCAATTCTCTATCAACATCTATTT-

>x68_G6YZTIE01CCUZG

--TCTCTCAGCAGGTATCGCCCATGCAGGATCCTCAGTGGACTTGGGCATCTTCTCTTTACATTTAGCAGGTGTATCTTCCATTTTAGGAGCAGTTAACATTATAACTACCGTTATAAACATACGAACAGTTGGAATTACAATAGATCAAATACCATTATTTGTATGAGCTATTTACCTCACAGCTATCTTACTTCTATTAAGACTCCCGGTCCTAGCAGGAGCTATCACTATATTACTAACTGATCGAAACTTTAACACAGCCTTCTTTGACCCAGCTGGAGGTGGAGATCCAATTTTATATCAACATCTATTC-

>x69_G6YZTIE02DKTEL

--ACTGTCAGGTAACTTAGCGCATGCAGGGGCATCTGTAGATTTAACCATCTTTTCACTACACTTAGCAGGAATTTCCTCAATCTTAGGAGCTATTAATTTTATTACAACAATTATTAATATGAAACCTCCAGCTATTTCTCAATATCAAACGCCTCTATTCGTATGAGCTGTTCTTATTACAGCTGTACTTCTACTCCTTTCACTTCCAGTTCTTGCAGCTGGAATTACAATACTGCTTACAGACCGAAACCTAAATACAACCTTCTTCGACCCTGCTGGGGGAGGGGACCCAATTCTTTACCAACACTTG----

>x70_G6YZTIE02DL5XG

--ACTAGCCGGAAATTTGGCCCATGCAGGTGCATCCGTAGATTTAACAATCTTCTCCCTTCACCTAGCCGGGATCTCATCAATTTTAGGGGCCATTAACTTCATCACCACTATTATTAACATGAAACCCCCAGCCATCACTCAATACCAA---CCCCTATTCGTCTGAGCAGTCCTCATTACAGCCGTTCTTCTACTACTTTCACTCCCCGTCCTCGCCGCGGGCATCACAATGCTTCTCACGGACCGAAACCTAAACACAACCTTCTTCGATCCGGCAGGAGGGGGAGACCCAATTCTCTACCAACACCTA----

>x71_G6YZTIE02ES8GC

--CTTATCAGGAAATCTAGCTCATGCTGGTGGCTCTGTAGATTTGGTTATTTTCTCTCTTCACTTGGCAGGTATTTCTTCTATTTTGGGTGCAATTAACTTTATCACTACAATCATTAATATGCGGTGGCAAGGAATGCAGTTTGAGCGGTTGCCATTGTTCGTGTGGTCAGTAAAAATTACTGCTATTTTATTGTTGCTCTCTTTACCTGTGTTGGCAGGAGCAATTACTATGCTTCTAACGGATCGAAATTTTAATACATCTTTCTTTGATCCCACAGGAGGTGGTGATCCGGTATTATTCCAACACTTATTT-

>x72_G6YZTIE01BDWJ3

--CCTTGCAAGAGGTATTGCTCACGCAGGAGCCTCAGTTGACATAGGCATTTTTTCCCTACATTTAGCCGGTGTATCCTCTATCCTAGGAGCAGTTAATTTTATAACCACAGTAATTAACATACGAACTGCAGGCATAACTATAGACCAAATGCCGCTATTTGTATGGGCAGTATTCTTAACTGCAATTTTATTATTACTCTCTCTACCCGTACTAGCCGGAGCTATTACAATATTATTAACTGATCGAAACTTAAATACATCATTTTTCGATCCTGCAGGAGGGGGGGACCCCGTTCTTTATCAACATTTATTC-

>x73_G6YZTIE01B1PSA

--TCTTGCTGGGGCTATTGCTCATGGGGGCGCTTCAGTTGATATGGGGATTTTTTCTCTTCATTTAGCTGGTGTATCCTCTATTTTGGGGGCTGTAAATTTTATGACCACTGTATTAAATATGCGGCCTAATGGTATAACGACAGATCGAATACCTTTATTTGTGTGATCAGTTTTTATTACAGCTGTGTTATTATTATTGTCATTACCTGTTCTAGCAGGAGCAATTACGATACTTTTAACTGATCGTAATTTAAATACA---TTTTTTGATCCTGCTGGAGGGGGTGATCCTGTACTTTATCAGCAC-------

>x74_G6YZTIE02C3RAT

--ATTATCAGGAAACTTAGCACATGCAGGGGCATCAGTTGACCTCACTATTTTTTCCCTCCATTTAGCAGGTGTCTCTTCAATTCTAGGGGCCATTAATTTTATCACAACTATTATTAATATGAAACCCCCAGCTATTTCACAGTACCAAACACCCCTGTTTGTTTGAGCTGTCCTAATTACAGCTGTACTTCTTCTTCTTTCCCTTCCAGTTCTTGCAGCAGGAATTACAATACTGCTAACGGACCGAAATCTAAATACTACTTTCTTCGACCCTGCAGGGGGAGGAGACCCGATCCTATACCAACACCTC----

>x75_G6YZTIE02EFBSN

--TTTATCAGGACCACAAACCCATTCAGGAGGATCTATAGATATGGCAATATTTAGCCTTCACTGTGCAGGTGCTTCATCTATTATGGGTGCAATAAATTTCATAACAACTATTTTCAATATGAGAGCCCCAGGGTTAACTTTAGACAAATTACCTTTGTTTGTTTGATCTGTATTAATTACTGCATTTTTATTATTACTATCTCTCCCAGTGTTAGCTGGTGCTATAACAATGCTATTAACAGATAGAAATTTTAATACAACATTTTTTGATCCTGCCGGAGGAGGAGATCCAGTATTATATCAACATTTATTT-

>x76_G6YZTIE02EC1KN

--TCTTGCAGGAGCTATTGCACATGCAGGTGCCTCAGTGGACATGGGTATTTTCTCTTTACATTTAGCAGGTGTATCTTCTATTTTAGGTTCTGTAAATTTTATAACAACAGTTATTAATATACGAGCAAAAGGTATAACAATAGACCGAATACCTCTATTCGTATGAGCTGTTTTTATTACAACTATTTTACTTTTACTTTCTTTACCTGTCTTGGCAGGAGCAATTACTATGCTTTTAACAGACCGTAATCTAAATACCTCATTCTTCGATCCTGCAGGAGGGGGTGATCCTGTTTTATATCAACATTTATTT-

>x77_G6YZTIE01BHU60

ACT---TCAGCAGGAATTGCCCACGCAGGAGCATCAGTAGACCTAGGAATTTTCAGTCTACACTTAGCCGGAGTATCATCCATTCTTGGTGCGGTTAACTTCATAACTACTGTTATTAACATACGAACGACAGGAATAACTATAGACCGTATACCTTTATTTGTCTGAGCTGTATTCTTAACAGCTATCCTTTTACTACTAAGACTCCCAGTTTTAGCTGGAGCAATCACAATACTTCTAACAGACCGAAATCTAAATACAGCATTCTTTGACCCGGCAGGAGGGGGAGATCCAATTTTATACCAACATTTATTT-

>x78_G6YZTIE02D2ECV

--TCTCTCAGCTGGAATTGCCCACGCCGGAGCATCAGTAGACCTGGGAATTTTTTCACTTCATCTAGCTGGAGTCTCCTCAATTCTAGGAGCCGTTAACTTCATAACAACGGTAATTAATATACGAACCACTGGAATGACTATAGACCGTATACCCCTGTTCGTCTGAGCTGTATTCTTAACAGCTATTTTACTATTACTCAGGCTGCCAGTTCTAGCAGGAGCTATCACTATGCTACTTACAGATCGAAACCTAAATACAGCCTTCTTCGACCCAGCAGGAGGCGGTGACCCAATTCTATACCAGCACTTATTT-

>x79_G6YZTIE02EHUFF

---TTAGCAGCAGCTATCGTCCACGCCGGAGCTTCAGTTGACTTACGAATTTTCTCTCTCCATCTTGCAGGT---TCATCTATTCTTACAGCCATAACTTTTATGACCACTGTTATTAATATATGGTCTTTTGCTATAATATTGGACAAAATACCCCTTTTCGTCTGATCTGTTTTTATTACAGCCATTCTTCTTCTCCTTTCTCTCCCAGTCCTTGCAGGAGCTATTACTATATTATTGACCGACCGAAATCTTAACACTTCCTTTTTTGACCCAGCTGGGGGTGGAGACCCTGTCTTGTACCAACATTTATTC-

>x80_G6YZTIE02ENPU7

--TCTAGCCGCAGGCATCGCTCATGCAGGAGCTTCTGTTGATATGGGAATTTTCTCTCTTCATTTAGCCGGAGTTTCTTCTATT---GGCGCAGTTAATTTTATAACAACTGTTATTAATATACGACCATCAGGAATAACTATAGATCGTATACCTTTATTTGTATGATCCGTATTCATTACTGCTATTTTACTTCTTCTTTCTCTACCTGTTCTAGCAGGAGCAATTACCATGCTTTTAACTGACCGTAATTTAAACACTTCTTTTTTCGATCCAGCGGGAGGTGGAG---------------------------

>x81_G6YZTIE01B0HR1

--CCTAGCCGCCGCTATTGCCCATGCAGGTGCCTCAGTAGACTTAGGGATTTTCTCTCTTCACTTAGCAGGTGTATCATCAATTCTAGGAGCAGTAAACTTTATAACAACAGTAATTAATATACGATCCTACGGCATAACTATGGACCAAATACCTCTATTTGTATGGGCCGTATTCATTACCGCCATCTTATTACTTCTGTCCTTACCAGTTCTAGCCGGAGCTATTACAATACTTCTAACAGACCGAAACTTAAATACATCCTTCTTTGATCCTGCAGGCGGTGGAGACCCCGTTTTATATCAACACTTATTT-

>x82_G6YZTIE02EBK4E

--ACTAGCTGCTCCTATCGCTCACGCAGGAGCTTCAGTTGATATAGGTATTTTCTCCCTACACTTAGCAGGTGTTTCCTCAATCTTAGGGGCCGTAAATTTTATAACTACAGTTATCAATATACGATCTTTTGGTATATCTATAGATCAAATACCTTTATTTGTATGAGCTGTATTTATTACAGCCATCTTATTGTTGTTATCTTTACCAGTTTTAGCAGGAGCTATCACAATACTTCTTACAGATCGAAATTTAAATACCTCTTTCTTTGACCCAGCAGGAGGAGGAGATCCTATTCTTTATCAACACTTATTC-

>x83_G6YZTIE02ECE6M

--TCTTGCTAGGGGAATTGCTCATGCAGGAGCCTCAGTTGATATAGGA---TTTTCTTTACACTTAGCAGGAGTCTCATCGATCTTAGGAGCAGTTAACTTTATAACTACTGTAATTAATATACGAGCCGCAGGAATAACAATAGACCAAATACCGCTATTCGTATGAGCAGTATTCCTTACTGCAATTTTACTTCTTCTCTCCCTCCCAGTTCTAGCTGGAGCCATTACTATACTTTTAACTGACCGAAATCTTAATACATCCTTCTTTGACCCGGCAGGGGGAGGGGACCCAGTTCTATACCAACACTTATTT-

>x84_G6YZTIE02C6OX1

--TCTAGCAGCCGCTATTGCACACGCAGGTGCCTCAGTTGATCTGGGTATTTTCTCTCTCCACCTAGCCGGTGTGTCCTCTATTCTTGGAGCTGTTAATTTCATGACCACTGTCATTAATATGCGATCATTCGGTATAAAAATAGACCAAATGCCACTTTTCGTATGATCAGTTTTTATTACAGCAATTCTACTTCTACTATCTCTACCTGTTTTGGCAGGAGCTATTACGATGCTATTAACTGACCGTAATCTAAATACTTCTTTCTTCGACCCTGCCGGAGGAGGAGATCCTGTTCTCTATCAACATTTATTC-

>x85_G6YZTIE02C2IMS

--ACTATCAGCAGCTATTGCTCATGCAGGAGCTTCAGTAGATTTAGGAATTTTTTCTTTACATTTAGCAGGTGTTTCTTCTATTTTAGGAGCAGTAAATTTTATAACCACAGTAATTAATATACGGCCATTAGGTATAACTATAGATCGAATACCTTTATTTGTATGAGCTGTATTTATTACGGCTATCTTACTTCTTCTTTCTCTTCCGGTATTAGCAGGAGCTATTACTATATTATTAACAGATCGTAATTTAAACACTTCCTTTTTTGATCCTGTAGGAGGGGGTGATCCTGTACTTTA--------------

>x86_G6YZTIE02DI53Q

--TCTTGCTGCTGCTATT---CATGCCGGAGCTTCGGTAGATATAGGAATTTTCCCTTTACATTTAGCAGGTGTATCATCTATTCTAGGAGCAGTTAATTTTATAACTACTGTAATTAATATGCGATCCTTTGGTATAACTATAGACCAAATACCTCTTTTTGTTTGAGCAGTGTTTATTACCGCCATCCTTCTTCTTCTCTCCCTTCCTGTCTTGGCAGGTGCCATTACAATGCTTCTTACAGACCGAAATTTAAATACATCCTTTTTTGACCCTGCTGGAGGAGG-----------------------------

>x87_G6YZTIE02EGNA3

--ACTAGCGGGCAATCTAGCACATGCCGGAGCATCCGTTGACCTTACTATCTTCTCCCTCCACCTCGCAGGTATCTCCTCTATCCTGGGAGCTATTAATTTTATTACCACAATTATTAATATGAAGCCCCCTGCAATTTCACAGTACCAAACACCCCTGTTCGTATGGGCTGTTCTAATTACAGCCGTCCTACTTCTTCTGTCGCTCCCCGTGCTTGCCGCTGGCATTACGATGCTGCTGACAGACCGAAACCTAAATACAACCTTCTTCGACCCGGCGGGAGGAGGAGACCCAATCCGTTTATCAGCACCT----

>x88_G6YZTIE01BR7YV

--TCTTGCTGCTGCTATTGCCCACGCTGGAGCTTCAGTTGACATAGGGATTTTCTCTCTTCATCTGGCAGGTGTCTCTTCCATTCTAGGGGCAGTTAATTTTATAACTACCGTTATCAATATACGCTCATTTGGGATAACTATAGACCAAATACCCCTCTTCGTATGAGCAGTGTTTATTACTGCCATTCTTCTTTTATTATCTTTGCCAGTCTTAGCTGGAGCTATTACCATACTTCTCACTGATCGAAATTTGAATACATCCTTTTTTGATCCAGCCGGGGGAGGAGACCCAGTACTCTATCAACATTTATTC-

>x89_G6YZTIE02DJIZM

--TCTCGCTGCAGGAATTGCCCATGCTGGTGCATCCGTCGATATAGGAATTTTCTCACTCCATCTAGCAGGAGTATCTTCAATTTTAGGAGCTATTAATTTTATAACAACAGTTATTAATATACGACCTAAAGGT---ACAATAGACCGTATACCTTTATTTGTTTGATCTGTTTTTATTACCGCAATTTTACTACTCCTATCACTACCTGTACTAGCAGGGGCCATTACTATACTACTAACTGACCGAAATCTTAGTACTTCCTTCTTTGATCCAGCCGGAGGGGGAGATCCAATTCTATATCAACATTTATTT-

>x90_G6YZTIE02DC6SD

---CTTGCTGCCGCTATCGCTCATGCTGGTGCATCAGTTGATATAGGAATTTTTTCGCTTCATTTGGCAGGGGTATCCTCTATTTTAGGTGCCGTTAATTTTATAACAACTGTTATTAATATACGATCCTTCGGTATAACCATAGACCAAATGCCTTTATTTGTGTGAGCTGTTTTTATTACAGCCATCTTATTGTTATTATCCTTACCAGTATTAGCAGGTGCTATTACCATGCTCCTTACTGATCGAAATCTTAATACTTCCTTTTTTGACCCAGCTGGAGGAGGGGATCCAGTTCTCTACCAACATTTATTT-

>x91_G6YZTIE02DV80L

--TTTAGCAGGGCCACAAACACACTCTGGAGGTTCAGTAGATATGGCT---TTTAGTTTACACTGTGCAGGTGCATCATCCATTATGGGTGCCATAAATTTTATAACAACCATATTTAATATGAGAGCTCCAGGTTTAACAATGGATAAATTACCTTTGTTTGTTTGATCAGTTCTAATTACCGCATTTTTGTTATTACTATCTTTACCCGTATTAGCAGGGGCAATAACAATGTTATTAACAGATAGAAATTTTAATACAACCTTTTTTGACCCAGCAGGGGGAGGTGATCCAGTTTTATATCAACATTTATTT-

>x92_G6YZTIE01CF0JN

--TCTGGCTGCCGCAATCGCTCATGCAGGAGCATCTGTAGACATAGGGATTTTCTCCCTACACTTAGCGGGGGTCTCCTCTATCTTAGGAGCCGTAAACTTTATAACTACCGTTATAAACATACGATCATTTGGAATAACTATAGACCAAATACCTCTATTTGTCTGAGCAGTATTTATTACTGCTATTTTACTTCTTTTATCCCTTCCAGTTCTTGCAGGAGCTATTACAATACTCCTTACTGACCGAAACTTAAATACATCTTTCTTCGACCCTGCCGGAGGAGGGGACCCCGTTTTATACCAACACTTATTC-

>x93_G6YZTIE01B79TD

--TCTAGCTGCTGCTATTGCCCACGCAGGTGCTTCAGTTGATATAGGTATTTTCTCACTTCATTTAGCAGGAATTTCTTCCATCTTAGGAGCAATCAACTTTATAACCACAGTCATTAATATACGATCTTTCGGTTTAACTTTAGATCAAATACCACTTTTTGTATGAGCAGTTTTTATTACTGCTATCCTTTTACTACTTTCTCTTCCCGTTCTAGCTGGAGCTATTACTATACTCTTAACCGATCGAAATTTAAACACCTCATTTTTTGACCCAGCTGGAGGTGGAGATCCTATTCTCTATCAACACTTATTT-

>x94_G6YZTIE02D0WAE

---CTAGCAGCCGCCATCGCTCATGCTGGAGCTTCAGTAGACTTAGGTATCTTCTCACTCCATCTAGCAGGTGTATCCTCCATTCTTGGGGCTGTAAACTTTATAACCACTGTTATTAACATACGGTCGTTTGCTATAACTATAGACCAAATACCTCTCTTTGTTTGGTCTGTATTTATTACCGCTATCCTCCTTCTCCTTTCCCTACCAGTTCTCGCCGGAGCTATTACTATGTTGCTAACAGACCGTAACCTTAACACTTCATTTTTCGACCCAGCTGGTGGGGGAGATCCTGTCTTATACCAACACTTATTC-

>x95_G6YZTIE02EM5GE

--TCTATCAGCAGGAGTTGCCCACGCTGGGGCCTCAGTAGACCTGGGGATTTTCTCCCTCCACCTAGCTGGAGTCTCATCCATTCTAGGAGCAGTTAATTTTATAACTACTGTCATTAATATACGAACCACAGGGATAACTATAGACCGGATGCCCCTATTTGTTTGAGCTGTATTCCTAACAGCTATTCTCCTACTATTAAGACTACCCGTTCTAGCTGGAGCTATTACCATACTACTCACAGATCGAAACCTCAACACAGCCTTCTTCGATCCCGCCGGAGGTGGGGACCCAATCCTTTACCAACACCTATTC-

>x96_G6YZTIE01BFRIT

--CACACACTCTGGAATTGCCCACGCAGGGGCATCAGTAGACTTAGGGATTTTCTCCCTCCACCTAGCAGGTGTTTCTTCTATCCTAGGAGCAGTTAATTTTATAACTACCGTTATTAATATACGAGCAACAGGAATAACTATAGACCGAATACCCCTATTCGTCTGAGCCGTATTCTTAACAGCTATCTTATTACTCTTAAGACTACCAGTTCTAGCCGGAGCAATCACCATACTGCTAACAGACCGAAACCTTAATACAGCTTTCTTCGACCCAGCCGGAGGTGGCGACCCTATTCTATATCAACACCTA----

>x97_G6YZTIE01CFD6F

--TCTTAGAAGAAACATTGCTCACTACGGACCCTCGGTGGACTGCGCTATTTTTTCGCTCCACTTAGCGGGAATTTCTTCAATTTTGGGTGCCATAAATTTTATTACAACAATTTTGAATATACGAGTATTAGGGATAATACTAGATCAAATGCCTTTGTTTGTATGATCTGTTCTTATTACTACCGTGCTACTTTTACTTGCTTTACCAGTATTAGCCGGAGCAATTACTATACTATTAACCGACCGGAATCTTAATACCTCTTTTTATGACCCAGCAGGGGGGGGAGACCCGATTCTTTACCAACACTTGTTT-

>x98_G6YZTIE01B79I0

--TCTTGCAGCTGCTATTGCTCATGCAGGGGCTTCTGTAGATATAGGAATTTTTTCTCTTCATCTTGCAGGGGTCTCCTCTATTTTAGGAGCGATTAATTTTATAACAACGGTTATTAATATACGCCCTCGTGGAATAAGGATAGGTCGAATACCTTTATTCGTATGGTCTGTATTTATTACTGCTATTTTATTATTATTATCTCTTCCTGTGCTTGCAGGTGCAATTACTATACTTTTAACAGACCGTAATATTAATACTTCTTTCTTTGATCCTGCTGGTGGAGGTGATCCTGTCTTATATCAACA--------

>x99_G6YZTIE01B7CHA

--CTTATCAGGACCCCAAACCCACTCAGGAGGATCAGTAGATATGGCTATATTCAGTTTACATTGTGCAGGTGCTTCATCAATAATGGGTGCTATAAATTTTATAACAACAATATTTAACATGAGAGCACCAGGTTTAACCATGGATAAATTACCTTTATTCGTTTGATCTGTTCTGATTACAGCATTTTTATTATTATTATCATTACCAGTACTAGCAGGTGCTATCACCATGTTATTAACCGATAGAAATTTCAACACAACATTCTTCGATCCCGCAGGAGGAGGAGATCCAGTTTTATATCAACATTTGTTT-

>x100_G6YZTIE02D9KZD

--TTTAGCTGCTGCTATTGCCCACGCCGGAGCCTCTGTGGATATAGGCATTTTCTCCTTGCATTTAGCAGGTGTTTCATCAATTCTAGGAGCCGTTAATTTTATAACTACCGTTATTAATATACGCTCATTTGGTATAACTATAGATCAAATACCTCTTTTTGTCTGAGCAGTATTTATTACTGCTATTCTTCTTCTACTTTCCCTGCCCGTTTTAGCAGGTGCAATTACCATACTCCTTACCGATCGTAATTTGAATACTTCCTTTTTTGACCCTGCCGGAGGGGGAGACCCAGTTCTATGTCAACACTTATTT-

>x101_G6YZTIE02EAGJW

--GCTAGCGGGTAATTTAGCCCATGCCGGAGCATCCGTAGACTTAACTATCTTCTCCCTCCACCTCGCAGGGATTTCTTCAATTCTTGGAGCCATCAATTTCATTACAACCATCATTAACATGAAACCCCCTGCTATTTCACAGTACCAGACTCCCCTATTTGTATGGGCAGTCCTGATTACTGCTGTCTTGCTCCTTCTCTCTCTTCCGGTTCTTGCTGCCGGAATTACAATGCTCCTTACAGACCGAAACTTAAACACCACCTTCTTCGACCCTGCAGGAGGAGGAGACCCAATTCTCTACCAACATCTA----

>x102_G6YZTIE01BELHY

--CCTGTCGGGAAACCTGGCTCACGCAGGGGCCTCTGTAGACCTAACTATTTTCTCCCTCCACCTGGCAGGTATTTCCTCAATCCTGGGAGCAATTAATTTTATTACTACCATCATTAACATGAAACCCCCTGCTATTTCTCAATATCAAACCCCACTCTTTGTATGAGCCGTTCTCATCACTGCTGTCCTTCTACTACTCTCTCTCCCAGTCTTAGCTGCTGGCATCACCATGCTCCTAACTGATCGAAACCTAAATACCACCTTCTTCGACCCAGCGGGAGGAGGGGACCCAATTCTGTACCAACACCTA----

>x103_G6YZTIE01CCQLZ

--TCTTGCAAGAGGAATTGCCCACGCTGGTGCTTCAGTAGATATAGGAATCTTCTCTCTCCATTTAGCGGGGGTATCATCGATTCTAGGAGCAGTAAATTTTATAACCACAGTTATTAACATACGAGCAGCTGGAATAACCATGGATCAGATGCCACTATTTGTCTGAGCAGTATCCCTAACTGCAATCTTACTACTTCTATCCCTCCCAGTGCTAGCAGGGGCCATCACCATATTACTAACGGACCGAAACCTAAACACCTCATTTTTTGACCCAGCAGGGGGTGGTGATCCAGTTCTGTATCAACACCTATTC-

>x104_G6YZTIE01B2GVM

---CTTGCCAGAGGAATCGCCCATGCAGGAGCATCCGTTGATATAGGA---TTTTCTCTCCACTTAGCAGGAGTATCTTCAATTTTAGGGGCCGTTAACTTTATTACAACAGTAATTAACATACGAGCAACAGGTATAACTATAGACCGCATGCCTTTATTTGTGTGGGCCGTTTTTTTAACAGCCATTCTTTTATTACTATCTCTGCCCGTCCTTGCAGGAGCGATTACAATATTATTAACAGACCGAAATTTAAATACCTCTTTCTTTGACCCCGCAGGAGGAGGAGATCCAATTTTATATCAACATTTATTT-

>x105_G6YZTIE02C6AMO

--GTTGGCCGGAAGAATCGCACACGCAGGGGCTTCAGTAGATATAGGAATCTTCTCTCTCCACCTTGCCGGAGTTTCATCAATCTTAGGAGCCGTAAACTTCATCACAACTGTGATTAACATACGCTCATCAGGAATAACCATAGACCGTCTCCCACTATTTGTCTGAGCTGTCTTTCTCACAGCAATTCTACTTCTCCTCTCACTACCAGTTTTAGCAGGAGCAATTACAATACTACTCACAGACCGAAACCTAAACACCTCATTCTTCGACCCGGCCGGAGGAGGAGACCCCATCCTATACCAACATTTA----

>x106_G6YZTIE02C8ZBQ

---TTATCAGCTGGTATCGCTCATTCGGGGCCTTCAGTTGATTTTTTAATTTCCTCTCTTCACTTACTAGGTGTAGGTTCTATTCTTGGATCAATCAATTATATAACAACCATTATTAACATACGGGCCACAGGTATATCTTTTGATCGAATACCTCTTTTTGTCTGGGCAATCCTGATTACATCTATTCTTCTCATACTCTCTCTCCCAGTTTTAGCCGGGGCTATCACAATACTTTTAACGGACCGAAACTTTAATACATCTTTTTTTGATCCAGCAGGAGGAGGGGACCCAGTTCTATTTCAACACCTTTTC-

>x107_G6YZTIE01B8PI0

--CTTAAGGGCAAATTTGGCGCACGCGGGGGCATCAGTTGATTTTGCTATTTTTTCTTTACATTTAGCTGGAATTTCTTCGTTGCTGGGGGCGGTCAATTTTATTAGAACATTAGGAAATTTACGTTCCCTTGGGATAAGGCTAGATGTTGTGCCTTTGTTTGCTTGAGCGGTTTTTATTACGGCTGTTTTATTGCTCCTATCCTTACCTGTCTTAGCTGGGGCTATTACTATGCTTCTGACTGATCGTAATTTAAATACAGCGTTTTATGATGTCGCAGGAGGTGGAGACCCGGTACTTTACCAGCATTTATTT-

>x108_G6YZTIE01A3JJQ

--TTTAAGGAGAAATATTGCACATTCAGGAGCATCGGTCGATTTTGCTATTTTTTCATTACATTTAGCCGGAATTTCTTCCTTACTAGGGGCAGTAAATTTTATTAGGACACTAGGGAATTTACGGGTGTTAGGTTTAGTTTGAGACACAGTGCCTTTATTTGGGTGAGCTGTTTTTATTACAGCCATTCTCCTTTTACTATCCCTACCAGTGTTAGCAGGAGCTATCACTATATTACTTACAGATCGAAATTTAAATACAACTTTTTACGATGCAGCGGGCGGAGGAGATCCTGTTTTATACCAGCATTTATTT-

>x109_G6YZTIE01CIS83

--ATTAGCTGGTAACCTGGCCCACGCCGGAGCATCCGTAGATTTAACTATCTTCTCCCTCCATCTCGCAGGAATCTCGTCAATTCTAGGGGCAATTAACTTCATTACCACTATTGTTAACATGAAACCTCCTGCAATCTCGCAGTACCAGACACCCTTGTTCGTCTGAGCTGTTCTTATTACGGCCGTCCTCCTTCTCCTATCCCTCCCTGTTCTTGCTGCCGGGATTACTATGTTGCTCACCGACCGTAACTTAAACACTACTTTCTTTGACCCAGCAGGGGGTGGAGACCCCATCCTATACCAACACCTA----

>x110_G6YZTIE01ARLS8

---TTAGCTGGTAACCTGGCCCACGCCGGCGCATCTGTTGACCTCACAATTTTCTCCCTTCACTTGGCAGGAATCTCCTCAATTCTCGGTGCCATTAACTTTATCACAACTATTATTAACATAAAACCCCCAGCTATTTCTCAATACCAGACTCCTTTATTTGTTTGAGCCGTTCTAATTACAGCCGTTCTTCTTCTTCTCTCCCTCCCCGTCCTTGCTGCCGGCATTACAATGCTCCTTACAGACCGAAATTTAAATACAACATTCTTTGACCCAGCCGGGGGAGGAGACCCAATCCTGTACCAACACCTA----

>x111_G6YZTIE01BQLL4

--TTTAGCATCTACTGCTGCTCACAGTGGTCCCTCAGTAGATTTGGCTATTTTCTCTCTTCATTTGGCTGGTGTCTCTTCAATCCTTGGAGCAGTAAACTTCATGTCTACTGTAATCAACATACGAATCACAAAAATGTTCTATCACATAATATCTTTATTCACATGATCAATTTACATTACTACTATCCTCCTACTCCTTTCTCTACCAGTCTTAGCCGGAGCTATTACTATACTATTAACAGATCGTAAC----------------------------------------------------------------

>x112_G6YZTIE02EAS6Z

--TCTTGCCGCTGCTATTGCCCACGCTGGAGCATCAGTTGATATGGGAATTTTTTCGCTTCATTTGGCCGGTGTCTCATCTATTCTAGGGGCTGTAAATTTTATAACCACAGTTATTAATATACGCTCATTTGGTATAACTATAGACCAAATGCCTTTATTTGTCTGCGCTGTATTTATTACTGCTATTTTACTTTTATTATCATTGCCTGTTTTAGCCGGAGCTATTACGATACTTCTTACTGATCGTAATTTAAATACATCATTTTTTGATCCAGCCGGAGGTGGAGATCCCGTCCTTTATCAACACTTATTT-

>x113_G6YZTIE02EQCDJ

--CCTAGCAGGAAACCTAGCCCACGCAGGAGCTTCCGTTGATCTAACCATCTTTTCGCTTCACCTGGCAGGTATTTCCTCAATCCTAGGGGCCATCAACTTCATCACAACAATTATCAACATGAAACCTCCAGCCATCTCTCAATACCAGACACCTCTGTTTGTTTGAGCAGTCCTAATTACGGCTGTCCTTCTTCTCCTATCCCTCCCTGTCCTTGCTGCTGGCATCACTATGCTTCTAACGGACCGAAACCTAAACACTACCTTCTTCGACCCAGCTGGAGGAGGAGACCCAATCCTTTATCAACACCT-----

>x114_G6YZTIE02D0O6I

--ACTATCAGCAGGAGTTGCCCACGCAGGAGCCTCAGTCGACCTAGGAATTTTCTCCCTCCACCTAGCAGGAGTCTCATCCATTTTAGGAGCAGTTAATTTCATAACTACTGTTATCAACATACGAACAACAGGAATAACTATAGATCGAATACCCCTATTTGTTTGAGCCGTATTCCTAACAGCTATTCTCCTACTCCTTAGACTACCCATTCAAGCTGGAGCTATTACTATATTACTTACAGATCGAAACATCAATACAGCCTTCTTTGATCCCGCCGGAGGGGGTGACCCAATCTTATATCAACACCTA----

>x115_G6YZTIE01ATHCT

--CCTTAGAGATACCATTGCCCATGCTGGCTCATCAGTGGATTTTGCTATTTTCTCACTTCATTTAGCCGGGGTTTCTTCATTACTTGGCGCGGTAAATTTTATCACTACAATTTGTAATCTACGAATTTTAGGAATAAGGTTCGACCGAATACCTATATTCCCCTGAGCTGTGTTAATTACGGCAATCCTCCTACTACTATCTTTACCGGTTCTAGCGGGGGCAATTACTATATTATTAACTGATCGAAATTTAAACACCTCTTTTTACGATGTAAGAGGAGGAGGAGATCCGGTTCTCTATCAGCATTTATTC-

>x116_G6YZTIE01AYGKA

--CTTATCTGCGGCAATTGCCCATGCTGGGGCATCAGTAGATCTAGGTATTTTTTCTCTTCATTTAGCGGGTGTATCGTCTATTTTGGGGGCTATTAATTTTATAACAACTGTAATTAATATGCGTCCTCAGGGAATGAGAATAGACCGAATGCCATTATTTGTGTGATCAGTCTTTATTACCGCTATTCTATTATTATTATCCTTACCGGTGTTAGCTGGTGCTATTACTATACTACTAACAGATCGAAATTTAAACACTTCTTTCTTTGATCCGGCAGGCGGAGGAGATCCAGTACTCTATCAGCACCTATTT-

>x117_G6YZTIE01A7LDQ

--TTTAGCTGCGGGAATTGCTCATGCAGGAGCATCTGTAGATTTAGGAATTTTTTCACTTCATCTTGCAGGTGTTTCTTCAATTTTAGGAGCTATTAACTTTATAACTACTGTTATCAATATACGACCTAAAGGAATAACTATAGACCGTATACCTTTATTTGTATGATCTGTTTTTATTACAGCTATTTTATTATTATTATCATTACCTGTTTTAGCAGGGGCTATTACAATATTATTGACTGATCGAAATTTAAATACTTCATTTTTTGATCCCGCAGGTGGAGGAGATCCTATTTTATATCAACATTTATTT-

>x118_G6YZTIE01BGIXT

--ACTCTCAGCAGGTATCGCCCACGCAGGAGCTTCTGTAGACTTAGGAATTTTTTCTCTACACCTAGCCGGTGTATCCTCGATCCTAGGAGCAGTCAACTTTATAACTACTGTTATTAACATACGAACAACGGGAATAACTATAGACCGAATACCTCTATTCGTCTGAGCCGTATTCTTAACAGCTATCCTTCTTCTTTTAAGACTCCCAGTCCTAGCCGGAGCTATCACTATACTATTAACCGACCGAAATCTTAATACAGCCTTCTTTGACCCCGCCGGAGGAGGTAACCCTATTTTATATCAACACCTATTC-

>x119_G6YZTIE02DOUTQ

--CCTTGCAAGAGGAATTGCCCACGCAGGAGCCTCTGTAGATATAGGGATCTTTTCCCTACACCTAGCAGGTGTATCATCAATTCTAGGAGCAGTAAACTTTATAACCACAGTTATTAATGTACGAGCAGCTGGAATAACTATAGACCAAATACCACTATTCGTTTGAGCAGTATTTCTGACCGCAATTTTACTGCTCCTATCTCTACCAGTGTTAGCCGGGGCTATTACAATACTACTTACCGACCGAAACCTAAACACTTCGTTCTTCGACCCAGCAGGCGGTGGGGACCCAGTTCTATATCAACACCTATTC-

>x120_G6YZTIE01B5DBG

--TCTTGCGGCTGCTATTGCTCATGCAGGAGCCTCAGTAGATATAGGAATTTTCTCACTTCATTTGGCTGGTGTATCATCTATTCTTGGAGCTGTAAATTTTATAACTACAGTTATTAATATACGATCCTTCGGTATAACTATAGATCAAATACCTTTATTTGTGTGAGCCGTATTTATCACTGCCATTTTACTTTTATTATCACTACCTGTCCTAGCTGGGGCTATTACCATACTCCTTACTGATCGTAATCTTAACACCTCTTTCTTTGATCCTGCTGGAGGAGGAGATCCTGTTTTATACCAACATTTATTT-

>x121_G6YZTIE01A8ZNY

--TCTAGCTGCTGCTATTGCTCATGCTGGAGCTTCCGTTGATATAGGAATTTTTTCTCTCCATTTAGCAGGAGTTTCTTCCATTTTAGGTGCTGTAAACTTTATAACTACCGTCATTAATATACGATCCTTCGGTATAACTATAGACCAAATGCCTTTATTTGTATGAGCTGTATTTATTACTGCTATTCTACTCCTGCTTTCTTTACCTGTTCTAGCCGGTGCTATTACTATACTTCTCACTGATCGAAATTTAAATACATCATTTTTTGATCCTGCAGGAGGGGGAGACCCAGTTCTATATCAACATTTATTT-

>x122_G6YZTIE02C5QWW

--CTTAGCAGCCGCTATTGCACATGCAGGGGCCTCAGTTGATTTAGGTATTTTCTCCTTACATTTAGCTGGTGTTTCTTCTATTTTAGGAGCAGTTAATTTTATAACGACTGTCATTAATATACGATCATTCGGTATAAAAATAGATCAAATACCTTTATTTGTATGGTCTGTTTTTATTACAGCAATTCTACTTTTATTATCTTTGCCTGTTCTTGCGGGTGCTATTACTATACTATTAACAGATCGAAATCTTAATACTTCATTCTTTGACCCTGCTGGAGGTGGGGACCCTGTCTTATATCAGCACTTATTC-

>x123_G6YZTIE01BF69B

---CTATCGGGCATACTAGTGCATTCGGGCGGGTCGGTGGATATGGCTATTTTTAGCTTGCACTTGGCGGGACTGTCGTCGATATTAGGGGCAATCAACTTTACAACGACGATACTAAACATGCGTGCGCCCGGGATGACGATGGACCGGCTGCCGTTGTTTGTTTGGTCCATACTTGTAACAGTGGTGCTGTTGCTGTTATCGCTGCCGGTGCTGGCTGGCGCAATAACAATGTTATTGACAGATCGGAACTTTAACACAACGTTCTTTGACCCGGAGGGGGGCGGCGACCCGATATTGTTTCAGCATTTGTTT-

>x124_G6YZTIE01BF1HZ

-CGTTATCAGGGGTTATTGCTCACGCCGGTCCGGCAGTGGACTTAGGTATCTTCTCCCTTCACCTTGCTGGGGTCTCATCTATTCTAGGGGCTGTAAATTTTATTACAACTGTAATAAATATACGCCCTAAAGGAATAACTATAGACCGAATGCCTCTCTTCGTTTGAGCTGTGTTTTTAACAGCCATCCTTTTATTATTATCTCTTCCTGTATTAGCAGGCGCAATTACCATGTTGCTAACAGATCGTAACTTAAATACCTCATTCTTCGATCCAGCGGGAGGTGGGGATCCTATTCTCTATCAACATCTATT--

>x125_G6YZTIE01CAHQC

---GTTTCAAGAAACATTGCACACGCAGGACCATCAGTAGACCTAGCAATCTTTTCTCTACACCTAGCAGGTGCCTCGTCCATCTTAGGCTCAATCAACTTTATCACCACCGTAGCCAACATGCGAAACGAAGGATTCCGCCTAGAACGAATCCCCCTCTTCGTCTGGGCAGTAGTAATTACAACTGTCCTTCTCTTACTATCCCTGCCCGTTCTAGCTGGAGCAATTACAATACTCCTTACCGATCGAAATATCAACACAACCTTCTTCGACCCCGCCGGAGGAGGAGACCCAGTCCTCTTCCAAC---------

>x126_G6YZTIE01BXUGB

---CTAGCAGGCAACTTAGCCCACGCAGGAGCCTCCGTGGACCTGACAATCTTCTCCCTACACTTAGCCGGAATCTCGTCTATTCTAGGAGCAATCAATTTCATTACAACAATCCTTAACATAAAACCCCCGGCCATCTCTCAATACCAGACACCACTGTTTGTGTGAGCTGTGCTAATTACTGCTGTTCTTCTTCTTCTCTCATTACCAGTCCTTGCTGCAGGTATTACAATACTCTTAACCGACCGAAACCTCAACACAACCTTCTTTGACCCGGCCGGAGGGGGGGATCCTATTCTGTACCAACACTTA----

>x127_G6YZTIE01AO8OS

--TTTAGCGGCAGCTATTGCTCATGCAGGGGCTTCTGTTGATATAGGAATTTTTTCTCTTCATTTAGCAGGGGTATCTTCTATTTTAGGGGCAATTAACTTTATAACTACGGTAATTAATATGCGGCCTCGAGGTATGAGAATGGATCGAATACCTCTATTTGTATGATCAGTGTTTATTACAGCTATTCTATTGTTATTATCCTTGCCTGTTCTAGCAGGGGCAATTACAATGCTTTTAACTGACCGAAATATTAACACTTCTTTTTTTGATCCTGCCGGTGGGGGAGATCCTGTTTTATATCAGCATTTGTTT-

>x128_G6YZTIE01BI86F

---CTCTCAGCTGGAATCGCCCACGCAGGAGCCTCCGTAGACTTGGGAATTTTCTCCCTACACCTGGCCGGTGTATCCTCAATCCTAGGAGCAGTCAACTTTATGACCACCGTTATTAACATACGAACAACGGGGATGTCTATAGACCGAATACCGCTATTCGTATGAGCTGTCTTTTTAACAGCTATTCTCCTCCTCTTAAGACTACCAGTTCTAGCCGGAGCCATTACCATGTTATTAACTGATCGTAACCTGAATACAGCTTTCTTCGACCCCGCAGGAGGAGGAGACCCAATCCTTTATCAACACTTATTT-

>x129_G6YZTIE01AKY17

---CTAGCCAGAAATATTGCCAATGCTGGTCCCTCCGTAGATCTTGCAATTTTCTCTCTTCATTTAGCCGGGGTATCCTCCATTTTAGGCGCCCTTAACTTTATTACTACTATAATAAATATACGATCCGCAGGCCTTCAACTAGAACGAGTTCCTTTACTAGTATGATCTCTAGGAATCACCGTCCTTCTTCTTCTCCTGGCCTTACCCGTTCTAGCAGGAGCCATCACTATACTCCTCACTGACCGAAATCTAAATACAGCATTCTTTGACCCTACGGGCGGAGGAGACCCTATCCTTTACCAGCACCTCTTC-

>x130_G6YZTIE02DE2U2

--ATTAGCAGGCAACCTAGCCCACGCAGGAGCTTCCGTTGACCTTATTATTTTCTCCCTTCACTTAGCAGGCATTTCTTCTATTTTAGGGGCTATTAATTTTATTACAACGATTCTAAACATAAAACCGCCTGCTATATCACAGTATCAGACCCCTCTTTTCGTCTGAGCCGTCCTCATCACTGCTGTTCTCCTTCTTCTTTCCCTGCCCGTCCTAGCTGCCGGTATTACTATGCTACTAACAGACCGGAACTTAAACACCGCATTCTTTGACCCTGCTGGAGGAGGAGACCCCATTCTATACCAACACCT-----

>x131_G6YZTIE02DI5KX

-CGCTATCAGGTGTAATCGCCCACGCTGGCCCCACAGTGGATTTAGGGATCTTCTCACTTCACTTAGCAGGAGTCTCTTCCATCCTAGGGGCAGTTAATTTCATCACCACAGTTATAAATATACGGCCTAAAGGGATAACTATAGACCGTATACCTTTGTTCGTTTGAGCAGTGTTTTTAACCGCAATTTTATTACTATTATCCCTGCCTGTTTTAGCTGGCGCTATTACCATACTACTAACAGACCGTAATCTAAACACCTCATTTTTCGACCCGGCCGGGGGCGGAGATCCCATCCTCTACCAACACTTATT--

>x132_G6YZTIE02D138B

--TCTAGCCGGGAGTATCGCCCACGCCGGAGCTTCAGTCGATATAGGAATTTTTTCTCTTCATCTAGCAGGAGTATCTTCTATTCTTGGTGCTGTAAATTTTATAACAACTGTAATTAACATACGAAGAGAAGGTATAACAATAGACCGAATACCTCTATTTGTTTGGTCAGTCTTTTTAACAGCTATTTTGCTTTTACTTTCTCTTCCTGTTTTAGCGGGAGCTATTACCATACTACTTACTGACCGAAATCTAAACACATCGTTCTTTGACCCTGCGGGAGGAGGGGATCCTATTTTATATCAACATTTATTC-

>x133_G6YZTIE01AKE6I

--CGTATCAGGCAATATTGCACATAGTGGACCATCAGTAGATATAGCCATCTTTTCACTCCACTTAGCAGGGGCAAGATCAATTCTAGGAGCAGCCAATTTCATCGCTACCATCTTTAATATACGCACCAAACCACAAAAGGCCGAAAAAACTCCTTTATTTCCATGGTCAGTTATCCTCACTGCAGTACTACTTCTCCTCTCTCTACCCGTACTCGCGGGAGCCATCACAATACTCCTAACTGACCGTAATTTAAATACTACATTCTTCGAAAGAAGAGGAGGTGGAGATCCCGTTCTATACCAACACTTATTC-

>x134_G6YZTIE01A97K6

--GCTTTCAGGAAATCTAGCTCATGCCGGGCCCTCAGTAGACTTAGCAATCTTCTCTCTTCATCTGGCAGGGATCTCATCAATCTTAGGTGCCCTTAATTTTATCACGACTGTAATCAATATACGATGAAAAGGACTCCGATTAGAACGAATCCCGCTATTTGTATGAGCAGTGGTAATCACGGCAGTACTTTTA---TTATCTCTCCCCGTATTAGCAGGGGCAATTACAATGCTTCTCACCGACCGTAATCTTAATACTGCGTTCTTTGACCCGGCAGGTGGGGGAGACCCTATTCTATACCAACATTTATTC-

>x135_G6YZTIE02EQKDC

--ACTCTCAAGAAATTTAGCTCATATAGGACCATCAGTTGATCTAGCTATTTTTTCCCTTCACCTAGCAGGAATTTCATCTATTCTTGGAGCCATTAATTTCATTACTACTATTATTAATATACGATGAGAAGGTATACTAATAGAACGACTCCCTTTATTCGTTTGATCTGTATTTATTACTGCAATTTTATTACTTTTATCACTTCCAGTGTTAGCCGGAGCAATTACTATACTATTAACAGACCGAAACTTCAACACTACTTTTTTCGACCCTAGAGGAGGAGGGGATCCAATCTTATATCAACATTTATTT-

>x136_G6YZTIE01BURKI

--TCTTGCCGCTGCAATTGCTCACGCAGGAGCATCTGTAGACATAGGAACTTTTTCACTTCATTTAGCAGGTGTATCCTCAATTCTAGGAGCCGTAAATTTTATAACAACAGTCATCAATATGCGATCCTTCGGTATAACTATAGACCAAATACCTCTTTTTGTTTGAGCCGTATTTATTACCGCTATCTTACTACTTTTATCTTTACCAGTTTTAGCAGGAGCTATTACTATGCTTCTTACTGATCGTAATTTAAATACCTCCTTCTTCGATCCAGCAGGAGGTGGTGATCCCGTCCTGTACCAACACTTATTT-

>x137_G6YZTIE01BGCL2

--TTTAGCAAGGAACATTGCACATGCCGGTGCATCTGTAGATCTAGGGATCTTCTCACTCCACCTAGCAGGTGTATCTTCTATTCTAGGGGCTATTAATTTTATTACCACCGTAATAAATATACGAGCCACAGGTATAACCCCCGACCGTCTCCCATTGTTTGTATGAGCAGTATTTATTACTGCAATTCTCTTACTTCTTTCCCTCCCGGTCTTAGCTGGAGCTATTACAATACTTCTTACAGACCGAAATCTAAATACATCTTTTTTCGACCCAGCCGGAGGTGGTGACCCCATCCTTTATCAGCATCTGTTC-

>x138_G6YZTIE01B5ERS

---CTGTCTGCGGGAATTGCCCACGCTGGCGCATCAGTTGATCTTGGGATTTTTTCTCTACACCTTGCAGGTGTCTCATCAATCTTAGGAGCAGTTAACTTCATAACTACAGTAATTAATATACGAACTACAGGGATAACTATAGATCGAATACCCCTGTTCGTATGAGCCGTATTTTTAACAGCAATCTTACTGCTTCTGAGACTTCCAGTCCTAGCCGGAGCTATTACGATACTCTTAACGGACCGCAACTTAAACACAGCATTCTTTGACCCTGCTGGCGGAGGGGACCCAATCCTCTACCAACA--------

>x139_G6YZTIE01AQY1Q

--TCTGGCAGCTGCTATTGCCCACGCCGGAGCCTCAGTGGATATAGGAATCTTTTCCTTACATTTAGCAGGTGTCTCTTCTATTCTAGGAGCAGTTAATTTTATAACTACCGTAATCAACATACGCTCTTTCGGTATGACTATAGACCAAATACCCCTTTTTGTTTGAGCTGTATTTATTACAGCTATTCTTTTACTTCTATCTCTCCCTGTTCTAGCAGGAGCTATTACTATACTTCTTACTGATCGAAACCTTAATACTTCTTTTTTCG---------------------------------------------

>x140_G6YZTIE02DHD7B

--CCTTTCCGCCGCTATTGCTCATGCAGGGGCATCTGTAGACCTTGGAATTTTCTCTCTTCATCTTGCAGGAGTATCTTCCATTCTAGGCGCAGTGAACTTTATGACAACGGTTATTAACATGCGACCCCAAGGGATGACCCTTGACCGTATGCCATTGTTTGTCTGGGCGGTATTTATTACAGCAATTCTTCTCTTATTATCTCTACCAGTCTTGGCTGGTGCAATCACCATGCTATTAACAGATCGAAATTTGAATACATCTTTTTTCGACCCAGCAGGGGGAGGAGACCCCGTCCTGTACCAACACCTCTTC-

>x141_G6YZTIE02D61PY

--TTTAAGATCAACACTTGGACATTCCGGGGCTTCTGTAGATCTTGCAATTTTTTCTCTTCATTTGGCTGGAATTTCTTCTTTACTAGGTGCTGTTAATTTTATTAGGACTTTAAAAAATACACGAAGATTAGGAATAGTTTTAGATCGAATAAGATTATTTCCTTGATCCGTTTTAATTACAGCAATTTTACTTCTTCTATCACTTCCTGTATTAGCCGGAGCAATTACTATACTTCTAACAGATCGGAACTTAAACACCACTTTTTACGACCCAAGAGGAGGAGGAGATCCTGTTTTATACCAACACTTATTT-

>x142_G6YZTIE01CCULL

--ACTTTCCAGAAATATTGCGCACGCCGGTAGATCGGTAGACTTTGCAATTTTCTCACTTCACTTGGCAGGTGTAAGATCTATTCTAGGGGCAGTAAATTTTATTAGAACTTTAGGCAATCTACGGGTATTTGGGATAATCCTAGATCGAATGCCCCTATTCGCCTGAGCTGTTCTTATTACTGCAGTCCTATTGTTATTATCGCTTCCAGTTCTGGCCGGGGCCATTACAATACTGCTTACTGACCGAAACCTTAATTCATCATTCTATGATGCCAGTGGGGGAGGTGACCCAATTCTTTATCAGCATCTATTT-

>x143_G6YZTIE01A03GL

--TCTAGCGAGAGGAATCGCCCGTGCAGGAGCCTCAGTAGACATAGGAATTTTCTCTCTCCATTTAGCAGGAGTATCCTCCATCTTAGGAGCAGTAAATTTTATTACAACTGTGATTAACATACGAGCTTCAGGTATAACAATAGATCGACTACCTTTATTCGTCTGAGCTGTCTTCTTAACAGCCATTTTATTACTTCTATCTTTACCAGTTCTAGCCGGAGCTATTACCATGCTCCTAACAGACCGAAATTTAAATACATCCTTTTTTGACCCAGCTGGAGGAGGAGACCCTATTTTATATCAACATTTATTT-

>x144_G6YZTIE01BALIL

--GTTACCAGATTATTTAGCTCACTCTACTCCTGCAGTTGATCAGGCTATTTTTTCATTACATTTGGCAGGTGTTTCATCTATTTTAGGAGCTGTTAATTTTATATGCACATTTTTAAATTTGAAACCCCGA---ATAAAACTTGAACTTGCGCCTCTTTTTTCTTGGTCGGTTTATGTAACAGCATTTTTATTGATTATGTCTTTGCCTGTATTAGCAGGAGGTATTACTATGCTTTTACTAGATCGTCATTTTAACAC--------------------------------------------------------

>x145_G6YZTIE01CI2RX

---CTTTCAAGAAACATCGCTCATGCAGGCCCCTCTGTAGACCTGGCTATTTTCTCCCTTCATCTAGCAGGAGCTCCCTCCATCCTAGGCTCTATCAACTTTATTACAACAGTAATCAATATACGAAGAGAAGGAATACGCCTAGAACGAATTCCCCTTTTTGTGTGAGCAGTAAAAATTACTACAGTACTCCTTCTACTCTCCCTCCCAGTCCTTGCAGGAGCAATCACAATACTCCTCACCGATCGAAACCTAAATACAGCCTTCTTTGATCCATCAGGCGGAGGTGACCCGATCCTCTATCAACATCTATTC-

>x146_G6YZTIE02D3CEA

--TCTTGCTGCGGCTATCGCACATGCTGGAGCCTCAGTTGACCTGGGA---TTTTCTCTTCACTTGGCCGGTGTCTCGTCCATTCTTGGTGCAGTAAACTTTATAACTACTGTTATTAATATACGTTCCTTCGGAATAAGAATGGACCAGATGCCATTGTTTGTTTGATCGGTCTTTATTACAGCCATTTTACTATTGTTGTCTCTTCCTGTTCTTGCGGGAGCTATTACTATACTCTTGACTGACCGTAATCTCAACACCTCTTTCTTTGACCCAGCTGGAGGTGGTGATCCCGTCCTTTATCAACATTTATTC-

>x147_G6YZTIE01BZYSQ

--TCTAGCTAGAGGGGTCGCACATGCAGGGGCTTCTGTTGATATGGGAATTTTTTCTTTGCATTTGGCTGGTGTCTCTTCAATTTTAGGAGCAATCAATTTTATGACTACAGTTATTAATATACGATCTAAAAATTTATTTATAGATCGAATACCCCTTTTTGTATGGTCAGTTTTCATTACTGCTATTTTATTACTTTTATCATTACCTGTTTTGGCAGGGGCTATTACTATGTTATTAACTGATCGTAATTTAAA-----------------------------------------------------------

>x148_G6YZTIE01BCUQF

--GCTATCTGGAAATCTAGCTCACACTAGGGCTTCAGTTGACCTCACTATTTTTTCTCTTCACTTAGCAGGAGCCTCTTCTATCTTGGGGGCAATTAATTTTATTACAACAATTTTAAATATACGCCAAGCCGGGATATCTTTAACAAAAATTCCTTTATTTGTGTGATCAGTTTTAATTACAGCTGTTTTACTTTTACTATCTTTACCCGTTTTAGCTGGAGCAATTACAATATTACTTACAGACCGAAATTTTAACACCACATTCTTTGGTCCTTTAGGAGGGGGAGACCCAATTTTTATATCAACA-------

>x149_G6YZTIE01BK9BX

---CTAGCTAGGAACATCGCCCACGCTGGAGCGAGAGTTGATTTAGGT---TTTTCTTTACATTTAGCAGGGGTTTCCTCTATTTTAGGAGCCGTAAACTTCATAACAACTGTTATTAATATGCGCCCAGTGGGAATGCCTATAGACCGAATACCTCTGTTTGTATGAGCTGTGTTTTTAACAGCAATTTTACTTTTATTATCCCTTCCAGTCTTAGCGGGGGCTATTACTATACTACTCACAGATCGAAATCTTAATACTTCATTCTTCGACCCTGCAGGGGGAGGAGACCCCATTCTCTACCAACATTTATTT-

>x150_G6YZTIE01B85DD

---CTGTCTTCTCTTGTGGGCCATGGTGGACTATCTGTAGATTTAGTTATTTTTTCACTTCACCTTGCAGGGGTATCCTCTATTTTAGGTGCACTTAATTTTATTACTACAATTATAAATCTACGTAATCCTAATATGTTTCCTGAACAAATATCGCTTTTCTTGTGAGCAATTTTAATTACTGCTATTCTCCTTTTATTATCCTTACCAGTTCTCGCCGGGGCAATTACTATATTATTAACAGATCGTAATATAAACACAACATTTTTTGACCCTGCCGGTGGTGGTGATCCTGTTCTTTTCCAACACCTTTTT-

>x151_G6YZTIE01B3WGG

---CTAAGCCACAACTTAGCCCATGCAGGACCCTCAGTTGATTTTACTATTTTTTCCTTACATTTAGCAGGTGTGTCTTCTCTATTGGGAGCTGTTAATTTTATTAGCACAATTAATAATTTACGCTCATTTGGACTTACATTAGACCGGATGCCAATATTTGTATGGTCAGTGTTAATTACAGCAATTTTACTTTTGCTTTCATTACCAGTACTAGCCGGGGCCATTACTATATTACTTACTGATCGTAACTTAAACACGTCTTTTTATGATGCAGGGGGAGGAGGCGATCCGGTATTATACCAACACTTATTT-

>x152_G6YZTIE01BRK3C

---TTATCTTCTAACTTAGGACATTCAGGAGGAGCTGTTGATATGGGAATTTTTTCTCTTCATTTATTAGGGGTATCTTCTATTTTAGGAGCTGTTAATTTTATATCTACTGTAATTAATATACGTCCCGCTGGTATAACAATAGATCAAATACCTCTTTTTGTCTGAGCAGTCTTTATTACTGCAATTCTTCTTTTATTATCCTTACCGGTTTTAGCGGGAGGGATTACAATACTATTAACTGACCGAAATTTAAATACTTCATTCTTTGATCCTGCGGGAGGGGGAGACCC-----------------------

>x153_G6YZTIE01BAS9P

--TCAAGCCGGGAATCTTGCACACGCAGGAGCATCCGTCGACCTAACAATTTTCTCTCTCCACCTGGCCGGAATTTCCTCAATTTTAGGGGCAATCAACTTTATTACAACCATCGTCAACATAAAACCACCTGCCATCTCCCAATACCAGACCCCTCTGTTCGTGTGAGCTGTTTTAATTACTGCTGTACTTCTTCTCCTCTCACTTCCTGTCCTTGCTGCAGGAATCACAATGCTACTCACAGATCGAAACCTAAACACCACCTTCTTCGACCCTGCAGGTGGGGGAGACCCAATTCTTTATCAACATCTA----

>x154_G6YZTIE01B8NYY

--TTTAGCTGCCAATATCGCCCACGCTGGACCTTCCGTAGACTTAGCAATTTTTTCTCTACATTTAGCTGGCATCAGCTCTATTCTTGGAGCTTTAAATTTTATTACTACTATATTCAACATACGCTCTGAAGGTCTTCGCCTAGAACGTCTCCCCCTATTTGTATGAGCTGTCATAATTACGGCTGTTCTTCTCCTCCTGTCTCTCCCTGTTCTAGCCGGAGCTATCACAATATTATTAACAGATCGAAACTTAAACACTGCATTCTTTGACCCCGCCGGGGGAGGAGACCCAATTCTCTACCAACACCTATTC-

>x155_G6YZTIE02DP9M0

---TTAAGAGCCAACCTAGCGCACGCCGGGGCTTCTGTGGATTTAGCTATTTTTTCGTTACACTTAGCTGGAATTTCCTCCCTATTAGGGGCTGTTAATTTTATTTGCACTATCGGAAACTTGCGAACTCTGGGGATAGGGTATGATTTGATGCCCATATTTGTGTGAGCCGTATTAATCACTGCAATTTTGCTTCTTTTATCCCTACCTGTCTTAGCGGGGGCCATCACCATGCTTCTCACAGACCGCAACTTAAACACTTCTTTTTATGATATTGCCGGAGGGGGTGACCCAGTGCTCTACCAACATCTCTTC-

>x156_G6YZTIE01CDI8D

--CTTAGCAGCTGCTATTGCACACGCAGGTGCTTCAGTTGATTTAGGTATTTTCTCCCTCCACCTGGCCGGTGTTTCCTCTATTTTAGGAGCAGTCAATTTCATAACCACAGTTATTAACATGCGATCGTTCGGAATGAAAATAGACCAAATGCCACTTTTCGTGTGATCCGTTTTCATTACAGCCATCCTTCTTCTACTATCTCTACCTGTTCTAGCAGGTGCTATTACCATGCTACTAACTGACCGAAACCTTAATACATCTTTCTTTGATCCAGCTGGAGGAGGGGATCCAGTCCTATACCAACATCTCTTC-

>x157_G6YZTIE01BV0P3

--GTTAGTTGCTGCTATCGCCCATGCGGGAGCTTCAGTTGATATGGGAATCTTCTCACTTCACTTAGCAGGGGTTTCTCCAATTTTAGGAGCCGTAAATTTTATAACTACAGTTATTAATATACGATCTTTTGGTATAACGATAGACCAAATACCATTATTTGTATGAGCGGTATTTATTACCGCTATCCTCCTTTTATTATCTTTACCTGTTCTAGCAGGTGCCATTACTATACTCCTTACTGACCGTAATCTTAACACATCTTTCTTTGACCCATCCGGGGGAGGTGATCCTGTTCTTTATCAACACTTGTTT-

>x158_G6YZTIE01BAP38

---TTATCAGGAAACTTAGCGCATGCAGGAGCTTCCGTAGATCTAACCATTTTCTCGCTCCATCTGGCAGGAATTTCCTCAATCCTGGGGGCAATCAATTTTATCACAACCATCATTAACATGAAACCTCCCGCTATCACCCAGTACCAAACCCCTCTTTTTGTGTGAGCCGTCCTCATCACTGCTGTACTTCTCCTTCTATCCCTTCCAGTCCTAGCTGCTGGAATTACCATGCTCTTAACTGACCGTAACCTAAATACTACATTCTTTGACCCAGCAGGAGGAGGAGACCCGATCCTCTATCAACATTTA----

>x159_G6YZTIE01BA7ID

---CTAGCGGCAGGTATTGCCCACGCCGGGGCATCAGTTGATATAGGAATCTTCTCACTACATCTGGCAGGTGTATCGTCAATTCTAGGGGCCGTAAATTTCATAACCACTGTTATCAATATACGGGCCACTGGTATATCAATAGACCGAATGCCGCTTTTCGTCTGATCAGTTTTCCTTACTGCTATTTTACTTCTTCTTTCTCTACCTGTTCTCGCGGGGGCGATTACTATACTCCTAACTGACCGTAACCTAAATACCTCTTTCTTTGATCCTGCGGGGGGCGGTGATCCAGTCCTTTATCAGCACCTA----

>x160_G6YZTIE02DPVN8

--CCTAGCAGCCGCTATCGCCCATGCTGGAGCCTCAGTAGACTTAGGAATTTTCTCTCTCCATCTAGCGGGTGTATCCTCTATTCTCGGAGCTGTAAATTTCATGACCACTGTTATTAATATACGATCATTCGCTATAACTATAGACCAAATACCTCTCTTCGTCTGATCTGTATTTATTACTGCTATCCTCCTTCTTCTTTCCCTCCCAGTTCTTGCTGGCGCTATTACTATATTATTAACAGACCGTAACCTAAACACCTCATTTTTCGACCCAGCTGGAGGAGGAGACCCTGTCCTGTACCAACACTTATTC-

>x161_G6YZTIE02DHDVS

CCCCTC---GCGGCCATTGCGCACGCAGGAGCCTCAGTTGACATAGGAATCTTCTCCCTTCGCTTAGCCGGAGTCTCCTCCATTCTTGGGGCCGTAAACTTCATGACAACAGTCATTAACATACGACCAACAGGAATAACCATAGACCGCATACCTCTATTCGTCTGGTCGGTTTTTATCACAGCAGTCCTACTTCTTTTATCTCTCCCCGTCCTAGCTGGCGCTATTACCATGCTTCTAACAGACCGAAACCTTAACACTTCTTTTTTCGATCCCGCCGGAGGGGGAGACCCAATCCTATATCAACATCTATTC-

>x162_G6YZTIE01BOR5N

--ACTATCTGCTAATCTCGCCCATGCTGGGCCTTCGGTCGACTTAGCCATTTTCTCATTACACCTTGCAGGCGTTTCTTCAATTCTTGGGGCGCTAAATTTTATAACAACTGTAATCAACATGCGGATAGAAGGGCTCCGAATGGAACGGATGCCTTTATTTGTCTGAGCGGTTTTTATTACTGCTATTCTATTACTCCTCTCTTTACCAGTCCTTGCGGGAGGGATCACTATGCTTTTAACTGACCGTAATTTAAATACCAGATTTTTTGATCCTGCAGGCGGTGGGGACCCAGTTCTATTTCAACACTTATTC-

>x163_G6YZTIE02EGRXC

--TTTAGCCGCAGCTATTGCCCATGCTGGGGCCTCTGTTGATATAGGAATTTTTTCACTTCATTTAGCAGGTGTTTCACCGATTTTAGGAGCTGTAAACTTTATAACTACCGTTATTAATATACGATCATTTGGTATAACTATAGATCAAATACCTCTCTTTGTCTGAGCTGTATTTATCACTGCAATTCTCCTCCTTCTATCTTTACCTGTTCTAGCAGGTGCTATCACTATGCTTCTTACTGATCGTAATTTAAATACCTCCTTCTTTGATCCTGCCGGTGGAGGGGATCCAGTTCTTTACCAACACTTGTTC-

>x164_G6YZTIE02EOIUT

--ATTATCTGCTGCTATTGCCCACGCCGGAGCCTCAGTTGATATGGGGATTTTCTCATTACATCTTGCTGGTGTATCCTCAATTTTAGGAGCAGTTAATTTTATAACTACAGTAATTAATATACGATCCTTTGGTATAACTATGGATCAAATACCTCTTTTTGTCTGAGCTGTGTTTATTACAGCTATTTTACTCTTACTATCCCTACCCGTTTTAGCAGGAGCAATTACCATACTTCTAACAGATCGAAATCTAAATACTTCATTCTTTGATCCCGCTGGGGGAGGTGATCCCGTTCTTTACCAACACTTATTT-

>x165_G6YZTIE01BPIX8

--CCTGGCCAGAAATATGGCACATGCCGGTGGGTCAGTTGACCTAGCCATTTTCTCCCTTCACCTCGCAGGAATCTCCTCAATCCTAGGGGCAATAAACTTTATGACTACAGTCATTAATATGCGAGCCCCTGGTATACGATTTGACCGCCTTCCCTTATTTGTATGGTCTGTTTTCATCACTGTGATCCCCCTTCTTCTATCTCTACCTGTGTTAGCCGGAGCCATCACAATGCTTCTGACGGATCGAAACCTGAACACATCATTTTTCGATCCCGCCGGAGGAGGTGACCCCATTTTATACCAACACCTATTC-

>x166_G6YZTIE01CF0XT

---CTTGCTGCCGCTATTGCTCACGCCGGAGCATCTGTCGATATAGGAATCTTCTCCCTTCATTTGGCAGGGGTTTCCTCAATTCTAGGTGCTGTAAATTTTATAACTACTGTTATTAATATACGATCTTTTGGTATAACTATAGACCAGATACCTTTATTTGTATGAGCCGTATTTATTACTGCTATTCTCCTCCTACTATCTCTTCCAGTTTTAGCTGGAGCTATTACTATACTTCTTACTGATCGAAACTTAAATACATCATTCTTTGATCCTGCTGGAGGGGGAGATCCAGTTCTATACCAACACTTATTC-

>x167_G6YZTIE01AX234

--TTTGGCAGCTGCCATTGTTCATGCAGGTGCTTCAGTAGATTTAGGTATTTTCTCACTTCACCTGGCAGGTGTTTCTTCTATTCTAGGAGCAGTTAATTTTATAACAACTGTTATTAATATACGATCGTTTGGTATAAAAATGGACCAGATACCACTTTTCGTATGATCAGTCTTTATTACAGCGATTTTACTTCTATTGTCTCTACCTGTTTTAGCAGGCGCTATTACCATGTTACTAACTGATCGTAATCTTAATACTTCCTTCTTTGACCCTGCTGGGGGA---GATCCTGTTTTATATCAGCATTTATTC-

>x168_G6YZTIE01B498W

--ACTATCAAGAAATATCGCCCACTCAGGAGCTTCAGTAGATTTCGCTATTTTCTCATTACACCTCGCAGGTGCAAGGTCAATCTTGGGGGCTGTAAATTTTATCTCAACCATTGGGAATCTGCGAGCGTTTGGAATACTAATAGATTTAATACCACTGTTTTCATGGGCGGTTTTAATCACCGCTGTTCTTTTATTATTATCTCTCCCAGTACTTGCCGGCGCAATTACAATATTATTAACAGATCGAAATCTTAATTCATCGTTTTATGATGC-----------------------------------------

>x169_G6YZTIE02DRZMV

--ATTGGCGGCAGGAATCGCTCACGCAGGCGCTTCCGTAGATATAGGGATTTTCTCACTTCATCTTGCTGGTGTTTCTTCAATTTTAGGAGCAGTTAATTTTATAACAACAGTTATTAATATACGACCATCTGGAATAACAATAGACCGAATACCCCTCTTTGTATGATCAGTTTTTATTACAGCTATTTTATTATTATTATCTCTCCCTGTTCTTGCAGGAGCAATTACGATACTTTTAACTGATCGTAATCTAAATACATCTTTTTTTGATCCTGCTGGAGGTGGAGATCCTATCTTATATCAACATCTGTTT-

>x170_G6YZTIE01B0REB

--TCTGGCCGGCAATCTTGCACACGCAGGCGCATCCGTCGATCTGACAATTTTCTCCCTCCACCTAGCAGGGATTTCTTCAATCCTAGGAGCAATTAACTTCATCACAACTATTATTAACATGAAACCACCTGCCATCTCTCAGTACCAAACTCCCCTCTTCGTGTGAGCCGTTCTAATTACTGCCGTTCTTCTTCTCCTCTCCCTTCCTGTCCTTGCTGCAGGAATCACAATGCTACTAACAGATCGAAACCTAAACACTACTTTCTTCGACCCTGCAGGCGGAGGAGACCCAATTCTCTATCAACACCTA----

>x171_G6YZTIE02D0QRG

--CCTATCCGCGAGAATCGCCCATGCCGGCGCCTCTGTCGACCTAGGAATCTTCTCCCTCCATCTTGCCGGAGTGTCATCGATTCTAGGAGCAGTAAATTTCATAAGAACTGTTCTGAACATGCGGACAACTGGAATGACTATAGACCGAATACCATTGTTTGTATGAGCCGTATTCTTAACAGCAATCCTCTTGCTTCTATCTCTTCCTGTGCTGGCAGGTGCAATCACAATACTCCTTACTGACCGTAACATCAACACGTCTTTCTTCGATCCGGCAGGCGGGGGGG---------------------------

>x172_G6YZTIE01ATQFE

--TTTAGCTGCTGCAATTGCTCACGCTGGGGCATCGGTAGACATGGGAATTTTTTCTTTACATTTGGCAGGTGTTTCTTCCATTTTAGGAGCCGTAAATTTCATAACTACCGTTATTAATATACGATCCTTCGGTATGACTATGGATCAAATACCTTTATTTGTCTGAGCGGTATTTATTACTGCCATCTTACTTTTATTATCTTTACCAGTATTAGCCGGTGCTATTACAATGCTTTTAACTGACCGTAACCTAAATACATCCTTT---GACCCTGCCGGAGGTGGAGATCCTGTACTTTATCAACACTTATTC-

>x173_G6YZTIE02DFK6K

--ATTAGCTAGAAATATTGCACACTCAGGACCTTCTGTTGACCTAGCAATCTTTTCATTACATTTAGCAGGGGTATCTTCAATTTTAGGAGCATTAAATTTTATTACTACAATCATTAACATACGCCCAATAGGATTACGTATAGAACGTTTATCCCTATTTATTTGATCAGTATTTATTACAGCTATTCTTCTTCTTCTATCATTACCCGTTCTAGCAGGAGCTATTACTATATTATTAACAGACCGTAATATTAATACTGCCTTTTTTG---------------------------------------------

>x174_G6YZTIE01BJA1S

--TCTAGCGGCTGCAATCGCCCACGCCGGTGCCTCAGTAGATTTGGGTATTTTCTCTCTTCATTTAGCCGGAGTCTCTTCTATCCTAGGAGCAGTTAACTTCATAACAACCGTTATTAACATGCGATCCTTCGGTATAAAAATAGACCAGATACCACTTTTTGTATGGTCAGTTTTCATTACAGCAATCTTACTCTTATTATCCTTGCCCGTTCTAGCAGGTGCTATTACTATGCTATTAACTGACCGTAATCTTAATACTTCTTTCTTTGACCCCGCAGGAGGAGGTGATCCTGTCCTATACCAACATTTATTC-

>x175_G6YZTIE01BR568

--TTACGCCGCTGCTATCGCTCATGCTGGAGCCTCAGTTGATATAGGAATCTTTTCACTTCATCTGGCAGGTGTTTCATCTATTTTAGGAGCCGTAAATTTTATAACCACTGTTATTAATATGCGATCTTTTGGTATAACCATAGACCAAATACCTTTATTTGTTTGAGCCGTTTTTATTACCGCCATCTTACTCCTCTTATCTTTACCTGTATTAGCAGGAGCTATTACTATGCTTCTTACTGACCGAAATCTTAATACTTC-----------------------------------------------------

>x176_G6YZTIE02DZ1ML

--CTTAGCTGCTGCTATTGCCCACGCCGGTGCTTCAGTTGACATAGGAATCTTTTCTCTGCATTTAGCAGGTGTATCCTCAATTTTAGGCGCTGTAAATTTTATAACTACAGTTATCAACATACGATCTTTTGGTATATCTATAGACCAAATACCTTTATTTGTTTGAGCCGTATTTATTACAGCTATTTTATTACTCCTTTCTCTTCCTGTCCTTGCAGGAGCTATTACAATGCTTCTTACAGATCGTAACCTAAATACATCTTTTTTTGACCCTGCAGGAGGTGGGGACCCTATTCTCTATCAACATTTATTT-

>x177_G6YZTIE01A1FB7

---TTA---GCTGCTATTGCTCATGCTGGACCCTCAGTTGACATAAGAAATTTTTCTATTCATTTAGCAGGGTTTTTTTCTATTCTAGGAGCAGTTAACTTTATAACTACTGTAATTAATATACACTCATTTGGTATAACTATGGATCAAATACCTCTTTTTGTATGAGCAATTTTTATTACTGCCATCCTCCTTTTACTCTCCCTTCCAGTTTTAGCAGGTGCTATTACTATGTTGCTTACAGATTGGAATTTAAGTACATCCTTTTTTGACCCAGCCGGAGGGGGTGACCCAGTACTTTACCAGCATTTATTC-

>x178_G6YZTIE02EEA87

--CTCTGCTGGCAATTTAGCACACGCCGGGGCTTCTGTAGACCTAACAATTTTCTCTCTACACCTGGCAGGAATCTCATCAATTCTAGGGGCTATTAACTTTATCACAACCATTCTAAACATGAAACCTCCTGCAATTTCACAGTACCAAACACCATTATTTGTTTGAGCTGTATTAATTACTGCTGTCCTCTTATTATTGTCTCTTCCTGTTCTTGCTGCTGGAATCACAATACTTCTTACAGACCGAAACCTAAATACGACTTTCTTTGACCCACCTGGAGGAGGGGACCCAATTCTTTACCAACATTTA----

>x179_G6YZTIE02C7N8C

--ACTATCAAGAAGTATTGCCCACGCAGGCCCATCCGTAGATCTAGCTATCTTCTCCCTCCATCTAGCAGGAGCTTCTTCCATCCTAGGCTCTATCAACTTCATCACAACAGTAATTAATATACGAAGAGAAGGAATACGCCTTGAACGAATTCCCCTATTTGTCTGAGCAGTAAAAATCACCACAGTACTTCTCCTCCTTTCCCTTCCGGTACTCGCTGGAGCGATCACAATACTCCTCACCGATCGAAATCTAAACACAGCCTTCTTTGATCCGTCAGGAGGCGGAGACCCAATCCTCTATCAACACCTATTC-

>x180_G6YZTIE01B7Z1G

--ACTTTCAGCAGGGATCGCCCACGCGGGGGCATCAGTAGATCTTGGGATCTTTTCACTACACCTAGCGGGTGTATCGTCAATCCTAGGAGCAGTCAACTTTATAACTACAGTCATTAATATACGAGCCACTGGAATAACTATGGACCGAATTCCTTTGTTTGTTTGAGCCGTCTTCCTAACAGCCATCCTACTCCTCTTGAGGCTCCCAGTTCTAGCCGGAGCAATTACCATGCTCCTAACAGACCGAAATCTAAACACAGCATTTTTTGACCCTGCAGGAGGGGGG---CCAATTCTATATCAACACTTATTT-

>x181_G6YZTIE01AP0RD

--CCTAGCCGGGAACATGGCCCATGCTGGAGGATCTGTTGATCTTGCAATTTTCCCCCTTCATCTCGCAGGAATCTCATCGATTCTCGGATCCATAAACTTTATGACTACCGTCATAAATATGCGTGCCCCAGGAATCCGATTTGACCGTTTACCCCTATTTATTTGGTCTATATTCATCACTGTAATTCTTCTTCTTCTCTCCCTCCCAGTACTAGCCGGAGCCATCACAATGCTGTTAACAGACCGGAACCTAAACACCTCATTCTTTGACCCTGCCGGTGGAGGAGACCCAATTTTATATCAACACCTTT---

>x182_G6YZTIE01BMUDR

---TTATCAGCCGGAATTGCCCATGCTGGAGCTTCTGTAGATTTAGGAATCTTCTCTCTACACCTTGCTGGTGTATCATCTATCCTAGGTGCAGTTAACTTTATAACAACAGTTATTAATATGCGAACTACAGGGATAACTATAGACCGAATGCCTTTATTTGTATGGGCGGTTTTCTTAACAGCAATTCTACTTTTACTAAGATTACCAGTATTGGCTGGAGCTATTACTATACTTTTAACAGACCGGAATTTAAATACAGCATTTTTCGATCCAGCTGGAGGTGGAGATCCTATTTTATATCAACATTTATTT-

>x183_G6YZTIE02DO9I7

--CCTGTCTGCCGCCGTCGCACATGCCGGAGCCTCAGTAGACCTGGGAATTTTTTCACTTCATCTGGCCGGAGTCTCTTCCATCCTAGGCGCGATTAACTTTATAACTACTGTTATCAACATGCGGCCCCAAGGAATAAGGATAGACCGGATACCTCTCTTTGTGTGATCTATCTTTGTTACAACTATTTTACTTTTACTGTCTCTGCCTGTTCTAGCAGGAGCTATTACCATGCTCCTAACAGACCGAAACCTAAATACGTCTTTCTTTGACCCAGCAGGGGGAGGTGAC-------------------------

>x184_G6YZTIE01AMNKK

--TCTCGCCGCTGCGATTGCTCACGCCGGAGCTTCGGTAGATATGGGAATCTTCTCCCTTCACTTAGCGGGGGTATCTTCTATCCTTGGGGCCGTAAACTTCATAACAACCGTTATCAACATACGATCCTACGGAATAATAATAGACCAGATACCACTATTTGTATGAGCAGTTTTCATTACTGCTATTTTACTATTGTTATCTCTTCCAGTCCTAGCAGGAGCTATCACAATGCTTCTCACCGACCGTAATCTAAATACC---TTTTTCGATCCCGCAGGGGGTGGAGATCCTATTTTATACCAACACCTATTT-

>x185_G6YZTIE02D7XGU

---CTAGCAGGAATCCAAACTCACTCTGGAGGATCCGTAGACATGGCTATATTTAGTTTACATTGCGCCGGTGCATCCTCCATAATGGGTGCCATAAACTTTATAACAACTATTTTCAACATGAGAACCCCAGGAATGACTATGGATAAATTACCATTATTCGTATGATCAGTATTAATAACTGCATTCCTATTACTTTTATCTCTTCCAGTTTTAGCAGGAGCTATTACAATGTTATTAACTGATCGTAATTTCAATACAACTTTCTTTGATCCAACAGGAGGAGGCGATCCCATATTATATCAACACTTATTT-

>x186_G6YZTIE01BWV0E

--GCTTGCAGGAAATCTTGCCCATGCCGGGGCTTCTGTTGACTTAACCATCTTTTCACTCCACCTGGCTGGGGTTTCTTCAATTTTAGGAGCAATCAATTTCATTACAACAATCCTAAACATGAAACCCCCTGCCATTTCCCAGTATCAAACTCCTCTTTTTGTATGGGCTGTTCTAATTACAGCTGTTTTATTACTTCTGTCCCTACCTGTTTTAGCTGCCGGGATCACAATGCTTTTAACTGACCGAAACTTAAACACAACCTTCTTTGACCCTGCAGGAGGTGGAGACCCAATCCTCTACCAGCACCTC----

>x187_G6YZTIE02EOC3P

--CTTATCAGCGGCTATCGCCCATGCCGGAGCTTCTGTTGATTTAGGAATTTTTTCCCTTCACCTAGCAGGGATTTCATCTATTCTTGGGGCTGTAAATTTTATAACTACCGTTATTAATATGCGTTCTTTTGCAATAAGAATGGACCAAATACCCCTTTTCGTTTGATCAGTGTTTATTACTGCCATCCTACTT---CTTTCTTTACCAGTTCTTGCGGGAGCTATTACTATGCTATTGACAGACCGGAACCTTAACACCTCCTTTTTTGACCCAGCAGGAGGTGGAGACCCTGTGTTATACCAGCATTTATTC-

>x188_G6YZTIE02EROE1

--TTTATCTGGCCCTATCGCCCATGGCCCTTGTGCGGTTGATTTGGCTATTTTTTCTTTGCACTTAGCTGGTATGTCCTCTATTTTAGGTGCCATTAATTTTATTACTACTATTATTAATATACGCGCTCCGGGGATTACCTTTGAGCGTCTCAGCTTATTTGTGTGGTCTGTGTTTGTAACAGCTTTTTTGCTTTTATTAAGCTTACCAGTTTTAGCTGGTGCCATTACTATACTCTTAACGGATCGAAACTTTAATACTAGTTTTTTTGATCCTGCAGGAGGAGGAGATCCTATCTTGTACCAACACCTATTT-

>x189_G6YZTIE02DX8MI

--TTTATCAGCCGCTATCGCCCATGCAGGTGCTTCAGTAGATTTAGGTATTTTCTCATTACATCTGGCTGGTGTTTCTTCTATTTTAGGAGCTGTAAATTTCATGACTACAGTTATTAATATGCGCTCATTTGGTATAAGAATGGACCAGATGCCTTTATTTGTATGGTCAGTATTTATTACCGCTATTCTACTTTTATTATCTCTACCTGTCTTAGCGGGAGCTATTACTATACTTCTTACTGACCGAAACCTTAATACTTCCTTCTTTGATCCTGCTGGGGGAGGAGACCCGGTCCTTTACCAGCACTTGTTT-

>x190_G6YZTIE01B610P

--TTTGGCTGCAGCCATTGCTCATGCCGGAGCTTCAGTTGACATGGGTATTTTCTCCCTTCACTTAGCAGGAGTTTCTTCCATTCTAGGAGCAGTAAATTTTATAACCACCGTTATTAATATACGATCTTTCGGCATAACCATAGACCAAATACCTCTTTTCGTTTGAGCAGTATTTATTACTGCTATTTTACTTTTACTTTCTCTACCAGTTTTAGCAGGTGCTATTACTATACTTCTTACTGACCGTAATTTAAATACATCTTTCTTTGATCCAGCTGGAGGTGGAGATCCTGTACTTTATCAACACTTATTC-

>x191_G6YZTIE01AL4G0

--TCTTGCTGCTGCTATTGCTCATGCTGGAGCCTCAGTTGATATGGGTATTTTTTCTCTCCATCTAGCAGGTGTGTCTTCAATTTTAGGTGCCGTAAATTTTATAACTACCGTTATTAATATACGATCCTTTGGAATAACTATAGACCAGATACCTTTATTCGTATGAGCTGTATTTATTACTGCCATCCTCCTTTTACTTTCCTTGCCTGTTTTAGCAGGTGCTATTACTATACTTCTTACAGACCGAAATCTGAATACATCTTTCTTTGATCCTGCCGGAGGAGGTGACCCAGTTCTTTATCAACATTTATTC-

>x192_G6YZTIE01A7Q63

---CTTGCTGCCGCTATTGCTCACGCCGGAGCCTCAGTTGATATAGGAATTTTCTCTCTCCACCTGGCGGGGGTATCTTCCATCCTAGGAGCAGTAAACTTTATAACTACAGTTATTAATACACGATCTTTCGGTATAACTATGGACCAAATACCCCTTTTCGTCTGAGCAGTGTTCATCACTGCAATTCTTTTACTTCTCTCTTTACCTGTTTTAGCAGGTGCTATTACTATACTTCTAACTGACCGTAATTTAAACACATCCTTTTTTGATCCAGCCGGAGGCGGTGACCCTGTACTTTATCAACATCTGTTC-

>x193_G6YZTIE01BGIOT

--TCTATCCAGAAATATCGCCCATGCCGGTCCGTCAGTCGATATAGCGACCTTTTCTCTACACCTAGCGGGAGTCTCGTCAATTATGGGAGCACTAAACTTTATTACTACAGTAATTAATATGCGATCTAAAGGCCTACGATTAGAACGTATCCCTTTATTTGTTTGATCAGTTCTAGTCACTGCCATCCTTCTTCTACTAAGGCTCCCCGTTCTTGCAGGTGCTATCACTATACTACTTACAGACCGAAATATCAACACATCTTTTTTCGACCCTGCAGGAGGTGGAGACCCTATTTTATACCAACATTTATTT-

>x194_G6YZTIE02DQSBH

--TCTTTCTGCAGCTGTCGCCCACGCTGGGGCATCAGTGGATCTTGGAATCTTCTCGCTACACCTGGCAGGCGTATCCTCTATCCTAGGCGCAGTCAACTTCATAACAACAGTAATTAACATACGATCCACCGGAATAACTATAGACCGTATACCTTTATTTGTCTGGGCTGTATTCCTCACAGCAATCTTATTATTACTAAGGCTACCAGTCCCAGCAGGAGCAATTACAATGCTTCTAACAGACCGAAACCTAAATACAGCCTTCTTCGACCCTGCAGGGGGTGGAGACCCGATCCTGTACCAGCACCTATTC-

>x195_G6YZTIE01AL88Y

--TAAGAGGAAATTTTGGCCCCATGCTGGAGCCTCGGTCGATTTTGCTATTTTCTCTCTTCATCTAGCTGGGATTTCTTCTCTATTAGGTGCTGTTAATTTCATTAGAACTCTAGGTAACTTGCGATCATTAGGGATAGGGCTAGACACTGTTCCGCTTTTTGCTTGGGCTGTATTCATTACGGCAGTTCTACTACTTCTCTCTTTGCCCGTTTTAGCCGGAGCAATTACTATACTCTTAACTGATCGAAATTTAAACACGGCTTTTTATGATGTGGCAGGAGGAGGAGATCCTGTATTGTATCAGCACTTATTT-

>x196_G6YZTIE01BMXZW

--CCTATCTGCAGGAATTGCTCATGCCGGCGCATCAGTAGACCTAGGAATTTTTTCACTACACCTTGCTGGTGTATCTTCAATTCTAGGAGCAGTAAATTTCATAACTACAGTTATTAATATACGAACTACAGGAATAACTATGGATCGTATACCTCTATTTGTATGAGCTGTATTTCTAACAGCAATTCTACTACTCTTAAGACTTCCAGTCTTAGCAGGAGCTATTACTATACTTCTCACTGATCGAAATCTTAATACAGCATTTTTCGATCCTGCTGGAGGAGGTGACCCAATTTTATACCAACACTTATTC-

>x197_G6YZTIE02DXTFK

--CCTTGCTGCTGCAATTGCCCATGCAGGG---TCAGTAGATATAGGTATCTTCTCTCTCCACTTAGCAGGTGTTTCCTCCATTTTAGGAGCAGTAAACTTTATAACTACCGTAATTAATATACGATCTTTTGGTATAACCATAGACCAAATACCACTCTTTGTTTGAGCCGTATTTATTACCGCCATCCTGCTCCTTCTCTCTTTACCTGTATTAGCAGGAGCTATTACTATGCTTCTCACAGATCGTAATCTAAATACATCCTTCTTTGACCCTGCTGGAGGAGGAGACCCCGTTCTTTACCAACATTTATTT-

>x198_G6YZTIE02EL1DA

--TCTTGCTGCGGCTATTGCCCACGCTGGAGCCTCAGTTGACGTGGGAATTTTTTCTCTTCATTTAGCAGGCGTTTCCTCAATTCTAGGGGCTGTAAATTTTGTAACTACCGTTATTAATATACGATCGTTTGGTATAACTATAGACCAAATACCTCTTTTCGTCTGAGCTGTGTTCATCACTGCTATTCTTCTCCTACTCTCTTTACCTGTTCCAGCAGGTGCCATTACCATACTTCTTATTGATCCTAATCTAAACACCTCATTTTTTGACCCTGCTGGAGGAGGAGACCCAGTTCTTTATCAACATTTATTT-

>x199_G6YZTIE02DLX9O

---CTTAGGAGAACCCTTGGGCACTCAGGGGCGGCGGTAGACTTTGCGATTTTCTCTTTACACTTGGCTGGAATTTCTTCTTTATTAGGGGCAGTTAATTTCATTAGGACGCTTAAGAATCTGCGAACTATAGGAATGTTACTTGATCGAATAGCCATATTTCCTTGGGCCGTCTTAATCACCGCAGTTTTACTTTTGCTATCCCTGCCAGTTTTGGCGGGCGCCATTACTATACTATTAACTGATCGAAACTTAAATACTTCCTTTTATGACCCTAGAGGGGGAGGGGACCCGGTTTTATATCAGCACCTATTT-

>x200_G6YZTIE01BISQW

--TCTTTCAGGCGCGTTAGCTCATGCGGGTCCTTCTCTTGATTTTGCAATTTTTTCTCTTCATTTAGCGGGTGTAAGGTCAATTCTTGGCGCTTTAAACTTTATTACAACTGTAATTAATATGCGAATAGAAGACATAACTTGGGAACGACTACCCTTGTTTATTTGAGCTGCCTTTATTACTGTGGTTTTGTTGCTACTTGCGCTTCCTGTACTAGCAGGAGCTTTAACCATGTTGCTGACAGATCGAAATTTAAATACATCTTTTTTTGATCCTAGCGGAGGAGGAGACCCTATTCTTTATCAACACCTATTC-

>x201_G6YZTIE02DSPZW

--ACTCTCTGCTGCAATTGCTCACGCCGGAGCATCAGTAGATTTGGGTATTTTCTCTCTTCATCTTGCAGGTGTATCTTCAATTTTAGGTGCAGTTAATTTCATAACTACTGTAATTAATATACGACCTCAAGGTATAACAATAGACCGTATACCACTCTTCGTATGAGCAGTCTTCATTACCGCTATTTTATTGCTGCTTTCTCTCCCAGTATTAGCAGGAGCTATCACTATGCTTCTAACAGATCGAAACTTAAACACTTCTTTTTTTGATCCAGCTGGAGGAGGAGATCCTGTACTCTATCAACACCTATTC-

>x202_G6YZTIE02C156P

--TCTATCCTCTAATATCGCTCATAGAGGAGCCTCAGTAGACTTAGCTATTTTCTCCCTTCACTTAGCTGGAGTATCTTCTATTTTAGGCGCAGTAAATTTCATTACCACAGTAATCAATATGCGCTCAAAAGGACTGCGATTAGAGCGTGTTCCTCTTTTCGTATGATCCGTATTTATTACTGCTATCTTATTACTTCTTTCTCTCCCTGTTCTTGCCGGAGCCATTACAATGCTTCTAACTGACCGTAACCTTAATACTACTTTCTTCGACCCTGCAGGCGGGGGAGACCCTGTTCTGTACCAACACCTATTT-

>x203_G6YZTIE01AV6EH

CCTTTAAGA---GACGTAGCGCATTACGGTCCGTCTGTAGATATGACTATTTTTGCCCTCCATCTAGCAGGGGTATCTTCCCTTCTTGGGGCAGTAAACTTTATTAGTACTATTATTAGCATACGAGTGTTTTCACTTTATTTAGAACAATCTCCCTTATTTGTTTGAGCTGTATTCATCACAGCTATTCTTCTTCTCCTATCTCTACCAGTATTGGCAGGTGCTATTACTATATTACTTACAGACCGAAACTTTAATACTTCTTTTTACGAACCGAGAGGGGGAGGGGACCCTATCCTTTACCAACACTTGTT--

>x204_G6YZTIE02DBISY

--CTTGTCTAGAACCGTAGGTCAACCTGGAATGAGGGTGGACCTGGCAATTTTCAGGATACATATCGCGGGGGCTTCCTCTATTGGTGGATCCATTAACTTCCTGTGCACCATTGTAAATTTACGAAGACCGAGAGTTTCTTGGGAGAACTTGACCTTATTTATTTGAGGAGTATTCTTCACAGCTATTTTATTGGTTGTCTCCCTCCCAGTCTTTGCTGGAGGTATCACCATATTGCTTACTGACCGTAATTTCAATACCTCCTTTTTTGACCCAAATGGTGGTGGAGACCCAGTTCTCTATGCTCACCTCTTC-

>x205_G6YZTIE02D3GO6

--TCTTAGAGGAAATCTAGCACACTCAGGCCCTTCCGTTGATTGCGCAATCTTCTCTTTACACTTAGCAGGTGTGTCCTCAATTTTAGGGGCAATAAATTTTATTAGTACTATTTTTAATATACGAGCCATAGGTTTATTTTTAGATGAAATACCTTTATTTGTATGATCTGTATTAATCACAACGGTTTTACTTCTTTTAGCATTGCCAGTTTTGGCAGGAGCAATTACTATGTTATTGACTGATCGAAACCTAAATACTAGGTTTTATGACCCCGCAGGGGGAGGGGATCCTATTTTGTATCAACACTTGTTC-

>x206_G6YZTIE01BHU6G

--CCTAAGATCTAATTTGGCCCACAGAGGCGCTGCCGTTGATTTAGCAATTTTTTCTCTACATTTAGCAGGTATTTCTTCTTTATTGGGAGCAGTTAATTTTATTACGACTTTAATTAACTTGCGATCTTTAGGAATACTTGTAGAACGCATACCTTTATTCGCCTGGTCTGTTTTGGTTACGGCCGTTTTGCTACTACTTTCCTTGCCTGTCTTGGCCGGTGCCATCACTATGCTGCTAACCGATCGAAATTTAAACACTAGGTTTTATGATTCCAGAGGCGGAGGTGACCCGGTTCTTTACCAACACTTATTT-

>x207_G6YZTIE02DURE4

--CCTATCAGCGGGAGTCGCCCACTCAGGGCCTTCAGTAGACTTAGTCATTTTCTCTCTCCACCTAGCAGGG---TCCTCCATTATAGGTTCAATTAATTTCATCACCACAATTGTAGTTGCACGAAGAGCCCCCCATACTCTTCCACAAACAGCCCTATTTGTGTGATCAGTAAAAATTACAACAGTTCTCCTACTCCTTTCTCTTCCAGTATTAGCGGGAGCAATTACCATACTCCTAACAGATCGAAACGCATCAACATGCTTTTTTGACCCAAGAGGGGGAGGTGACCCAATTTTATTTCAACACTTATTC-

>x208_G6YZTIE02EC84R

--TTTAAGTAGAAATATTGCCCATGGCGGAGCCTCAGTGGACTTTGCTATTTTTTCTTTACATTTAGCTGGAGTGTCTTCACTTTTAGGGGCTGTTAATTTTATTAGAACCCTTAGAAACCTCCGACCAATAGGAATGCTAGCAGACCGGATACCATTATTCCCTTGAGCAGTTTTAATTACCGCGATCTTACTTCTTCTCTCACTTCCGGTATTAGCTGGGGCTATCACCATGCTTTTAACAGACCGAAACTTTAATTCCTCATTTTATGATCCTAGAGGCGGAGGGGACCCCTTACTCTATCAACACCTTTT--

>x209_G6YZTIE02DSG32

--CTTATCCCAAAATATCTCTCACAGGGGTGCTGCAGTTGACCTCACTATTTTTTCATTACACCTTGCCGGTGCTAGATCCATCTTAGGCGCCATTAATTTCCTCACAACAGGGGCAAATAGTCAATCCTCACCTGCAACCCCAACAAAAATTACCTTACTCACATGGTCAATTCAAATCACGGCCTTACTTCTTCTTTTATCTCTCCCTGTTCTCGCAGGAGCCATCACTATACTCCTAACTGACCGCAACTCTAACACCAGCTTCTTTGAAGTAGGTGGAGGCGGAGATCCAATCTTATATCAACATCTCTTT-

>x210_G6YZTIE01B3J1C

--TTTATCAGATTATTATTACTCCCCCTCAAGATCAATAGATTATGCTATTTTTAGTTTGCATTTAGCAGGACTATCCTCAATTGCGGGTGCTATTAATTTTATTAGGTCTTTTATGCATATTCGGCTGACAAATATAACTACTGAAAATTTTAATTTATTTAGGTGGTCTATTTATTTAACAACAATTTTGTTATTGCTTAGATTACCTGTCTTAGCTGGAGCAATTACCATATTAATTTGTGATCGTAACATCAGAACTAGATTTTTTGATCC-----------------------------------------

>x211_G6YZTIE02ETR29

---CTCTCTAATAACGTTGCCCATGAAGGCCTTTCTGTCGACCTCACCATTCTATCCCTTCATACCGCAGGATTATCCTCTCTCCTAGGATCCATTAACTTTGCCACAACAATAAAACATCTCCGCGTAAAAAGAATAACAGCAGAACTAATACCCCTCTTCGTTTGATCTATAGCCGTAACAGTCTTTTTACTGCTTTTAGCACTACCTGTGCTTGCAGGAGCCCTAACGATACTCCTTCTAGACCGGAACTTTAATACCACATTCTTCGAGCCACAGGGAGGAGGAGACCCCGTGTTATTCCAACATCTCTTC-

>x212_G6YZTIE02D85KV

---TTGAGGAGAAATATTGCGCATGCAGGGGCATCGGTGGATTTTGCTATTTTCTCTTTACATTTAGCGGGGATTTCTTCTTTGTTAGGAGCAGTAAATTTTATTAGAACCGTGGGGAATCTTCGATCATTAGGGATAGGACTAGATAATGTCTCCTTATTTGCTTGGTCAGTGTTAATTACTGCGGTTTTACTTCTTTTATCTCTACCAGTTTTAGCGGGAGCTATTACTATACTTTTAACAGATCGTAATTTAAATACAGCGTTTTATGACGTAGCGGGAGGAGGGGACCCATACGTG---------GCGTGG-

>x213_G6YZTIE02C1L9F

--TCTGGCTAGAAACATCGCCCACGCAGGCCCTTCCGTGGACCTTGCGATCTTTTCCTTACACATCGCAGGTGTTTCATCGATTTTAGGGGCTCTTAACTTTATTACCACGGTTATTAATATGCGGTATAAAGGGCTACGATTAGAGCGCGTCCCTTTATTTGTATGAGCAGCTAAGATTACGGCTATTCTCCTTCTTCTCTCTTTACCTGTTCTGGCAGGTGCTATTACAATGCTTCTCACCGACCGTAACTTAAACACTGCCTTCTTTGACCCTGCGGGAGGTGGTGACCCAGTTCTTTACCAGCACTTGTTC-

>x214_G6YZTIE02C8S3D

--CCTATCCTCTAGCCTCGCCCATGCAGGCCCGTCCGTTGATTTAGCAATTTTTTCCCTTCACCTTGCAGGAGCATCTTCTATTTTAGGCTCAATCAATTTTATCTCTACTATTATAACCGCTCGCCAAGAAGCTTACACTTTAGTCCGTATGCCCCTCTTTCTCTGAGCTGTAAAAATCACAACTATCCTCCTTCTTCTTTCCCTCCCAGTTCTTGCAGGAGCCATCACAATACTACTTACTGATCGAAATGCCAATACATCCTTCTTCGACCCAAGTGGTGGTGGAGACCCTGTTTTATTTCAGCACCTCTTC-

>x215_G6YZTIE01BMNXI

--ATTAAGAAGATCAACTTTCCATAACGGACCCTCAGTGGATCTAGTAATTTTTTCATTACATTTAGCAGGAGTTTCATCATTACTTGGAGCAGTAAATTTTATCAGAACAATCACAAACATACGGTCAACAGGAATATGAATAGAAAAA---CCACTATTCGTATGATCAGTACTAATTACAGCAATCTTATTATTACTTGCACTCCCAGTATTAGCAGGAGCAATCACAATACTATTAACAGACCGAAATTTCAACACCGCCTTTTACGAGGTAAGGGGAGGGGGAGATCCAATTTTATTTCAACATTTATTC-

>x216_G6YZTIE01BSJ4G

--TCTATCAGGAAACTTGGCTCATGGCGGCGCATCTGTAGACTTGGCT---TTTTCTCTACATCTAGCAGGAATCTCATCTATCTTAGGAGCTATTAACTTCATTACAACGGTAATTAATATGCGATGGCGAGCCATGTTATTTGAACGGTTACCATTGTTTGTGTGGTCCGTTAAAATTACAGCTATTTTGCTACTCCTGTCATTACCTGTTTTAGCTGGTGCCATTACTATACTTCTCACGGACCGAAACTTTAACACTGCTTTTTTTGACCCGGCAGGTGGCGGAGACCCTATTCTTTACCAACATCTATTC-

>x217_G6YZTIE02DQWYK

----TATCTAATAACATTGCCCACTCTGGGAGAGCTATTGATATATCTATCTTTTCCTTGCATCTGGCTGGAGCCAGCTCCATCCTAGGTGCTATTAATTTTATAACCACTATTCTAAACATACGCTCCAGCTCTATAAGTTTTGAGACCCTTCCCCTATTAATTTGATCTATTTTTATCACCACCATCCTACTTTTATTATCTTTGCCAGTACTAGCTGGCGCTATCACCATGCTATTAACTGATCGTAATTTCAACACTTCCTTCTTCGACCCAGCAGGAGGGGGAGACCCCATTTTATACCAACATCTTTTT-

>x218_G6YZTIE01BMR4T

--TCTTGCTGCTGCTATTGCCCACGCTGGAGCTTCAGTTGACATAGGGATTTTCTCTCTTCATCTGGCAGGTGCCTCTTCCATTCTAGGAGCAGTTAATTTTATAACCACGGTTATTAATATGCACTCATTTGGGATAACTATGGACCAAATACCATTGCTTGTTTGAGCTTTATTTACTACAGTCATTTTACTTCTCCTGTCCCTACCTGTTTTATCAGGTGCCATCACTATACTCCTGACCGATCGAAATCTAAACACCTCATTCCTTGACCCAGCCGGCGGTGGAAACCCTGTTTTATACCAACACTTATTC-

>x219_G6YZTIE02D28W2

---CTATCAAGTATACAAGCCCACTCAGGGGGATCGGTTGATATGGCTATATTTAGTCTTCATTTAGCCGGAATATCCTCAATACTGGGAGCCATGAATTTTATTACAACAATTATAAATATGAGAGCCCCAGGAATCTCAATGGACCGAATGCCTTTATTTGTGTGATCCGTCCTAGTAACTGCCGTATTGTTGTTGTTATCGTTGCCGGTATTGGCGGGAGCTATAACAATGCTTTTAACAGATCGGAACTTTAATACCGCGTTCTTTGATCCAGCAGGTGGAGGAGATCCTATTTTATATCAACACCTTTT--

>x220_G6YZTIE01BAW6G

--TCTATCAGCGGGAATCGCCCACGCCGGTGCTTCTGTAGACATGGGAATCTTCTCTCTCCACATTGCTGGTGCTTCTTCAATTTTAGGAGCAGTTAATTTTATTACTACTGTGATTAACATACGATCTGCGGGAATGACTATAGACCGTATCCCTCTATTCGTATGGTCAGTATTCATTACAGCCATCTTATTACTCTTATCTCTACCTGTCTTAGCAGGCGCAATCACAATGTTGTTAACAGACCGAAATCTAAATACATCATTCTTCGACCCTGCAGGAGGTGGAGATCCTATTTTATATCAACACCTTTTC-

>x221_G6YZTIE02DT08S

--TCTAAGGGGAAATATCGCCCATTCGGGACCTTCGGTGGATTTCGCCATTTTTTCCCTTCACCTAGCTGGGGTCTCTTCTTTATTAGGAGCCGTTAATTTTATTAGCACATTAAGAAATTTACGAACTCTAGGAATATTGTTAGACCGACTTCCTTTATTTGCTTGATCAGTATTAGTCACAGCTATCTTACTACTACTGTCTTTACCTGTGCTAGCAGGTGCTATCACGATACTTTTAACAGACCGAAACCTGAACACCTCATTCTATGATCCTAGAGGAGGAGGGGACCCCGTTCTGTACCAACACTTGTTT-

>x222_G6YZTIE01ASN7O

---CTTAGAAGCAATATTGGACACTCCGGAGTGTCTGTTGATTTAACTATCTTCTCGCTGCACTTGGCCGGGATTTCTTCCTTGTTGGGAGCCGTAAACTTTATTAGAACTTTGGCTAATTTGCGGGTACTCACTATAACACTAGACCGCATACCGCTTTTCCCGTGGTCGGTTTTAATCACAGCAATCCTGCTTTTGCTTTCGTTGCCAGTATTGGCAGGTGCCATCACAATGCTTTTAACAGACCGAAATTTAAACACATCATTTTATGACGCCAGGGGAGGAGGAGACCCGGTGCTATACCAGCATTTGTTT-

>x223_G6YZTIE01BK7OI

--TTTAAGAGGTAACTTGGCTCACAGGGGGGCGTCAGTTGATTTTGCTATTTTTTCTCTTCATTTGGCCGGAGTATCATCTTTGTTAGGTGCAGTAAACTTTATTAGGACTTTAATAAATCTACGAACTTGAGGCCTTTTTAGAGAGCAGATGCCCTTATTTGCTCGATCCGTGCTAGTAACGGCAATCCTATTATTACTTTCTTTACCAGTTTTGGCAGGGGCTATTACCGTACTATTAGTCGATCGTAATTTAAACAGG-------------------------------------------------------

>x224_G6YZTIE01B1O47

---TTGAGGAGAAATTTGGCTCATGCGGGCGCTTCGGTGGATCTAGCAATCTTTTCTTTACATTTAGCCGGAATTTCCTCGCTATTAGGGGCAGTTAATTTTATTTCTACTATTGTAAATTTGCGGATCTTGGGAATAAACTATGACCGTATACCTATATTTGTTTGGGCGGTTTTAGTAACTGCTGTTTTATTATTATTATCTTTACCGGTTTTAGCTGGGGCAATTACCATGTTATTAACAGACCGAAACCTGAATACATCTTTTTATGATGTGGCCGGAGGGGGGGACCCCGTTCTATACCAGCACCTATTC-

>x225_G6YZTIE01AQ594

--ACTATCAAGGAAAATTGCCCATGCTGGAGGGTCTGTAGACCTAGCAATTTTCTCCCTTCATCTTGCCGGTGCCTCATCTATTCTAGCTTCTATAAATTTTATTACAACTATTATAAATATGCGAACACCAGGAATGTCCTTTGACCGACTCCCATTGTTCGTTTGGTCCGTATTTGTGACTGCATTTCTACTACTTTTATCTCTTCCGGTACTAGCTGGAGCAATAACAATGCTTCTTACAGATCGAAATATTAACACAACATTCTTCGACCCAGCGGGAGGAGGGGACCCAATTCTATTCCAACATTTATTC-

>x226_G6YZTIE01BY8O9

--TTTAAGAGGTGCTTTAGCTCACTCAAGACCTGCAGTAGATTTAACAATTTTTTCTCTCCATTTAGCGGGTGTATCTTCTTTATTAGGTGCAGTCAATTTTATCAGAACAATTGGTAATATACGAGTGTTCGCCATGCTATTAGATCAAACTCCACTATTTGTATGAGCTGTATTGGTTACAGCAGTTTTATTATTATTATCCTTACCCGTTTTAGCAGGTGCTATCACTATGCTTCTTACAGATCGGAACCTAAATAGTTCATTTTATGAAGTTAGAGGAGGGGGAGATCCTGTTCTGTATCAGCATTTATTC-

>x227_G6YZTIE02ENY8M

--CTTAAGAAGAAACTTAGGGCACTCAGGAATAAGAGTTGACTTAACCATCTTTTCTTTGTACTTAGCCGGAATTTCATCTTTATTAGGAGCAGTGAATTTTATTAGCACATTAGCTAACTTACGGTCTATTACTATGTTTTTAGATCGCATGCCCCTTTTTCCTTGAGCTGTTTTAATTACAGCAATTTTATTATTGCTTTCTTTACCTGTGCTAGCGGGGGCTATCACTATACTTTTAACTGATCGTAACCTAAATACTTCTTTCTATGACCCAAGAGGCGGGGGGGACCCAGTTCTTTATCAACATTTATTC-

>x228_G6YZTIE02EDLZY

---CTATCTGGGTTTATCGCACATACAGGACCTGCTGTTGACTACGCCATCTTCTCTCTCCACCTAGCCGGTGCTAGGTCTATTCTAGGCGCTATTAACTTTATTACTACTATCTCCAATTTACGTCATCGAGGAATTACATTTGAGCGATTACCTTTGTTTATTTGAGCAGTATTTATTACTGTCATCCTCTTACTTTTAGCTCTTCCAGTCCTTGCAGGAGCTATTACCATACTTCTTACCGATCGAAATCTTAACACATCTTTTTTTGACCCAGCAGGAGGAGGG---CCAGTTCTATTTAGTCATCTTTTC-

>x229_G6YZTIE02EBK0E

--TCTTAGTGCTAACACGGCTCACTCTGGAGCTGCCGTTGACTTCGCTATTTATTCTCCTCACTTGGCTGGGATCTCTTCTCTCTTAGGAGCTGTGAATTTTATTAGAGCTCTAACTAATCTTCGAGTAATAGGTATACTTCTTGATCGCATGCCCTTGTTTGCTTGATCTGTTTTAGTAACCGCTATTCTTCTTCTTCTTTCTCTGCCAGTACTAGCTGGAGCTATCACTATACTCTTAACTGACCGTAACCTCAATACTTCATTTTACGACCCTAGGGGAGGGGGAGACCCTATTCTCTACCAACACTTGTTT-

>x230_G6YZTIE02DMOGD

--TCTTAGAAGAAACTCTGCACACTCTGGTTCATCTGTAGATTTTGCTATTTTTTCCCTCCACCTAGCCGGAGTCTCCTCTCTTTTGGGGGCGGTAAATTTTATTTCCACCCTTGTGAACGCACGCTGTTTAGGAATGGAGTTAGACCAAATACCGTTATTTGCTTGAGCTGTGTTTATTACAGCCGTTCTGCTTCTCCTTTCTTTACCAGTATTAGCAGGGGCTATTACAATATTACTCCTAGACCGAAATATTAACACGAGGTTTTACGATTCAAGTACAGGAGGTGACCCAGTACTCTATCAACACTTATTT-

>x231_G6YZTIE01ALWKY

--TTTGTCC---ACTCTCGGGCATCCCGGGGCTGCTGTAGATCTAGGGATTTTCTCTCTTCACCTGGCAGGGGTTAGGAGGATCCTGGGGTCGGCGAACTTTATTACTACAATCTCCAACATGCGACCTGAAGCTCTAACTATGGAACTTATACCACTATTCGTGTGATCTGTACTTTTAACCGCGATTCTACTTCTCTTGTCCTTACCGGTGCTGGCTGGGGCCATTACTATGCTATTGACCGACCGTAATTTTAATACATCTTTCTTCGACCCCGCGGGGGAAGGTGACCCAATTTTGTA--------------

>x232_G6YZTIE02DUUYV

--TTTGAGAGGGAATTTAGCGCACAGTGGAGGTTCTGTTGACTTAGCAATCTTTTCGCTTCATTTAGCGGGGGTATCCTCTTTGTTAGGTGCAGTAAATTTCATTAGCACTTTAGGGAACTTGCGAATATTTCATATGGGATTAGAAAATATCCCCTTGTTTGGTTGAGCGGTATTAATTACAGCTGTTCTATTGCTTTTGTCGTTGCCAGTCTTGGCCGGTGCTATTACTATGCTTTTAACTGATCGCAATCTTAATTCTAGGTTTTATGATGTGAGAGGAGGGGGGGATCCAGTACTTTATCAGCATTTATTT-

>x233_G6YZTIE02DVMVA

--TTTATCATCTAAAATTGCTCAAAGGGGTCCAAGAGTGGATCTAGCAATTTTCTCACTACATTTAGCGGGAGTAAGGTCTATTTTAGGTTCTATAAATTTTATAACTACAATGGTCAATGCCAAGTTACAA---GTGAGATGAGGGCATATGCCCCTATTTTTATGAGCTGTAATGGTAACAGCTTATATGTTAGTGTTATCTTTACCGGTATTGGCGGGAGGTTTAACAATGTTATTAACAGACCGTAAATTTAATACCACTTTTTTTGATCCTGGTGGAGGAGGAGACCCTATTCTTTTCCAACACATTTTT-

>x234_G6YZTIE02D0R9K

--GTTATCTGGCGCTTTAGCTCACGCAGGTGCATCGGTAGACCTTGCCATTTTCTCCCTTCATTTAGCTGGGGTAAGATCTATTCTAGGTGCACTAAACTTTATCTCGACAGTTACTAATATACGCCCTAGTATACTTTCGTGAGAGCGCGTCCCCCTTTTTGTCTGAGCTGCGTTTATCACAGTCATTTTGCTTCTTTTAGCACTGCCAGTCCTTGCCGGCGCAATTACTATGCTTTTAACAGATCGAAACTTAAACACAGCCTTTTTTGACCCCGGGGGCGGCGGAGACCCTATTCTCTTCAGCCATCTTTTT-

>x235_G6YZTIE02DW64A

--CCTAAGCAGAAACATCTCGCACGCCGGCGCGTCAGTTGATTTTGCCATCTTTTCGCTCCACCTAGCAGGCGTGTCATCGTTGCTTGGTGCGGTAAACTTTATTACCACCTTAAGCAATTTACGCGTTATAGGAATGTTAGTTGAGCGAATGCCTTTATTTCCATGAGCCGTACTAGTTACAGCTATCCTCCTCTTACTATCGCTACCTGTCCTAGCTGGAGCTATTACCATACTCTTGACTGACCGAAATTTTAATACATCATTTTACGACCCAAGCGGAGGGGGTGACCCATTGCTCTACCAGCATTTATTC-

>x236_G6YZTIE01BV11S

--TCTCTCTGGTAATAGAACTCATAGAGGGGCAAGAGTAGATTTAGCAATTTTCTCACTTCACATGGCCGGTGTCTCGTCTATTCTTGGAGCCGTT---TTTATTAGAACCTGTTTTAATATACGATCAAGTGACATTGTAATAACCAAAATATCACTTTTTGTTTGATCAGTTTTTATTACCGCTTTTCTTTTATTATTATCCCTCCCAGTTCTTGCCGGCGCTTTAACCATGCTACTCACAGACCGAAATATCAATACGTCTTTCTTCGATCCAGCAGGAGGCGGGGATCCTATTCTATTTCAACACCTTTT--

>x237_G6YZTIE02DR6QM

--TTTGGCTTCAAGCTTAGCACACAGGGGCCCTTCTGTAGATCTAGGTATTTTTTCTCTTCATTTAGCTGGAGCTTCTTCTATTTTAGGGGCGGTTAATTTCATCTCCACTATTTTAAATATACGGGTTAGAGGGATAGCTCTAGACAAAACTCCCCTATTTGTTTGATCTGTGTTTTTAACAGCAATTCTTCTTTTACTATCTTTGCCAGTGTTAGCGGGGGCTATTACAATACTTCTCACAGATCGGAACTTGAACACCACTTTTTTTGAGCCCAGAGGAGGGGGAGATCCAGTGCTGTACCAACATCTTTTT-

>x238_G6YZTIE01BB8ED

--CCTCTCCAGAAACATCGCTCATGCAGGTCCTTCTGTCGATATAGTAATTTTCTCCCTTCACCTAGCTGGGGTTAGCTCAATTTTAGCATCCATCAACTTTATTACAACAGCAAAAGAGCTCCGTATAGTA---ATAACTCTTGACCAAATACCTTTATTTGTTTGATCAGTCAAAATTACTACAATTCTACTACTTTTATCCCTTCCAGTTTTAGCAGGAGCTATTACCATACTCCTAACCGACCGTAACTTAAACACCTCCTTTTTCGACCCTTCCGGGGGAGGAGATCCCGTCCTATTCCAACACCTCTTC-

>x239_G6YZTIE02DAREJ

--TTTATCTGGCATTACCGCGCATTCTGGTGGTTCTGTGGACTTGGTGATTTTCAGCTTGCACCTTGCAGGGATTTCTTCCATTTTGGGGGCCATGAATTTTATTACCACTTTAAGTAATATGGGTGTGCCCGGCATGACCCCGGATGGGGTGCCCTTATTTGTCTGATCGATTTTAGTGACGGCCATCCTATTATTATTGTCCTTGCCAGTATTAGCGGGGGCCATAACCATGTTATTAACAGATAGGAATTTTAATACGGCTTTTTTTGACCCTGCGGGGGGTGGGGACCCGATTTTATACCAACATTTATTT-

>x240_G6YZTIE01A7UFD

---TTAGCAGCCGCTATTGCACATGCAGGGGCCTCAGTTGATTTAGGTATTTTCTCTTTACATTTAGCTGGTGTTTCTTCTATTTTAGGAGCAGTTAATTTTATAACGACTGTCATTAATATAAAACCTCCCGCCCTCTCACAATATCAAACGCCACTATTTGTATGGGCTGTATTAGTCACCGCTGTACTACTTCTTCTCTCCCTCCCCGTCCTTGCTGCAGGAATTACAATACTGTTAACCGATCGAAATCTTAACACAACCTTTTTTGATCCCACAGGAGGAGGAGACCCAATTCTTTATCAACACCTA----

>x241_G6YZTIE01B88OF

--TCTTAGAAGTAATTTAGCTCATAGAGGAGCTTCTGTGGATTTAGCAATTTTTTCTTTACATTTAGCGGGGATTTCTTCTCTATTCGGAGCAGTAAATTTTATTACTACTTTACTAAATTTGCGACTATTCAATATGGGTCTAGAGAATATCTCTCTATTTGGGTGATCTGTCTTTATTACCGCAATTTTATTACTTTTATCTCTTCCAGTTTTAGCAGGGGCTATCACCATATTGCTCACAGATCGAAATCTGAACTCCACTTTTTACGACGTAAGAGGAGGAGGAGACCCTATTTTATAC-------------

>x242_G6YZTIE01BUQ8Q

--CCTTTCTAGAAACATTGCCCACGCAGGCCCATCCGTAGATCTTGCCATTTTTTCTCTGCACCTTGCCGGAGCCTCTTCAATTCTAGGCTCCATCAACTTTATTACAACCGTGATTAATATACGTAGGGAAGGCTTACGACTTGAGCGTATCCCCCTCTTTGTATGGGCAGTAAAAATTACAACTGTTCTTCTACTCCTCTCTTTACCAGTCTTAGCCGGAGCAATTACTATGCTTCTCACTGACCGCAACCTTAATACAGCTTTCTTTGACCCTTCCGGAGGAGGAGACCCAATCCTCTATCAACATCTTTTT-

>x243_G6YZTIE01AZSII

--TTTGAGAAGAAATATTGCTCACAGGGGGGCCTCTGTTGACTTCGCAATTTTTTCTCTTCACTTAGCAGGAATTTCGTCACTACTAGGGGCAGTAAATTTTATTAGAACTCTGGGAAACCTGCGAATTTTTGGAATATTACTGGAGCGGATGCCTTTATTTGCCTGGTCAGTGCTAGTTACTGCAGTTTTGCTCCTTTTGTCTTTACCTGTACTGGCGGGGGCAATCACTATACTTCTTACAGATCGAAATTTAAATAGTTCTTTTTATGATGTGGCAGGGGGCGGAGACCCTGTTTTATACCAACATTTGTTT-

>x244_G6YZTIE02DMKUL

--ACTTTCTAGAAGAATATTTCATTCTGGTTCTTCGGTTGATTTAGCTATTTTTTCACTGCACTTAGCAGGAGTTTCATCTTTAATAGGAGCTATTAATTTTATTAGAACTCTAATAAATTTACGTGTTTTAGGAATTTTAATGGAACGAATGTCTATATTTCCATGATCAATTTTTATTACTGCTATTTTATTATTATTGTCCTTACCTGTATTAGCTGGGGCTATTACAATATTATTAACGGATCGTAACATTAATACAACTTTTTTTGATGTTAGAGGGGGTGGTGATCCAGTTTTATTTCAACATTTATTT-

>x245_G6YZTIE01BV8C4

--GCTGAGAAGGAACTTGGCTCATGCAGGGGCATCAGTAGACTTTGCCATTTTTTCTCTCCATCTAGCCGGGGTGTCTTCTTTGTTAGGGGCGGTTAACTTTATTAGAACTTTAGTGAACTTGCGGGTAATGGGAATGGTTATAGACCGAATGCCTCTATTTGCTTGAGCTGTTTTTGTAACGGCAATTTTGCTTCTGTTGTCACTACCAGTTTTAGCTGGGGCCATCACGATACTGTTAACGGATCGAAATTTAAACACTTCTTTTTACGACCCTAGAGGAGGGGGGGACCCTATTTTATACCAACATCTGTTC-

>x246_G6YZTIE02DZAO2

--TCTAGCAGGTGTTATTGCTCACGCTGGTCCAGCGGTAGATTTAGGGATTTTCTCTCTTCATTTAGCAGGGGTTTCTTCTATCTTAGGAGCAGTTAATTTCATTACTACAGTTATAAACATACGACCTCAGGGGATAACTATAGACCGCATGCCTTTATTCATTTGAGCAGTATTTTTAACTGCAATTTTACTGCTACTTTCTCTACCTGTTTTAGCAGGAGCCATCACCCTGCTTTTAACAGGCCGCAACCTAAACACCTCATTT---GACCCGGTGGAAAGGGGCGACCCTATCCTCTACCAATACTTATTC-

>x247_G6YZTIE02C9VK1

-CATTAAGAGGGAATGTTTCTCACAGAGGAGCTGCTGTTGATTTCGCTATTTTTTCTCTCCATTTAGCGGGGGTTTCTTCATTGCTTGGGGCAGTTAATTTTATTACTACAATTATTAATTTGCGAGCTTTAGGTTTATATTTGGACCGAATGCCAATGTTCCCATGAGCTGTTCTTATTACTGCTATTTTACTATTACTATCTCTTCCTGTTCTTGCAGGAGCTATTACAATACTTCTTACTGATCGTAACTTTAATTCTTCATTTTATGACCCTAGGGGAGGAGGAGATCCAGTTTTATACCAACACTTATT--

>x248_G6YZTIE02C8JA6

--TCTTGCAGGAACTTTAGCCCACGCAGGCCCCTCTGTAGACCTAGCCATTTTCTCCCTTCATCTTGCAGGAGTAAGATCTATTTTAGGATCCTTAAATTTTATTTCTACTGTAACAAACATACGACCAAGAATACTTTCTTGAGAACGAGTCCCTTTATTTGTATGGGCCGCCTTTATTACAGTTATCCTCCTTCTTCTCTCTCTACCAGTACTAGCTGGAGGCATTACTATACTTCTTACAGACCGAAATCTAAACACAACCTTTTTTGACCCAGGAGGAGGAGGTGACCCTGTTTTATTTAGGCACCTATTC-

>x249_G6YZTIE01B3K2B

---TTAAGAAATAATTTAGCCCATTCAGGCCCATCAGTCGATTTTGCTATTTTTGCGCTTCATTTGGCCGGTGTTTCCTCTCTTTTGGGGGCTGCGAATTTTATTAGGACTTTTTCTAATCTTCGAAGATTAGGAATGCTCCTGGAGCGGGTACCTCTTTTTGGGTGAGCTGTTTTAATTACAACTATTTTATTACTATTATCCCTCCCTGTCCTAGCAGGTGCTATTACTATACTTCTTACTGACCGAAACTTAAACACGGCTTTCTATGATTCAAACGGCGGAGGTGACCCAATCTTATATCAACACTTATTT-

>x250_G6YZTIE01A6LOH

--TCTAAGAAGTAATATCGCCCATAGTGGGCCTTCAGTAGATTTGGCAATTTTTTCTCTACATTTGGCAGGAGTGTCTTCACTGTTAGGTGCTGTTAATTTTATTAGAACTCTAATCAATTTGCGTGTTTTAGGGATAGTTAGAGACCGAATGCCGCTGTTTCCTTGATCGGTATTGATTACAGCGATTCTCTTGTTGCTATCTCTTCCGGTTCTTGCCGGGGCTATTACAATACTTTTAACTGATCGCAATATAAACACTAGATTTTATGATGT-----------------------------------------

>x251_G6YZTIE01BEFDS

--TTTAGCGGGTAATATTGCACATAGGGGCCCTTCAGTAGATTTGGCT---TTTTCTTTGCATTTAGCTGGAGCGTCTTCTATTTTAGGAGCAATTAACTTTATTTCCACTGTACTAAATATACGTAGGCCCGGAATAATATTAAATAGAATACCGTTGTTTGTTTGGTCTGTATTTATTACAGCTATTTTATTATTGTTATCTCTACCAGTGTTAGCAGGGGCTATTACCATGTTATTAATAGACCGAAATTTAAATACATCATTTTTTGACCCGGGGGGTGGGGGCGACCCTATTTTATATCAACATTTATTT-

>x252_G6YZTIE01AV51Y

--ACTCTCCGGCAACCTAGCACATGCCGGGGCATCAGTCGACCTCACAATTTTCTCGCTGCATTTGGCAGGAATCTCGTCTATCCTCGGGGCCATCAACTTTATCACTACAATCTTAAATATGAAGCCTCCGGCCATCTCACAATACCAAACGCCCCTCTTTGTCTGAGCGGTCCTTATCACGGCAGTACTACTCCTCCTTTCTCTCCCGGTCCTTGCCGCTGGAATCACCATGCTTCTTACGGATCGTAATCTAAACACAACCTTCCTTGACCCTGCTGGCGGAGGAGACCCCATCCTTTACCAACACCTA----

>x253_G6YZTIE02EUK89

TTTAGCAAGGGGGGT---GCCCACGCCGGAGCCTCTGTAGATATAGGAATCTTTTCCTTGCATTTAGCAGGTGTTTCTTCAATTTTAGGAGCCGTCAACTTTATAACCACAGTAATCAACATACGTCCCGCAGGAATAACGATGGACCAGATGCCTCTATTTGTATGGGCAGTCTTCCTAACCGCTATCCTACTACTGTTATCCCTCCCAGTACTAGCCGGGGCAATCACGATGCTATTAACTGACCGAAACCTTAACACTTCATTCTTCGATCCAGCGGGAGGAGGTGATCCTGTCCTCTATCAACACCTATTT-

>x254_G6YZTIE01BOCVW

--ACTTTCAGCCGGAATTGCTCACGCCGGAGCTTCAGTTGATATAGGG---TTTTCTCTTCACTTGGCAGGAGTTTCTTCAATTTTAGGAGCTGTAAATTTTATAACAACAGTTATTAATATACGAGCATCTGGAATAACTATAGACCGAATACCTTTATTTGTATGATCAGTATTTATTACTGCTTTACTACTCCTTTTATCTCTACCAGTCTTAGCAGGGGCTATTACTATATTATTAACAGATCGAAACTTAAACACTTCTTTCTTTGACCCAGCCGGGGGAGGAGACCCAATTCTTTACCAACATTTATTT-

>x255_G6YZTIE02EDNBV

--TTTATCAAGAAATATTGCGCATGCTGGAGCATCAGTTGATTTTGCT---TTTTCTTTACATTTAGCAGGAGTGAGTTCTATTTTAGGGGCAGTCAATTTTATTAGAACTTTAGGAAATTTACGAGTTTTTGGTATATTATTAGATCGAATACCTTTATTTGCTTGAGCAATTTTAATTACTGCTGTCCTATTATTATTGTCTTTACCAGTATTAGCTGGGGCTATTACAATATTATTAACAGATCGAAATCTTAATACTTCATTTTATGATGTAGGAGGAGGGAGTGATCCTATTTTGTACCAAC---------

>x256_G6YZTIE02D9CMW

--TCTTTCTAGTATTCAAGCACACTCCGGAGGTTCTGTTGATATGGTTATTTTTAGTCTTCATTTAGCTGGGGTTTCTTCTATTTTAGGTGCTATTAACTTTATTACTACAATTTTTAATATGCGAGCCCCGGGTGTGTCTTTTAATAAACTACCTTTATTTGTTTGATCTATTTTAATAACAGCTTTTTTACTACTTTTATCTTTACCTGTTTTAGCTGGTGCTATTACTATGTTGTTAACAGATAGAAACTTTAATACGACTTTTTTCGATCCAGCGGGTGGCGGGGACCCAATATTATTTCAGCATTTATTT-

>x257_G6YZTIE01BHUZT

--TCTATCAGGTCCTCAGATGCACTCAGGAGGATCTGTTGACATGGCTATATTTAGCTTACATTGTGCGGGTGCCTCATCAATAATGGGTGCAATAAACTTTATTACAACTATATTCAATATGAGAGCACCGGGTATGACTTTCGACAAATTACCACTATTTGTTTGGTCAGTTTTAATAACTGCAGTTCTATTACTACTATCTTTGCCTGTACTGGCTGGAGCCATAACAATGCTCCTAACAGACAGAGATTTTAATACTACTTTCTTCGACCCTGCTGGAGGTGGAGACCCAGTTCTATATCAACATCTATTC-

>x258_G6YZTIE01BKIG9

--TCTTAGCCATTCCATTTTTCACTCGGGAATGTCAGTAGATTGCTGTATTTTTTCATTGCACCTAGCCGGGGTTTCTTCGATCTTAGGGGCGATAAATTTTATTACTACAATTCTTAACATACGGATTACTGGGATAGATTTAGACCTTGTCCCATTATTCCCATGATCAGTTCTAGTAACTACTGTTTTATTGCTATTGGCACTTCCCGTTTTGGCGGGGGCCATTACTATACTTCTCACTGACCGGAACCTAAATACGGGGTTCTATGACTCAAGAAGAGGGGGAGACCCTGTTCTTTACCAACACTTGTTT-

>x259_G6YZTIE01AL9FE

--TCTAGCCTCAAATATTGCTCATGCTGCCCCC---GTTGATTTAGCTATCTTTTCTCTTCATCTAGCAGGAGTATCTTCTATCCTTGGAGCAGTAAACTTTATTACCACAGTTATTAATATACGACCCAAATTAATACATTTAGAACGGGTGCCATTATTTGTATGATCAGTAAAAATTACCGCCATCCTTCTTCTTCTATCTCTGCCTGTATTAGCAGGAGCAATTACTATACTTCTAACAGACCGAAACTTAAATACTGCATTTTTTGACCCAGCAGGAGGTGGTGATCCTATTTTATACCAACACCTATTC-

>x260_G6YZTIE01AVJQO

--TTTATCCGCAGCTATTGCCCATGCAGGTGCTTCAGTAGACTTAGGTATTTTTTCACTTCATTTAGCTGGAGTATCTTCTATTTTAGGTGCTATTAACTTTATAACTACTGTAATTAATATGCGTCCCCAGGGTATAAGAATAGACCGTATACCTTTATTTGTTTGATCGGTCTTTATTACAGCTATTTTACTTTTACTATCTCTTCCTGTCTTGGCAGGTGCCATTACAATATTATTAACTGATCGTAATTTAAATACATCTTTCTTTGACCCAGCAGGGGGTGGGGATCCTGTATTGTATCAACACTTATTT-

>x261_G6YZTIE01AR347

----TTTCCAGAAATATCGCGCATTCAGGAGCGTCTGTGGATTTAACTATCTTTTCCCTCCATTTGGCGGGG---TCATCTACCCTTGGTGCTATTAACTTTATGTCTACGGTGATCAACATACGGCCAGCCACGATAACATTCGACCGAATTCCCCTATTCGTTTGGAGAGTTTTTTTAACGGTAATCCTTCTACTTTTATCCCTCCCGGTTCTAGCAGGAGCAATCACTATACTTCTGACAGACCGAAACCTTAACACCTCCTTCTTTGACCCTACTGGAGGTGGAGACCCTATCCTTTATCAGCATCTCTTT-

>x262_G6YZTIE02EIAU7

--TTTAGCCGCTGCCATTGCCCACGCCGGAGCGTCAGTAGATATAGGAATTTTTTCTCTTCATTTAGCAGGGGTCTCTTCAATTCTAGGGGCCGTAAATTTTATAACAACAGTTATTAATATACGATCGTTTGGAATGACTATAGACCAAATGCCTCTTTTTGTATGAGCTGTATTTATTACTGCTATTCTACTACTTTTGTCTTTACCAGTTCTAGCAGGAGCTATTACTATACTTTTAACAGACCGAAACCTAAATACATCATTTTTTGATCCAGCCGGTGGAGGGGACCCTGTTTTGTATCAACACCTATTT-

>x263_G6YZTIE02EKJR9

--CCTTGCAAGAGGAATTGCCCACACTGGTACCTCAGTAGATATAGGAATCTTTTCTTTACACTTAGCAGGAGTGTCATCAATTCTAGGAGCGGTAAATTTTATAACTGCAGTTATTGATATATGAGCAGCCGGCATAACCATATACCAAATACCATTATTCGTGTGAGCTGTGTTCTTGACAGCAATCTTACTACTTCTCTCCCTCCCAGTACTAGCCGGAGCTATCACTATACTACTAACTGACCGAAATCTAAACACA---TTCTTTGATCCAGCAGGAGGTGGAGATCCAGTCCTATACCAACACTTATTT-

>x264_G6YZTIE02DSZCG

---CTTAGGAGAAATATTGCGCACAGGGGCGCCTCGGTAGATTTTGCTATTTTTTCCTTACACTTAGCAGGAATTTCCTCACTATTAGGGGCTGTAAATTTTATTAGAACTCTAGGTAATTTACGTATTTTTGGTATGTTGCTTGAACGGATGCCGCTGTTTGCCTGATCTGTCTTAGTTACTGCTGTTTTGTTGCTTTTATCTCTCCCGGTGTTAGCGGGGGCTATTACAATGCTTCTGACGGATCGTAATTTAAACAGGTCTTTTTATGATGTAGCAGGAGGGGGGGATCCCGTTCTTTATCAACATCTGTTT-

>x265_G6YZTIE01A7SOP

--GCTACCGGGGAATGTAGCCCATGCAGGGCCCGCGGTAGATTTAACTATTTTATCACTTCATTTAGCCGGTGTTTCTTCACTTCTCGGTGCAATTAATTTTACAACTACTATCATGAACAGACGAATAGAAGGAATGCCTTCAGAAAAAATACCTTTATTTATCTGGTCCGTCCTAGTCACTGTTGGGCTTTTAATCTTAGCCCTTCCTGTTTTAGCAGGGGCATTAACAATATTAATTTTAGACCGTAACTGTAATACCTCATTCTTTGAGCCAACAGGGGGAGGTGACCCAATTCTATTTCAACACCTCTTC-

>x266_G6YZTIE01CD4CO

--TCTGCCTGCCGGTATTGCTCACACTGGAGCATCTGTAGACATAGGAATCTTCTCGCTCCATCTTGCAGGGGTATCGTCAATCTTAAGAGCGGTAAACTTCATAACTACCTTAAGTTACATACGAGCCACTGGAATGTCAATGGTTCACATGCCTCTTTTCGTCTGGTCAGTGTTCCTTACTGCCATTCTTCTCCTTCTTTCCCTCCCTGTTCTTGCCGGAGTGATCACCATGCTCCTAACGGATCGTAATCTAAACAGCTCTTTCTTTGACCCTGCAGGTGGAGGTGACCCTGTTCTCTACCAACATTTATTC-

>x267_G6YZTIE01AO4WU

--CCTTTCTAGAAATATTACACACGCAGGCCCATCAGTAGATTTAGCCATTTTTTCTCTTCACCTAGCAGGTGCCTCATCCATCTTAGGATCAATCAACTTTATTGCCACAGTAATTAATATACGAATGAAAGATATTCGCCTAGAACGAATTCCCCTATTTGTTTGGGCAGCGGTTATCACAACTGTTCTTCTACTCCTATCTCTGCCCGTCCTAGCTGGAGCAATTACAATACTTCTCACAGACCGAAATCTCAACACAACCTTCTTCGACCCAGGAGGCGGAGGAGACCCCGTTTTATTCCAACACCTGTTC-

>x268_G6YZTIE02EUKCO

--ACTAGCCGGGAATTTGGCCCATGCAGGAGCTTCCGTCGACTTAACAATCTTTTCGTTACACCTTGCAGGGATCTCTTCGATCCTAGGAGCTATTAATTTTATCACAACAATTATTAATATAAAACCCCCTGCTATTTCGCAATATCAGACACCTCTATTTGTGTGGGCTGTTCTAATTACCGCGGTTCTTCTGTTATTGTCCCTTCCCGTTCTTGCTGCTGGGATCACGATGCTCCTGACAGATCGTAACCTAAATACCACATTTTTTGACCCCGCAGGCGGAGGTGACCCAATTCTTTATCAACACTTA----

>x269_G6YZTIE01BYTK6

--CCTTTCAAGAAATTTAGCCCATATAGGACCATCTGTTGACCTAGCTATTTTTTCTCTTCATTTAGCAGGAATTTCATCAATTCTAGGAGCTATTAATTTTATTACTACCATTATTAATATACGATGAGAAGGTATACTAATAGAACGACTTCCTCTATTTGTATGATCTGTATTAATTACTGCAGTTCTCTTACTACTATCCCTCCCAGTTCTTGCAGGTGCAATTACTATATTATTAACCGACCGAAATTTTAATACTACATTCTTTGATCCAAGAGGTGGAGGAGACCCAATCTTATATCAACATCTATTC-

>x270_G6YZTIE01BUXGF

--ATTGGCAAGTAACATTGCTCACTCAGGGGCCTCAGTAGATCTAACAATTTTTTCTTTGCACTTAGCAGGAGCTTCGTCAATTCTAGGAGCAATTAATTTTATATCAACAGTAATTAACATACGAGGAAAAAGACTAACTTTTGATCGTTTACCTTTATTTGTGTGAAGAGTATTTGTAACTGTAATTTTATTACTATTGTCGCCTCCGGTATTAGCAGGAGCTATTACAATATTGCTTACTGACCGAAATTTAAATACATCATTTTTTGATCCGACAGGAGGAGGAGATCCTATTCTATACCAACATTTATTC-

>x271_G6YZTIE01CGLPX

--CCTATCTGCTGCCATCGCTCATGCTGGGGCCTCAGTAGACCTGGGTATTTTTTCCCTACACTTAGCAGGTGTATCATCCATCCTAGGGGCTGTTAATTTCATAACAACAGTTATTAACATACGATCTTTCGGCATGAAGATAGACCAAATACCACTTTTCGTGTGGTCAGTCTTCATTACAGCCATTTTACTATTACTATCTCTTCCTGTTTTAGCTGGAGCAATTACTATGCTATTAACTGATCGTAATCTAAACACCTCTTTCTTCGATCCAGCCGGAGGGGGAGACCCTGTGCTATATCAACACTTATTC-

>x272_G6YZTIE01CFVTW

---TTTAGCCGCGCTATTGCCCACGCAGGAGCTTCAGTTGACATGGGTGTTTTCTCACTCCACCTGGCCGGAGTCTCGTCCATTCTTGGGGCCGTAAATTTTATAACTACCGTTATTAATATACGATCATATGGAATATCAATAGATCAAATACCCCTGTTTGTATGAGCTGTATTTATCACGGCAATTTTACTACTTTTATCCCTGCCTGTATTAGCTGGAGCCATTACAATGCTTCTAACAGATCGTAATTTGAACACATCCTTTTTTGACCCTGCCGGAGGGGGAGACCCAGTGCTCTACCAACACTTATTT-

>x273_G6YZTIE01BYSAB

--TTTATCCAGTATTCAAACCCATTCTGGGGGATCTGTGGATATGGTGATATTTAGTCTTCATTTAGCAGGGATATCTTCTATATTGGGTGCTATGAATTTTATAACAACAATATTTAATATGAGGGCACCGGGCATAACTATGGATAGAATGCCTCTATTTGTTTGATCTGTTTTAGTAACTGCTTTTTTATTATTATTATCGTTGCCGGTATTAGCCGGTGCTATAACAATGCTTTTAACCGATCGAAATTTTAATACTACTTTTTTTG---------------------------------------------

>x274_G6YZTIE02D0574

--ATTAGCTGGAATACAATCACATTCAGGGGGATCGGTAGATATGGCGATATTTAGTCTTCATTTAGCAGGGGCTTCATCAATACTTGGGGCAATAAATTTTATTACAACTATATTTAATATGCGAGCTCCAGGAATAACAATGGATCGACTTCCATTATTTGTTTGATCAATCTTAATAACTGTATTTTTACTATTATTATCTTTACCAGTTTTAGCAGGTGCTATAACTATGTTATTGACTGACCGAAATTTTAATACAACCTTTTTTGACCCAGCTGGAGGTGGTGATCCTATATTGTATCAACACTTATTC-

>x275_G6YZTIE02ER0KU

--TTTAGCTGCAGCTATCGCCCACGCTGGTGCTTCAGTTGACATAGGAATTTTCTCTTTACACTTAGCGGGTGTATCCTCAATCTTAGGTGCTGTAAATTTTATAACTACAGTTATTAATATACGATCTTTTGGCATATCTATAGACCAAATACCTTTATTTGTTTGAGCTGTCTTTATTACAGCGATTTTGTTACTTCTTTCTCTCCCTGTTCTCGCAGGAGCTATTACAATGCTCCTTACAGACCGTAACCTAAATACATCTTTCTTTGACCCTGCAGGAGGTGGAGATCCTATCCTATATCAACACTTATTT-

>x276_G6YZTIE01AMYQU

--GCTAGCAGGTAATCTCGCCCATGCAGGGGCCTCCGTTGACCTCACAATCTTTTCACTGCATTTAGCAGGAATTTCATCAATTTTAGGGGCAATCAACTTTATTACAACCATCGTTAACATGAAACCTCCCGCCATCTCACAATACCAAACCCCCCTGTTTGTGTGAGCCGTCCTAATCACAGCAGTTCTGCTTCTTCTCTCCCTGCCTGTCCTTGCTGCCGGAATCACCATGCTCCTCACAGACCGAAACCTTAATACAACTTTCTTTGATCCCGCTGGAGGGGGAGACCCCATCCTGTACCAACACTTG----

>x277_G6YZTIE02DUA3W

---CTTGCTGCCGCAATTGCTTACGCCGGGGCTTCAGTTGACATGGGCATTTTTTCGCTCCACTTGGCAGGTGTATCCTCAATTCTTGGTGCCGTAAACTTTATAACAACTGTAATTAATATACGATCTTTTGGCATAACTATAGACCAGATACCTTTGTTTGTCTGAGCTGTCTTTATTACTGCCATCCTCCTTCTATTATCTCTACCTGTTCTAGCAGGAGCCATTACAATACTTCTTACCGACCGAAACCTCAATACATCATTTTTTGATCCAGCAGGAGGTGGTGACCCAGTCCTGTACCAACACTTATTC-

>x278_G6YZTIE02DS5MV

--TTTGTCCGCAGCCATTGCCCACGCAGGGGCTTCTGTAGATATAGGTATCTTCTCTTTACATTTAGCAGGTGTTTCTTCTATATTGGGGGCAGTAAATTTTTTAACTACAGTAATTAATATACGACCTAATGGTATAACAATAGACCGAATACCTTTATTTGTATGATCTGTTTTTATTACAGCAGTATTATTACTTCTCTCTCTTCCTGTGCTAGCAGGGGCTATCACTATACTGTTAACTGATCGAAATTTAAACACTTCTTTCTTTGATCCTGCAGGAGGGGGAGACCCTATTTTATACCAGCACCTG----

>x279_G6YZTIE01AZJKJ

--ATTAAGAAGAAATATTGCGCATGCTGGAGCATCTGTAGATTTCGCAATTTTCTCTTTACATTTAGCCGGGATCTCTTCTTTATTAGGGGCTGTAAATTTTATTAGAACCTTGGGTAACCTGCGCTCTCTGGGGATAAGATCAGATACAGTTCCTTTGTTTGCTTGAGCTGTTTTTATTACTGCAGTGTTACTTCTTTTATCGCTGCCGGTTCTTGCAGGGGCAATTACTATACTGTTGACGGATCGTAATTTGAACACTGCCTTTTTTGATGTGGCGGGAGGGGG-----------------------------

>x280_G6YZTIE01BMJP1

--ATTAAGTTCAATACAAAGTCATTCCGGTGCATCTATCGATTTAGCTATTTTTAGTTTGCACTTAGCAGGAGCTTCTTCTATTTTAGGGGCAATCGATTTTATTTCTACGATTATAAATATGCGTAATCCAGGTCAAACTTTATATAGAATACCGTTGTTTGTGTGATCTATATTCGTAACCGCATTTTTATTATTATTAGCTGTTCCCGTATTAGCTGGCGCTATTACTATGTTATTAACAGATCGTAATTTTAATACATCATTTTTTGATCCATCTGGTGGAGGCGATCCTGTTTTATATCAACACTTATTT-

>x281_G6YZTIE01CBZSS

--TTTATCTGCGGCAATTGCACATGCCGGGGCATCAGTAGATCTAGGTATTTTTTCTCTCCATTTAGCGGGTGTATCATCTATTTTAGGGGCTATTAACTTTATAACAACTGTAATTAATATGCGCCCTCAGGGAATGAGAATAGATCGAATGCCATTGTTTGTTTGATCGGTTTTTATCACTGCTATTTTACTATTATTGTCTTTACCAGTGTTAGCAGGAGCCATCACTATATTACTAACAGATCGAAATTTAAATACTTCTTTCTTTGACCCAGCGGGCGGCGGCGACCCAGTACTTTATCAGCACCTGTTT-

>x282_G6YZTIE01CE5X9

--TCTGGCAGCTGCTATTGCTCACGCAGGAGCTTCAGTAGATTTAGGTATTTTTTCTTTACATTTAGCAGGAATTCCATCCATTCTTGGTGCTGTTAATTTTATAACTACTGTTATTAACATACGCTCTTATGGTATAACAATAGACCAAATGCCATTATTTGTTTGAGCTGTATTTATTACGGCTGTTTTACTTCTACTTTCTTTACCTGTTTTAGCAGGAGCTATTACTATACTACTTACAGATCGAAATCTTAATACATCATTTTTTGATCCTGCAGGAGGAGGGGACCCAGTCCTTTACCAACATTTATTC-

>x283_G6YZTIE02D4LPF

--GCTCGCTGGCAACCTTGCCCACGCAGGGGCCTCTGTTGACTTAACAATCTTCTCTCTACACCTAGCAGGAATTTCTTCAATTCTTGGAGCCATCAATTTCATTACTACCATTATTAACATAAAACCCCCAGCTATAACCCAATATCAGACTCCACTTTTCGTGTGATCTGTCCTAATCACCGCCGTCTTGCTCCTCCTATCCCTCCCTGTTCTTGCCGCCGGAATTACAATGCTACTTACAGACCGAAACTTAAATACAACTTTCTTTGACCCGGCAGGAGGAGGAGACCCTATTCTTTACCAACACCTG----

>x284_G6YZTIE02EW7XL

--TCTAGCCGGTAATCTAGCACATGCGGGGGCATCTGTAGACCTCACTATTTTCTCCCTACATCTCGCAGGGATTTCCTCAATTCTTGGGGCTATCAACTTTATTACAACAATTATTAACATGAAACCCCCAGCCATCTCCCAATACCAGACACCTCTATTCGTATGAGCTGTGCTAATTACCGCCGTTCTACTCCTTCTCTCACTTCCTGTCCTTGCTGCCGGAATTACAATGCTACTTACAGATCGCAACCTAAACACCACCTTCTTCGACCCTGCAGGCGGAGGGGACCCTATCCTGTATCAGCACCTG----

>x285_G6YZTIE02DG8SV

--CTTAGCAGCCGCTATCGCCCACGCAGGAGCCTCAGTTGATTTAAGTATTTTTTCATTACACCTTGCTGGTGTATCTTCAATTCTAGGGGCAGTTAATTTTATAACGACCGTCATTAATATGCGATCCTTTGGAATAAAAATAGATCAAATACCACTATTTGTGTGATCAGTTTTTATTACTGCAATTCTACTTTTGTTATCTTTACCAGTATTAGCAGGAGCTATTACCATGTTATTAACTGATCGTAACCTTAACACTTCATTCTTTGATCCTGCCGGAGGAGGAGACCCTGTCTTATATCAACATTTATTT-

>x286_G6YZTIE02DI82N

--CCTATCTAGGAATATTGCCCATGCCGGCCCATCTGTAGACCTTGCCATCTTTTCCCTACATTTAGCAGGGGCCTCCTCAATTCTAGGCTCCATTAACTTTATCACAACCGTAATTAACATACGTAGAGAAGGGTTACGACTTGAACGAATTCCTCTTTTTGTTTGAGCTGTAAAAATCACAACCGTCCTTCTCCTTCTCTCCTTACCTGTATTAGCAGGCGCTATTACTATACTTCTTACTGACCGCAACCTTAACACTGCATTCTTCGACCCTTCCGGAGGAGGAGATCCAATTCTATATCAACATCTCTTT-

>x287_G6YZTIE01BYQR2

--TCTCTCAGCAGCTATTGCTCATGCGGGGGCGTCGGTTGATTTGGGGATTTTTTCTCTTCATTTGGCAGGAGTGTCTTCTATTTTAGGAGCAATTAATTTTATGACAACAGTGATTAATATACGGCCTCAAGGTATAACAATAGACCGGATGCCTTTGTTTGTATGATCTGTTTTTATTACTGCGATTTTATTGTTACTTTCTCTGCCCGTATTGGCAGGTGCTATTACTATATTATTAACTGATCGTAATTTAAATACTTCCTTT---GACCCTGCTGGGGGAGGGGATCCTGTATTATATCAAC---------

>x288_G6YZTIE02DVP9G

--TCTAGCAGCCGCTATTGCACACGCAGGTGCCTCAGTTGATTTGGGTATTTTTTCCCTTCATCTTGCCGGTGTTTCCTCGATTTTAGGTGCGGTAAATTTTATAACTACAGTGATTAATATACGACCTCAAGGAATAACAATAGACCGTATACCTTTATTTGTATGAGCAGTATTTATTACAGCGATTTTACTCCTCCTATCATTACCAGTTCTAGCAGGAGCTATTACTATACTCCTTACAGATCGAAATCTTAATACATCCTTT---GATCCAGCAGGAGGTGGAGACCCAGTTCTTTACCAACACTTATTT-

>x289_G6YZTIE01B2F65

--TCTCGCTGCTAACATTGCCCATGCCGGTGCTTCAGTAGACTTAGGAATCTTTTCACTTCACTTAGCTGGGGTTTCTTCAATTCTAGGAGCAGTTAATTTTATAACAACAGTTATTAATATACGAGCAAGAGGAATAACAATAGATCGAATACCTCTTTTTGTATGATCAGTATTTATTACAGCAATTCTTCTTCTTCTTTCCCTACCAGTCTTAGCAGGAGCTATTACTATACTATTAACTGACCGTAATTTAAATACTTCATTCTTTGACCCAGCTGGAGGGGGAGATCCTATTTTATATCAGCACCTA----

>x290_G6YZTIE02D7JSC

--ACTATCCGGAAACCTCGCCCACGCAGGAGCATCCGTAGATTTAACCATTTTCTCCCTTCATCTAGCAGGGATTTCATCAATTCTAGGAGCTATTAACTTTATTACTACTATTATTAATATAAAACCCCCTGCTATTTCTCAGTACCAAACGCCGCTATTTGTTTGGGCCGTCCTAATTACAGCCGTACTTCTTTTACTCTCTCTCCCTGTCCTGGCTGCCGGTATTACCATGCTTCTGACTGATCGAAACCTTAATACTACCTTCTTTGACCCTGCCGGAGGGGGGGACCCTATCCTTTACCAACACTTA----

>x291_G6YZTIE02DV8KO

--CCTAGCCGGCAACCTAGCCCATGCAGGAGCATCCGTTGACTTAACTATTTTCTCCCTTCACCTTGCAGGGATTTCATCCATCCTCGGTGCAATCAACTTTATCACAACCATTATTAATATGAAACCCCCAGCTATCTCCCAATATCAAACACCCCTTTTCGTATGAGCAGTTCTAATTACTGCTGTTCTCCTACTCTTGTCCCTCCCTGTTTTAGCTGCCGGTATTACAATGCTCTTAACAGACCGTAACCTTAACACGACTTTCTTTG---------------------------------------------

>x292_G6YZTIE01B98P5

--ACTATCCGGAAATCTTGCCCATGCTGGGGCCTCTGTTGATCTTACAATCTTTTCCCTACACTTAGCTGGTATTTCATCAATTTTAGGTGCAATCAACTTTATTACAACCATCCTTAATATGAAACCTCTTGCAATCTCCCAATACCAAACCCCTTTATTTGTTTGGGCTGTCCTAATTACAGCTGTTCTCCTACTTCTGTCCCTCCCTGTTCTTGCGGCTGGCATTACAATGCTACTCACAGATCGAAACCTAAATACAACCTTCTTTGATCCTTCAGGAGGGGGAGACCCAATTCTGTACCAACACCTC----

>x293_G6YZTIE01B24E2

--CCTCGCAGGCGACCTGGCCCATGCAGGAGCCTCTGTCGACCTAACAATCTTTTCCCTTCACCTGGCTGGGATTTCATCGATCCTTGGGGCTATCAATTTTATTACCACAATTATTAATATGAAACCCCCTGCCATCACTCAGTACCAAACTCCCCTATTCGTGTGAGCAGTCCTAATTACAGCCGTTCTCCTTCTTCTCTCCCTGCCTGTCCTAGCCGCTGGAATTACAATGCTACTCACAGACCGAAACCTAAACCCAACCTTCTTCGACCCGGCAGGGGGAGGGGACCCCATTTTATATCAACACTTA----

>x294_G6YZTIE01BFTVZ

--TCTCTCAGGAAACCTGGCACACGCAGGTGCATCCGTCGACTTAACTATCTTTTCACTTCACCTAGCCGGAATTTCATCAATTTTAGGGGCAATCAACTTTATCACAACAATTCTCAACATGAAGCCCCCAGCCATCTCACAGTACCAGACACCCCTCTTCGTCTGAGCCGTGCTAATTACAGCTGTACTGCTTCTTCTCTCCCTCCCTGTACTCGCCGCCGGAATTACAATACTCCTAACAGACCGAAACCTCAATACAACTTTCTTTGATCCCGCAGGAGGAGGGGACCCTATCTTATACCAACACTTA----

>x295_G6YZTIE02EAJTR

--ACTCTCTAACGGTATTGCCCATGCAGGACCATCAGTTGACACAGCAATTTTTTCACTCCATTTAGCAGGAGCATCTTCAATTATAGGAGCCATTAACTTTATGTGCACAGTAGTTAATATATGCCCAAAAGGTATATCAATAACATGTATACCTCTATTTGTCTGAGCAGTATTTTTAACCGCTATTCTCCTTCTTCTATCTCCACCCATATTAGCAGGTGCCATCACCATACTACTAACTGACCGTAATCTAAACACTTCTTTTTTCG---------------------------------------------

>x296_G6YZTIE02EAJG9

--ATTAGCTGCAGCTATTGCGCATGCTGGGGCTTCTGTAGATCTGGGTATTTTTTCTCTTCAATTAGCGGGGGTCTCTTCTATTTTAGGAGCTGTAAACTTTATAACTACCGTTATTAATATACGACCTAAGGGGATAACAATAGACCGTATGCCTTTATTTGTTTGATCTGTTTTTATTACTGCTATTCTATTACTATTATCACTTCCTGTGTTGGCAGGCGCTATTACTATACTTTTAACTGATCGAAATCTTAATACTTCTTTCTTTGATCCTGTCGGAGGGGGTGATCCAGTGCTCTATCAGCATTTGTTT-

>x297_G6YZTIE02C2G7M

--TTTATCAGGCCCATTAGCTCATTCAGGTCCTTCAGTGGATTTAGCTATATTTAGTCTACATATAGCCGGTGCTTCCTCAATAATGGGAGCTTTAAACTTTATAACAACTATAATAAACATGAGAGCTAAAGGAATGTCTTTTGTAAAACTTCCTTTGTTTGTTTGATCATTATTTATCACAGCATTTTTATTGTTATTATCTTTACCTGTCTTAGCAGGTGCCATAACCATGCTTTTGTTTGACAGAAACTTTAACACAAATTTCTTTGATCCTTGCGGAGGAGGTGACCCT---CTTTATCAACACTTATTC-

>x298_G6YZTIE02DE2TY

--TTTATCAGGCCCTCAAACCCATTCTGGTGGATCAGTAGATCTGGCCATTTTTAGTTTACACACAGCAGGAACTTCCTCTATTATGGGTGCTATAAACTTTATAACAACTATATTTAACATGAGGGCTCCTGGTATGTCTTTTGATAAATTACCGCTGTTCGTATGGTCAGTATTAATTACCGCTTTCTTGTTATTACTTTCCCTACCTGTATTAGCAGGAGCTATAACCATGCTTTTAACCGATAGGAATTTTAATACTAGTTTTTTTGATCCTGCAGGTGGTGGTGATCCAGTTTTATACCAACACTTGTTT-

>x299_G6YZTIE01BR7QC

--TCTATCTAGAAATGTGGCTCATTCAGGTCCTTCTGTAGATCTTGCTATTTTTTCTTTACATTTGGCTGGTGTGTCTTCAATTTTGGCTTCTATTAATTTTATTACTACTTCTATGAATATGCGTTCTGCTGGATTGCGATTGGAACGTGTTCCTTTATTTGTTTGATCTGTTGCTATTACTGCTTTATTGCTTTTATTGTCTTTACCTGTTTTAGCTGGAGCAATTACTATATTACTGACTGATCGTAATCTTAATACTTCTTTTTTTGATCCTGCAGGAGGTGGAGATCCGATTTTGTATCAGCACTTGTTT-

>x300_G6YZTIE01A0PUT

--TTTGGCTAGTCAACTAGGGCATAGAGGGGGCTCTGTTGATTTAGCTATTTTTTCTTTACATTTGGCTGGGGCTTCTTCTATTTTAGGGGCAATTAATTTTATTTCTACTTGTATTAATATGCGTAATATTAGGTTAGGTATGGATCAAATGTCTTTATTTGTTTGATCAGTTTTTCTTACTGCTATTCTTTTACTTTTATCTTTACCTGTTTTAGCGGGGGCAATTACCATGCTTTTAACTGACCGTAATCTTAATACTTCTTTTTTTGACCCAAGGGGGGGTGGGGATCCTATTTTATATCAACA--------

>x301_G6YZTIE02DPZ45

--TTTAAGTTCAATTCAAAGTCATTCAGGCGCTTCCGTAGATTTAGCTATTTTTAGTTTGCATTTATCTGGAGCGTCGTCTATTTTAGGAGCAATAAATTTTATATCGACAATATTAAATATGCGCAATCCTGGACAAACATTTTATCGCTTACCGTTATTTGTTTGATCTATTTTTGTGACAGCGTTTTTGTTATTATTAGCAGTTCCAGTTTTAGCTGGTGCAATCACCATGTTACTTACTGATAGAAATTTTAATACTTCATTTTTTGATCCTGCAGGAGGAGGTGATCCAATATTATATCAACATTTATTT-

>x302_G6YZTIE01BGYMW

--ATTAAGTTCTATACAAAGCCATTCTGGAGGAGCTGTAGATTTAGCAATATTTAGCTTACACGTATCGGGAGCTTCTTCTATTTTAGGGGCTGTAAATTTCATATCAACTATTTTAAATATGAGAAGTCCTGGACAAAGCATGTATAGAATACCTTTATTTGTGTGATCTATTTTTGTGACGGCCTTCTTATTATTGTTAGCAGTACCAGTTTTAGCAGGGGCTATTACAATGCTTTTAACCGATCGTAATTTTAATACTTCGTTTTTTGATCCTGCAGGAGGTGGGGATCCAGTTTTATATCAACACTT-----

>x303_G6YZTIE01AXQB7

--ACTAAGATCAAATCTTGCTCACTCAGGGGCGTCAGTAGATTTTGCAATTTTCTCTCTCCATTTAGCCGGAATCTCGTCTTTATTGGGAGCCGTGAATTTTATTACTACACTTGGTAACCTACGAATTTTTGGCCTTTTATTAGACCGGATACCATTATTTGCGTGGTCTGTTCTTGTTACGGCGATTTTGTTGTTGTTAAGGTTACCTGTTTTAGCTGGAGCAATCACAATACTTTTAACAGATCGGAATTTAAATACCTCTTTTTATGACCCTAGCGGGGGAGGAGACCCTATTCTTTACCAGCATTTATTT-

>x304_G6YZTIE02D44F9

--TTTAAGAAGGAATCTTGCGCATTCGGGAGCTTCAGTAGATTTTGCTATTTTCTCTTTACATTTGGCAGGAATTTCGTCTCTACTAGGAGCCGTTAATTTCATTACTACTCTAGGAAATTTACGAATCTTAGGGCTTATATTAGACCGAATGCCTCTTTTTGCTTGGTCAGTTTTAGTGACAGCCATTTTACTTTTATTAAGTCTACCAGTCTTAGCTGGAGCAATTACTATGCTT---ACGGATCGTAATTTAAATACTGCTTTCTATGACCCAAGTGGAGGAGGGGACCCAATTTTGTACCAGCATTTA----

>x305_G6YZTIE01BNW8I

--TCTATCTGCAGGAATTGCCCACGCCGGCGCATCAGTTGATCTCGGAATTTTCTCACTACACCTTGCAGGTGTATCATCCATTTTAGGAGCGGTTAACTTTATGACTACAGTCATCAACATACGAACCACGGGAATGACTATAGACCGAATACCACTATTCGTCTGAGCTGTATTTTTAACAGCAATCTTACTACTTTTAAGACTTCGAGTTCTAGCCAGAGCTTTTACAATACTCTTAACAGACCGAAACTTAAATACAGCATTTTTCGACCCAGCTGGGGGAGGG---CCGATCCTATACCAACACTTATTC-

>x306_G6YZTIE02EZYQD

---CTAGCCGCAGGAACTGCTCACGCTGGAGCTTCCGTCGACCTGGGAATCTTTTCTCTACACCTTGCCGGGGTGTCTTCCATCTTAGGAGCCGTGAACTTTATTTCCACAGTAATTAACATACGCTCAACAGGTATAACGATAGACCGTATCCCACTATTTGTTTGATCAGTATTCTTAACTGCCATTCTTCTACTCCTCTCTCTTCCTGTACTTGCGGGAGCTATTACAATACTTCTAACAGATCGAAACTTAAATACATCTTTTTTCGACCCTGCGGGAGGTGGAGATCCAATCCTTTACCAACACCTATTT-

>x307_G6YZTIE02DXW2V

--GCTATCTGGGAATTTGGCCCACGCAGGGGCATCAGTAGACTTAACTATCTTCTCTCTTCATCTAGCCGGTGTCTCATCAATTCTAGGTGCAATCAACTTTATTACTACCATTATTAATATAAAACCCCCCGCTATCTCTCAATACCAAACACCTCTATTCGTGTGAGCCGTCCTAATCACCGCCGTACTTCTACTTCTCTCCCTCCCAGTGCTGGCTGCTGGCATTACCATGCTCTTAACTGACCGAAATCTTAATACCACATTCTTCGACCCCGCTGGAGGAGGAGACCCTATTCTATACCAACACCTG----

>x308_G6YZTIE02DGWHA

--CCTATCAGCAAGAATCGCACATGCGGGTGCATCTGTAGACCTGGGAATCTTCTCCCTCCACCTGGCCGGAGTCTCTTCAATCCTAGGAGCAGTTAATTTCATAAGTACCGTCTTAAACATGCGGGCAACAGGAATAACAATAGACCGGATACCTCTATTCGTGTGATCCGTATTCCTCACAGCCATCCTATTGCTCCTGTCTCTACCAGTGCTAGCCGGGGCCATCACTATACTACTAACGGACCGTAACATCAACACTTCATTCTTCGACCCAGCTGGAGGTGGAGACCCTATCCTCTATCAACACCTATTT-

>x309_G6YZTIE01B5JZ6

--TCTCTCTGCTGGAATTGCCCACGCCGGAGCCTCCGTCGACCTAGGAATTTTCTCGCTTCACCTCGCAGGTATTTCATCGATTCTAGGAGCAGTTAATTTCATAACAACGGTTATCAATATGCGGGCGACCGGAATGACTATAGACCGAATGCCCCTGTTCGTCTGAGCTGTGTTCCTGACAGCGATCCTACTCCTACTAAGACTCCCAGTCCTAGCCGGAGCTATCACTATACTCCTGACAGACCGAAACTTAAATACGGCATTCTTCGACCCCGCAGGAGGGGGGGACCCCATCTTGTACCAGCACCTGTTC-

>x310_G6YZTIE01BB5S6

----TATCAAGAAATATTGCCCACTCAGGAGCATCAGTTGATCTAACAATTTTTTCTCTTCACCTAGCAGGTTCCTCGTCTATTTTAGGGGCTATTAATTTTATATCAACAGTTATTAATATACGACCTAATAGTATATCCTTTGACCGAATTCCTTTATTCGTCTGAAGTGTCTTTTTAACAGTGATTCTCCTTTTACTCTCACTACCCGTTTTAGCAGGAGCTATTACTATACTTCTTACTGACCGAAACCTTAATACATCGTTCTTCGATCCAACAGGAGGAGGTGACCCTATTCTTTATCAACACCTATTT-

>x311_G6YZTIE01B4N2Z

--ATTAAGTTCAATTCAAAGTCATTCAGGGGCTTCAGTAGATTTAGCTATTTTTAGTTTACATTTATCGGGTGCTTCATCAATTCTAGGAGCAATTAATTTTATTTCTACTATTTTAAATATGCGTAGTATTGGTCAAAATTTTTATAGAATACCATTATTTGTTTGATCAATTTTTATAACTGCGATACTACTATTATTAGCGGTTCCAGTTTTAGCAGGAGCCATTACTATGTTATTAACTGATAGAAATTTTAATACTACGTTT---GATCCTGCAGGAGGAGGTGATCCTATTTTATATCAACATTTATTT-

>x312_G6YZTIE01BXNPI

---CTCAGGAGAAATATTGCCCACTCTGGAGCCTCAGTTGATTTTGCTATTTTTTCTCTTCATTTAGCGGGTATCTCTTCTTTACTGGGAGCTGTGAATTTTATTAGAACCCTAGGAAACCTACGGGTATTAGGCTTAGTTTGAGACACTGTTCCCTTATTTGGTTGGGCAGTATTTATTACTGCAATTCTTTTGTTGCTTTCTCTACCTGTCTTAGCGGGGGCTATTACTATGCTTCTGACCGACCGTAATCTTAACACCACATTTTATGACGCGGCTGGAGGAGGCGACCCTGTCCTTTACCAGCATTTA----

>x313_G6YZTIE02EXLCJ

--TTTAAGAAGTCATTTGTTCCACGGAGGTCCTTCTGTAGATTTCGCAATCTTTAGGCTACACTTAGCCGGGGTTTCCTCTCTCCTAGGAGCAATTAATTTTATTACAACAATTCTTAATTTACGAGTGTTAGGAATGATCCTTGAGCGAATACCTTTGTTCCCTTGGGCTGTCCTAGTCACAGCAGTCTTACTCCTGTTATCCCTGCCCGTCCTTGCCGGAGCCATCACTATGCTGTTAACAGATCGAAATCTCAACACTTCGTTCTACGACCCTATAGGGGGAGGAGATCCAATTCTTTATCAACATTTATTC-

>x314_G6YZTIE01BD9KR

--ACTCTCTAATGCTATCGCCCATGCAGGACCATCAGCCGACATAGCAATTTTTTCACTTCACCTAGCAGGTGCATCTTCATTCATAGGCGTCATCAATTTTATAAGCACAGTAGTTAATATACGCCCAAAAGGCATGGCAGTAATGTGTATACCACTATTTGTCTGAGCAGCATTTTTAACAGCTATTCTTCTTCTCCTATCCTTGCCCATATTAGCAGGTGCCATTACCATACTTCTAACCGATCGTAATTTAAACACCTCTTTTTTCG---------------------------------------------

>x315_G6YZTIE01A4OKB

--TCTAAGCTCTAACCTTGCCCACGCAGGTGCCTCCGTGGATTTCGCTATTTTCTCTTTACATCTAGCAGGTATTTCTTCCCTACTCGGAGCTGTAAATTTTATTAGAACACTAATTAATCTTCGGTCTTTCGGGATAAGACTAGAGAGAATACCATTATTCGCTTGGGCCGTCTTTATTACAGCCATTCTTCTTCTGTTATCCCTCCCAGTGCTCGCTGGAGCCATTACTATGCTTCTCACTGACCGTAATTTGAACACAGCTTTTTATGATGTAGCGGGAGGGGGCGATCCTGTTTTATACCAGCATTTGTTT-

>x316_G6YZTIE02C4I2L

--CTTATCAGGTAATGTAGCACATGCAGGACCAGCAGTAGACCTGACAATTCTATCTCTCCATCTTGCTGGTGTATCTTCTCTTATAGGCGCAATAAATTTTACTACAACTATTCTCAACAGACGAATAGAAGGAATACCTACAGAAAAAATACCACTATTTATTTGATCAGTACTTATTACAGTTATCCTACTAATTCTTGCACTCCCTGTACTCGCAGGTGCACTTACTATACTAATTATAGACCGTAACTGTAACACATCCTTCTTCGAACCTACAGGAGGAGGAGATCCAATTCTCTTCCAACACTTATTC-

>x317_G6YZTIE01CENHY

---CTGTCAGCAAGAATTGCTCACGCGGGAGCGTCAGTAGACCTTGGAATTTTCTCATTACACCTAGCAGGTGTGTCTTCAATCCTAGGAGCTATCAACTTTATAACAACTGTCATCAACATACGAGCGACAGGTATTACCATAGACCGAATGCCTTTATTTGTATGAGCAGTGTTCCTCACCGCTATCCTCCTACTCTTATCACTACCAGTTCTAGCTGGCGCTATTACTATACTTCTAACTGATCGTAATTTAAATACTTCATTCTTTGAGCCAAGAGGTGGTGGAGATCCAATTCTTTACCAACATTTATTC-

>x318_G6YZTIE02C7X4Q

----TTAGGAGAAATGTCGCTCACTCAGGCCCATCTGTAGATTTTGCTATTTTTGCTTTACACCTTGCAGGAGTTTCCTCACTTTTAGGTGCAGTAAATTTTATCAGCACTTTTTCTAATTTGCGTACACTCGGAATATTACTAGAGCGAGTGCCACTTTTTGGTTGAGCTGTTTTAATCACTACAATTCTTCTTCTTCTATCTCTGCCAGTATTGGCGGGCGCTATTACTATGCTTCTTACTGACCGAAATTTGAATACTTCATTCTACGACTCCAACGGGGGGGGTGACCCTACTTTATACCAGCACCTATTT-

>x319_G6YZTIE02DAXPY

--CCTATCAGCAAGAATCGCCCACGCAGGTGCATCCGTAGACCTGGGAATCTTCTCCCTCCACTTAGCTGGAGTGTTCTCAATCCTAGGAGCAGTAAATTTCATAAGTACCGTCTTAAACATACGAGCAACAGGCATAACAATAGACCGAATGCCACTATTCGTATGATCTGTATTCCTCACAGCCATCCTCCTTCTACTATCCCTACCTGTGCTAGCTGGAGCTATCACTATACTACTAACAGACCGTAACATTAGCACTCCATTCCTCGACCCAGCAGGGAGTGGTGACCCGATCCTCTATCAACATCTATTC-

>x320_G6YZTIE01CCC3B

--TTTAGCGGCAGAGACAGCCCATAGAGGGGGCGCTGTAGATATCGCT---TTTTCTCTACATTTAGCGGGAGCCTCCTCTATCTTAGGAGCCATTAATTTTATTTCTACTGTTATCAACATACGGGCTCCGGCTATAACCATAGACCAAATCCCTTTATTCGTTTGGTCTATCTTTTTCACTACAATCCTACTCCTTCTCTCGCTCCCCGTACTAGCAGGAGCTATTACTATACTTCTTACTGACCGTAACCTCAATACCTCTTTTTTCGACCCGAGAGGAGGAGGAGACCCTATCCTGTACCAGCACTTATTC-

>x321_G6YZTIE01BB5F1

--CTTATCCGGCAATCTAGCCCACGCGGGAGCCTCTGTAGACTTAACCATCTTCTCCTTACACCTAGCCGGGATCTCTTCAATCCTTGGAGCAATCAACTTTATTACAACTATCATTAACATGAAACCCCCTGCCATCTCCCAATACCAAACACCCCTATTCGTGTGAGCAGTTCTAATTACTGCCATTCTCCTTCTCCTCTCCCTTCCGGTCTTAGCCGCTGGCATCACCATGCTCTTAACAGACCGCAATCTAAACACCACATTCTTTGACCCTGCAGGAGGGGGAGACCCAATCCTTTACCAACACTTA----

>x322_G6YZTIE02DKVMA

--CCTAGCCGGCAACCTCGCACATGCAGGAGCTTCTGTTGATCTGACAATTTTCTCTCTTCATCTTGCAGGAATTTCTTCTATTCTGGGGGCCATTAACTTTATTACGACAATTATTAACATGAAACCTCCAGCTATCTCCCAGTATCAAACCCCATTATTCGTCTGAGCAGTCCTAATTACGGCTGTACTTTTACTCCTTTCTCTCCCAGTCTTGGCTGCCGGCATCACAATGCTTCTCACAGACCGAAATCTAAACACAACCTTCTTCGACCCCCGC-------------------------------------

>x323_G6YZTIE02ENA8M

--CCTTAGAAGAAATATCGCTCACTCCGGGCCCTCCGTTGATTTTGCCATTTTTAGGCTTCATTTGGCAGGGGTTTCGTCACTCCTAGGGGCTGTTAACTTTATTAGAACTCTTAGAAATCTACGTACACTAGGGATACTACTGGATCGTATGCCCCTTTTTGCCTGAGCTGTTTTAATTACTGCTATTTTACTTCTTTTATCTCTCCCCGTGTTGGCAGGAGCAATCACTATATTGTTAACAGACCGTAATCTTAACACATCCTTTTATGACCCGAGAGGTGGAGGTGACCCTGTATTGTATCAACATTTATTT-

>x324_G6YZTIE02EH38V

----TATCTTCAAGCCTTGCCCACGCCGGGCCCTCTGTAGATTTAGCTATTTTTTCCCTTCACCTAGCAGGAGCATCTTCTATCTTAGGCTCCATCAATTTCATCTCAACTATTATAACCGCACGCCAAGAAGCCTACACTCTCGTGCGCATGCCCCTTTTTCTATGAGCAGTAAAAATTACTACTATCTTACTTCTTCTCTCACTGCCAGTCCTTGCAGGAGCTATCACCATACTTCTTACAGATCGCAATGCTAATACATCCTTCTTCGACCCAAGAGGCGGGGGAGACCCTGTGTTGTTTCAACACCTATTC-

>x325_G6YZTIE02DXW5M

---TTGTCCAGCAACCTGGCTCACGCCGGAGCTTCAGTCGACCTAACGATCTTTTCTTTACACCTAGCAGGTATTTCTTCAATTCTAGGAGCAATTAACTTTATCACCACAATCGTCAATATGAAGCCCCCCGCCATCTCCCAGTATCAAACGCCTCTTTTCGTATGAGCAGTGCTAATCACAGCCGTTCTTCTTCTTCTGTCTTTGCCTGTTCTAGCCGCAGGAATTACAATGCTCCTGACGGACCGAAATTTAAATACAACATTTTTTGACCCTGCAGGGGGAGGAGACCCCATTCTCTATCAACACCTC----

>x326_G6YZTIE01BXHGG

-CTCTGGCTGCAGCCACTGCCCACGCAGGGGCCTCGGTGGACCTTGGGATTTTTTCTCTTCACTTAGCAGGTGTGTCATCCATTCTAGGTGCTGTAAATTTTATAACTACTGTGATTAATATACGATCTTACGGTATGACATTAGATCAAATACCTTTATTTGTGTGAGCGGTTTTTATTACTGCTATCTTACTCTTACTCTCATTACCAGTTTTAGCAGGTGCTATTACTATACTCTTAACTGACCGAAATTTAAACACTTCGTTCTTTGACCCAGCGGGGGGAGGTGATCCTGTTTTATACCAGCACCTTTT--

>x327_G6YZTIE01BW5FV

--ATTAAGTAGTAATTTAGCGCATTCAGGAATATCAGTAGACTTTGCTATTTTCTCTTTGCATTTAGCTGGTATTTCTTCTCTCTTAGGAGCTATTAACTTTATTAGTACAATTATAAATTTACGAGTTTTAGGAATGACTTTAGATAACATTCCTTTATTTGTCTGAGCGGTATTTATTACTGCTATCCTTTTATTATTATCTCTCCCAGTTTTAGCAGGAGCAATTACAATATTATTAACTGATCGTAATTTAAATACTTCTTTTTATGACTCTAGAGGAGGAGGAGATCCTGTGCTTTATCAACATCTGTTT-

>x328_G6YZTIE02DG1VU

---TTAGCTAGCATCCAAGCACACTCAGGTGGGTCAGTTGATATGGCCATTTTTAGTTTACATTTAGCTGGGGCATCTTCTATATTGGGGGCAATAAATTTTATAACTACTATATTTAATATGAGGGCACCAGGAGTAACTATGGATAAATTGCCATTATTTGTCTGATCTGTACTTATTACAGTCTTTTTGTTATTATTGGCTTTACCTGTATTAGCAGGAGCAATAACTATGTTATTAACGGATAGAAATTTTAATACTACTTTCTTTGACCCTGCAGGAGGAGGTGATCCAGTTTTATATCAACATTTATTT-

>x329_G6YZTIE02EUXHH

CGG---TCTGCTGCAATCGCCCACGCCGGGGCATCAGTAGATTTAGGTATTTTCTCACTTCATCTTGCAGGTGTGTCTTCAATCCTAGGTGCAGTTAATTTCATAACTACTGTAATTAACATACGACCCCAAGGTATAACAATAGACCGTATGCCACTCTTCGTATGAGCAGTCTTCATTACTGCTATTTTATTACAACTTTCTCTCCCAGTATTAGCAGGAGCTATTACCATGCTCCTAACAGATCGAAACCTAAACACCTCTTCATTCGACCCCGCTGGGG---------------------------------

>x330_G6YZTIE01CGREY

---CTGTCCGGAAACTTAGCCCACGCAGGAGCCTCTGTAGACTTAACTATCTTCTCCCTACACCTAGCAGGTATTTCCTCAATCCTAGGGGCAATCAATTTTATTACAACTATTATTAACATGAAACCCCCTGCTATCTCTCAATACCAAACACCCTTATTCGTATGAGCCGTCCTAATTACAGCCGTACTCCTACTTCTCTCGCTCCCAGTCTTAGCCGCTGGAATCACTATACTACTAACCGACCGAAACCTAAACACAACTTTCTTTGACCCAGCAGGAGGTGGTGACCCCATTCTTTACCAACACCTA----

>x331_G6YZTIE02D2DUC

--TCTAGCTGCGGGCATTGCCTACGCTGGAGCATCTGTAAATATTGGAATCTTCTCATTTCACCTAGCGGGGGTGTCGTCAATTCTAGGGGCCGTTAACTTCATGACCACCGTAATTAATATACGAGCCACTGGTATGTCGATGGACCGCATGCCTCTCTTCGTCTGATCAGTTTTCCTTACTGCTATCTTTCTT---CTTTCTCTCCCTGTTCTTGCCGGGGCGATCACTATGCTCCTAACCGACTGCAACCTAAATACCTCTTTCTTTGACCCTGCGGGAGGTGGCGACCCAAATCTCTATCAACACTTA----

>x332_G6YZTIE02D7NOB

--ACTCTCTGCTGGAATTGCCCATGCAGGAGCTTCTGTCGACCTAGGAATTTTCTCGCTACACCTCGCAGGTGTCTCATCGATTTTAGGAGCGGTTAATTTCATGACAACCGTCATCAACATACGAGCAACCGGAATAACCATAGACCGAATACCCCTGTTCGTCTGGGCTGTCTTCCTAACAGCAATCCTACTCCTACTAAGACTCCCAGTTCTAGCCGGAGCTATCACTATGCTCCTGACAGACCGAAACCTGAATACAGCACTCTTCGACCCGGCCGGGGGAGGCGACCCCATCTTATACCAACACCTGTTC-

>x333_G6YZTIE01AYW8U

--TCTCTCTAATGCTATTGCTCATGCAGGTCCATCAGTTGACATAGCAATTCTTTCACTCCACTTAGCGGGAGCATCTTCAGTCATAGGTGCCATCAACTTCATATGCACAGTAATTAATATACGCCCAAAAGGAATGTCAGCAACATGCATACCACTGTTTGTATGAGCAGTATTTTTAACAGCCATTCATCTCCTTTTATCCTTACCTGTATTAGCAGGGGCCATTACCATACTTTTAACCGATCGTAACTTAAACACTTCCTTTTTCGACCCAGTAGGAGGGAGAGACCCAATCCTCTACCAACACCTATTT-

>x334_G6YZTIE01BRMHG

--TCTTTCGGCAGGGATTGCACACGCTGGGGCTTCAGTAGACATAGGAATCTTTTCTCTTCATATTGCTGGTGCTTCATCTATTCTAGGAGCTGTTAACTTCATTACGACAGTCATCAACATACGCTCAGCTGGAATGACGATGGACCGTGTGCCTTTATTTGTGTGGTCTGTGTTTATTACTGCCATCCTTTTACTATTGTCGCTTCCTGTCCTAGCCGGAGCTATCACTATGCTTTTAACTGACCGTAATTTAAACACTTCTTTTTTCGATCCAGCGGGAGGTGGAGACCCCGTTTTATACCAACACTTATTC-
